# Intrinsic Dynamic and Static Nature of π···π Interactions in Fused Benzene-Type Helicenes and Dimers, Elucidated with QTAIM Dual Functional Analysis

**DOI:** 10.3390/nano12030321

**Published:** 2022-01-19

**Authors:** Taro Nishide, Satoko Hayashi

**Affiliations:** Faculty of Systems Engineering, Wakayama University, 930 Sakaedani, Wakayama 640-8510, Japan; s209004@wakayama-u.ac.jp

**Keywords:** ab initio calculations, quantum theory of atoms-in-molecules (QTAIM), nonbonded interactions, polycyclic aromatic hydrocarbon (PAH)

## Abstract

The intrinsic dynamic and static nature of the π···π interactions between the phenyl groups in proximity of helicenes **3**–**12** are elucidated with the quantum theory of atoms-in-molecules dual functional analysis (QTAIM-DFA). The π···π interactions appear in C-∗-C, H-∗-H, and C-∗-H, with the asterisks indicating the existence of bond critical points (BCPs) on the interactions. The interactions of **3**–**12** are all predicted to have a *p*-CS/vdW nature (vdW nature of the pure closed-shell interaction), except for ^2^C_bay_-∗-^7^C_bay_ of **10**, which has a *p*-CS/*t*-HB_nc_ nature (typical-HBs with no covalency). (See the text for definition of the numbers of C and the bay and cape areas). The natures of the interactions are similarly elucidated between the components of helicene dimers **6**:**6** and **7**:**7** with QTAIM-DFA, which have a *p*-CS/vdW nature. The characteristic electronic structures of helicenes are clarified through the natures predicted with QTAIM-DFA. Some bond paths (BPs) in helicenes appeared or disappeared, depending on the calculation methods. The static nature of C_cape_-∗-C_cape_ is very similar to that of C_bay_-∗-C_bay_ in **9**–**12**, whereas the dynamic nature of C_cape_-∗-C_cape_ appears to be very different from that of C_bay_-∗-C_bay_. The results will be a guide to design the helicene-containing materials of high functionality.

## 1. Introduction

Helicenes, which are ortho-fused polycyclic aromatic or heteroaromatic compounds with all rings angularly arranged to form helically shaped molecules, are of current and continuing interest. Helicenes are chiral; as a result, they are expected to have specific functionalities. Recently, helicenes have been widely applied in various fields [1,2,3,4,5], such as organic semiconductors [6,7,8,9,10], asymmetric catalysis [11,12,13,14,15,16], and molecular recognition [17,18,19,20,21,22], due to their diverse functionalities in materials. Many studies have also been reported on self-assembly phenomena at metal surfaces [23,24,25,26] caused by interactions with the π-orbitals of helicenes. The π-orbitals will cause intramolecular π···π interactions between adjacent aromatic rings of helicenes, which play an important role in effective interactions. It is crucial to clarify the nature of π···π interactions for future high-functioning material developments based on helicenes. The discussion in this paper will be limited to π···π interactions between the aromatic rings, the nature of which needs to be clarified, since helicenes of the fused benzene type were chosen as the target.

The noncovalent distances between the aromatic planes in close proximity to the helicenes were determined as the total effect of the attractive and repulsive forces between the atoms on the planes. The restoring forces from the deviated planarity in the helicenes should be a main factor for the attractive and repulsive forces due to π-orbital overlapping in the helicenes. The noncovalent distances between the planes in close proximity to the helicenes are defined as the balanced distances of the two factors. The noncovalent intramolecular distances between atoms in close proximity to the helicenes must be (much) shorter than the noncovalent intermolecular distances between the unrestricted nonhelical aromatic species. The shorter distances in helicenes result from the π···π interactions between the planes in close proximity in space, which operate under very severe conditions. Clarifying the nature of the π···π interactions in helicenes under such severe conditions will enable us to understand the factors that control the structures and the nature of the interactions. The results will also provide a starting point for understanding the nature of π···π interactions and will hint at designs for materials with high functionality based on the interactions.

**Scheme 1:**
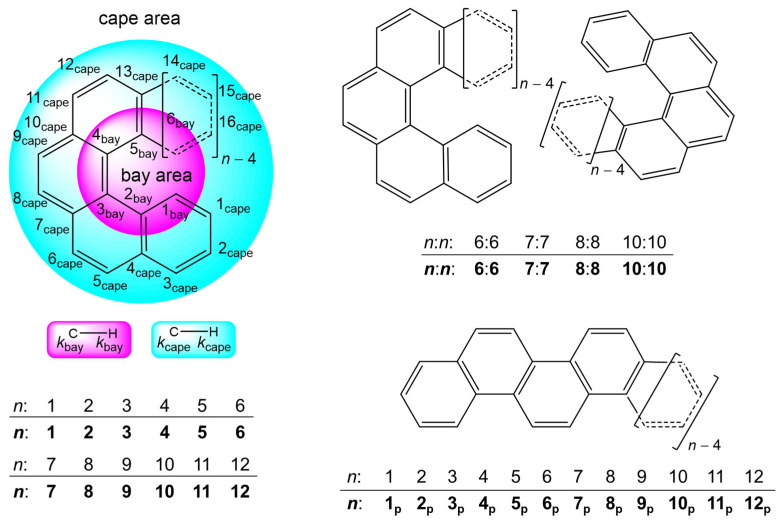
Helicenes, **1**–**12**, dimers, **6**:**6**–**8**:**8** and **10**:**10**, and [*n*]phenacenes, **1_p_**–**12_p_**. The bay and cape areas in **1**–**12** are illustrated. The number of C is shown, where the number of H is the same for C–H. Benzene, naphthalene, and phenanthrene are defined corresponding to *n* = 1, 2, and 3, respectively.

We have been particularly interested in the π···π interactions that operate under severe conditions, as these should be the factors that control the fine details of the structures. Interactions are also expected to result in materials with high functionalities. The nature of π···π interactions under such severe conditions was investigated in a series of fused benzene-type helicenes **1**–**12** and concave-type dimers **6**:**6**–**8**:**8** and **10**:**10**, where **1**–**3** are analyzed as helicenes in this paper, although they are usually not. Scheme 1 shows the structures of helicenes **1**–**12**, dimers **6**:**6**–**8**:**8**, **10**:**10**, and [*n*]phenacenes **1_p_**–**12_p_**, where **1_p_**–**12_p_** are the comparative compounds and **p** stands for phenacenes. The bay and cape areas used in this paper are also illustrated. We have previously reported the nature of the benzene π···π interactions in cyclophanes [27] (see also [28,29]). The π···π interactions in the helicenes must correspond to the extended π···π interactions of the species.

The π···π interactions in the helicenes were analyzed with QTAIM dual functional analysis (QTAIM-DFA [30,31,32,33,34,35]), which we proposed based on the QTAIM approach introduced by Bader [36,37]. The π···π interactions will be reproduced on the bond paths (BPs) between atoms, where a bond critical point (BCP, ∗) appears on each BP. The π···π interactions in helicenes are typically described by BPs with BCPs of the H-∗-H, C-∗-H, and C-∗-C forms. The asterisk indicates the existence of a BCP in each BP [36,37]. In QTAIM-DFA, *H*_b_(***r***_c_) is plotted versus *H*_b_(***r***_c_)–*V*_b_(***r***_c_)/2, where *H*_b_(***r***_c_) and *V*_b_(***r***_c_) are the total electron energy densities and potential energy densities, respectively, at the BCPs of the interactions in question. In our treatment, data from the fully optimized structures and the perturbed structures surrounding the fully optimized structures are used for the plots.

Data from the fully optimized structures in the plots were analyzed using polar coordinate (*R*, *θ*) representation, which corresponds to the static nature of the interactions [30,31,32,33,34,35]. Data from both the perturbed and fully optimized structures are expressed by (*θ*_p_, *κ*_p_), where *θ*_p_ corresponds to the tangent line and *κ*_p_ is the curvature of the plot. *θ* and *θ*_p_ are measured from the *y*-axis and the *y*-direction, respectively. (See Figure SA1 of the Appendix S1 of the Supporting Information for the definition of the QTAIM-DFA parameters of (*R*, *θ*) and (*θ*_p_, *κ*_p_), along with Equations (SA3)–(SA6) and the footnotes of Table 1). The concept of the dynamic nature of the interactions was proposed based on (*θ*_p_, *κ*_p_). The *θ*_p_ and *κ*_p_ for the major bonds seem to be controlled by the characters of the local bonds in question: The influence from the behaviors of the minor bonds would not be so severe for usual cases.

The perturbed structures necessary for QTAIM-DFA were generated by CIV [38], with the coordinates **C***_i_* corresponding to the compliance constants *C_ii_* for the internal vibrations [39,40,41,42,43,44]. The basic concept for the compliance constants was introduced by Taylor and Pitzer [45], followed by Konkoli and Cremer [46]. The *C_ij_* are defined as the partial second derivatives of the potential energy due to an external force [47,48,49], where *i* and *j* refer to internal coordinates. The dynamic nature of the interactions based on perturbed structures with CIV is described as the “intrinsic dynamic nature of interactions” because the coordinates are invariant to the choice of coordinate system. QTAIM-DFA and the criteria obtained by applying QTAIM-DFA with CIV to standard interactions are explained in the Appendix S1 of the Supporting Information using Schemes SA1–SA3, Figure SA1 and SA2, Table SA1, and Equations (SA1)–(SA7).

In this work, we present the results of the investigations into the natures of the π···π interactions in **1**–**12**, **6**:**6**–**8**:**8**, and **10**:**10**, although some are discussed in the Supporting Information or calculated only for comparison. The interactions are classified and characterized by using the criteria as a reference. The structural features and the energy profile are also discussed to provide a solid basis for the discussion.

## 2. Methodological Details of the Calculations

Calculations were performed with the Gaussian 09 program package [50]. The 6-311+G(3d,p) basis set was used for the calculations at the DFT level of M06-2X [51] (M06-2X/6-311+G(3d,p)). The optimized structures were confirmed by frequency analysis. The results of the frequency analysis were used to calculate the compliance constants (*C_ii_*) and the coordinates corresponding to *C_ii_* (**C***_i_*). Calculations were also performed with M06-2X/6-311+G(2d,p) and LC-ωPBE/6-311+G(2d,p) [52] to examine the basis set and level dependence, containing the optimized π···π distances, on the results. The results with M06-2X/6-311+G(3d,p) are discussed in the text, while the results with M06-2X/6-311+G(2d,p) and LC-ωPBE/6-311+G(2d,p) are discussed mainly in the Supporting Information. We should be careful with the basis set and level dependence on the QTAIM-DFA parameters, which has been examined carefully [53]. Similar methodology was also employed for the theoretical studies of the π-stacking [54,55].

Equation (1) explains the method for generating perturbed structures with CIV [38]. The *i*-th perturbed structure in question (**S***_iw_*) is generated by adding **C***_i_* to the standard orientation of a fully optimized structure (**S**_o_) in the matrix representation. The coefficient *g_iw_* in Equation (1) controls the structural difference between **S***_iw_* and **S**_o_, *g_iw_* is determined to satisfy Equation (2) for *r*, where *r* and *r*_o_ stand for the interaction distances in question in the perturbed and fully optimized structures, respectively, with *a*_o_ = 0.52918 Å (Bohr radius). Five-digit **C***_i_* values were used to predict **S***_iw_*.

**S***_iw_* = **S**_o_ + *g_iw_*·**C***_i_* (1)

*r* = *r*_o_ + *wa*_o_ (*w* = (0), ±0.025 and ±0.05; *a*_o_ = 0.52918 Å) (2)

*y* = *c*_o_ + *c*_1_*x* + *c*_2_*x*^2^ + *c*_3_*x*^3^ (*R*_c_^2^: square of the correlation coefficient) (3)

The QTAIM functions were calculated using the same basis set system as in the optimizations, unless otherwise noted, and were analyzed with the AIM2000 [56,57] and AIMAll [58] programs. The *H*_b_(***r***_c_) values are plotted versus the *H*_b_(***r***_c_) − *V*_b_(***r***_c_)/2 values for five data points in Equation (2) in QTAIM-DFA: *w* = 0, ±0.025, and ±0.05. Each plot was analyzed using a cubic function regression curve, as shown in Equation (3), where (*x*, *y*) = (*H*_b_(***r***_c_) − *V*_b_(***r***_c_)/2, *H*_b_(***r***_c_)) (*R*_c_^2^ > 0.99999 as usual) [31].

## 3. Results and Discussion

### 3.1. Structural Features of ***1**–**12*** and Their Energy Profile

The structures of **1**–**12** were optimized with M06-2X/6-311+G(3d,p), M06-2X/6-311+G(2d,p), and LC-ωPBE/6-311+G(2d,p), retaining *C*_2_ symmetry. The selected noncovalent X···Y distances (X, Y = C, H) in the optimized structures with M06-2X/6-311+G(3d,p), M06-2X/6-311+G(2d,p), and LC-ωPBE/6-311+G(2d,p) are shown in Table S1 of the Supporting Information, along with the observed values [59,60,61,62,63,64,65,66].

How can the behaviour of the energies of the helicenes be explained? The energies of the helicenes were compared with the energies of [*n*]phenacene, a nonhelical species, evaluated with M06-2X/6-311+G(3d,p). The energy profiles will be discussed based on the energy differences, Δ*E*(***n***) = *E*(***n***) − *E*(***n*** − 1) for helicenes (**1**–**12**) and Δ*E*(***n*_p_**) = *E*(***n*_p_**) − *E*(***n*_p_** − 1) for [*n*]phenacenes (**1_p_**–**12_p_**). The Δ*E*(***n***) values correspond to the energy differences in the formation of ***n*** from ***n*** − 1, and the Δ*E*(***n*_p_**) values similarly correspond to ***n*_p_** from (***n*_p_**− 1). The *E*(***n***), *E*(***n*_p_**), Δ*E*(***n***), and Δ*E*(***n*_p_**) values were calculated on the energy surface, which are described by *E*_ES_(***n***), *E*_ES_(***n*_p_**), Δ*E*_ES_(***n***), and Δ*E*_ES_(***n*_p_**), respectively. The values were also calculated with the zero-point energies, which are described by *E*_ZP_(***n***), *E*_ZP_(***n*_p_**), Δ*E*_ZP_(***n***), and Δ*E*_ZP_(***n*_p_**). The values calculated with M06-2X/6-311+G(3d,p) are collected in Table S2 of the Supporting Information. The plot of Δ*E*_ZP_(***n***) versus Δ*E*_ES_(***n***) revealed an excellent correlation (*y* = 1.0042*x* + 0.6859; *R*_c_^2^ = 0.980, see Figure S1 of the Supporting Information). As a result, Δ*E*_ES_(***n***) can be used to analyze the energy terms.

**Figure 1:**
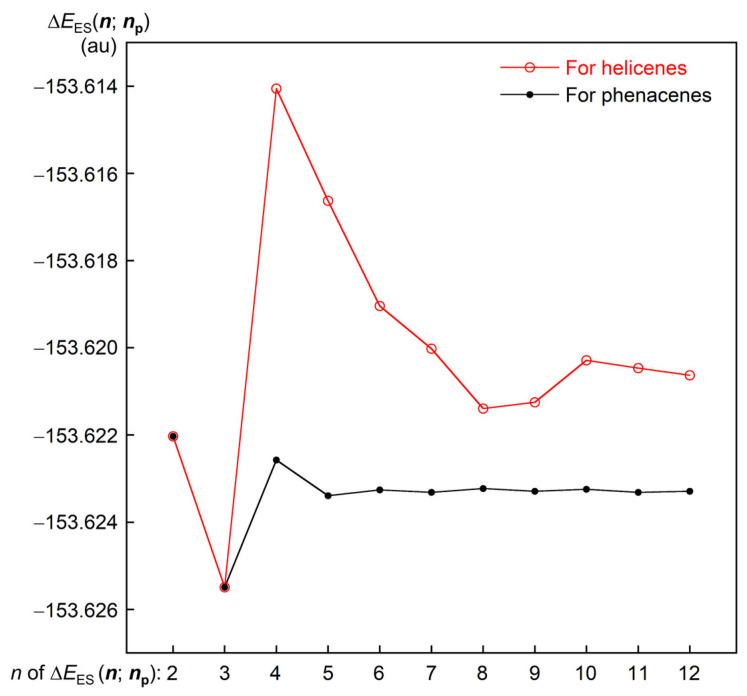
Plot of Δ*E*_ES_(***n***) and Δ*E*_ES_(***n*_p_**) versus *n*, evaluated with M06-2X/6-311+G(3d,p), where Δ*E*_ES_(***n***) = *E*_ES_(***n***) − *E*_ES_(***n*** − 1) and Δ*E*_ES_(***n*_p_**) = *E*_ES_(***n*_p_**) − *E*_ES_(***n*_p_** − 1).

Figure 1 shows the plots of Δ*E*_ES_(***n***) and Δ*E*_ES_(***n*_p_**) versus *n*. Both the Δ*E*_ES_(***n***) and Δ*E*_ES_(***n*_p_**) values (Δ*E*_ES_(***n***; ***n*_p_**)) decrease when *n* increases from 2 to 3. The extension of the π system appears to contribute more to the formation of phenanthrene from naphthalene than the repulsive noncovalent H···H interaction. The Δ*E*_ES_(**3**; **3_p_**) values are less than the Δ*E*_ES_(**2**; **2_p_**) values; however, the Δ*E*_ES_(***n***; ***n*_p_**) values increase from **3**; **3_p_** to **4**; **4_p_**. In the case of Δ*E*_ES_(***n*_p_**), the Δ*E*_ES_(**4_p_**) value is somewhat larger than Δ*E*_ES_(**3_p_**) but slightly smaller than Δ*E*_ES_(**2_p_**). The Δ*E*_ES_(***n*_p_**) value decreases again slightly from **4_p_** to **5_p_**. Then, the values are nearly constant for ***n*_p_** ≥ **5_p_**. The results show that the repulsive energy from the noncovalent H···H interaction does not appear to be as severe as the stabilization factor from the extended π systems in **1_p_**–**12_p_**. Namely, the ***n*_p_** system stabilizes almost constantly as the size of the species increase, especially for ***n*_p_** ≥ **5_p_**, although a change in Δ*E*_ES_(***n*_p_**) is detected for **2_p_** ≤ ***n*_p_** < **5_p_**.

**Figure 2:**
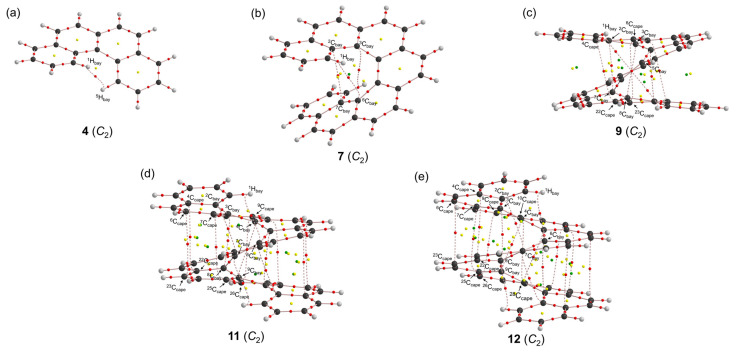
Molecular graphs for **4** (**a**), **7** (**b**), **9** (**c**), **11** (**d**), and **12** (**e**), calculated with M06-2X/6-311+G(3d,p), where BPs with BCPs corresponding to intramolecular noncovalent interactions are detected. The BCPs are denoted by red dots, RCPs (ring critical points) by yellow dots, CCPs (cage critical points) by green dots, and BPs by pink lines. The carbon atoms are in black and the hydrogen atoms are in grey.

The data points for Δ*E*_ES_(***n***) appear to be greater than those for Δ*E*_ES_(***n*_p_**) when *n* ≥ 4. The observations must be due to the severe steric repulsion in Δ*E*_ES_(***n*** ≥ **4**), where the plot for Δ*E*_ES_(***n*_p_**) corresponds to that without such severe steric repulsion. The Δ*E*_ES_(**4**) value is much larger than those of Δ*E*_ES_(**2**) and Δ*E*_ES_(**3**). The results can be explained by considering the much larger contribution from the repulsive noncovalent H···H interaction in **4** than in **3**. This consideration is supported by the optimized structure of **4**, drawn in Figure 2, as the molecular graph type. The Δ*E*_ES_(***n***) values decrease in the following order: Δ*E*_ES_(**4**) > Δ*E*_ES_(**5**) > Δ*E*_ES_(**6**) > Δ*E*_ES_(**7**) > Δ*E*_ES_(**8**). The contribution of steric repulsion to Δ*E*_ES_(***n***) due to noncovalent interactions is expected to increase as *n* increases in this process. However, the observed results are the opposite of what was expected. Therefore, the observed trend should be attributed to the increased energy-lowering effect by the extended π systems in **4**–**8** relative to the repulsive interactions.

The Δ*E*_ES_(***n***) value becomes somewhat larger again from ***n*** = **8** to **9** and **9** to **10**, and then decreases again from **10** to **11** and **11** to **12**. The subtle conditions in the steric repulsion contribute to the complex behaviour of Δ*E*_ES_(***n***) (8 ≤ *n* ≤ 12). The behaviour of Δ*E*_ES_(**2**)–Δ*E*_ES_(**12**) shown in Figure 1 should be affected both by the repulsive factor of the noncovalent H-∗-H, C-∗-H, and C-∗-C interactions and by the energy-lowering factor of the extended π system. The Δ*E*_ES_(**4**) value is the largest among Δ*E*_ES_(**2**)–Δ*E*_ES_(**12**). The results are of great interest since the repulsive noncovalent H···H interaction in **4** from **3** appears to be very large among **2**–**12** when evaluated by Δ*E*_ES_(***n***). The trend in Δ*E*_ES_(***n***) seems to be in good agreement with those reported by Rulíšek et al., calculated with PBE-D/TZVP//PBE-D/6-31G(d), except for Δ*E*_ES_(**8**) and Δ*E*_ES_(**9**) [67].

It is also instructive to analyze the aromaticities of acenes, phenacenes, and helicenes after investigating the energy profiles. The structures of acenes, phenacenes, and helicenes are illustrated in Chart S1 of the Supporting Information, together with the definition of the ring positions. The aromaticities were analyzed by the HOMA (harmonic oscillator model of aromaticity) method [68]. The HOMA values are collected in Table S3 of the Supporting Information. The HOMA values of the acenes and phenacenes are plotted versus those of the helicenes, which are shown in Figure S2 of the Supporting Information. The plot of the data for phenacenes versus those for helicenes gave a very good correlation (*y* = 0.964*x* + 0.042; *R*_c_^2^ = 0.981), whereas the correlations of the plots for acenes versus helicenes were very poor (*y* = −0.685*x* + 0.995; *R*_c_^2^ = 0.492 if calculated under the closed–shell singlet conditions and *y* = −0.399*x* + 0.880; *R*_c_^2^ = 0.317 under the open–shell singlet conditions). The very good correlation of the former demonstrates that the aromaticities of the helicenes appear to be very similar to those of the phenacenes, irrespective of the very severe steric deformations in the structures of helicenes. However, the very poor correlations with the negative correlation constants show that the aromaticities of the helicenes are very different from those of acenes.

### 3.2. Survey of X-∗-Y (X, Y = C and H) in ***3**–**12*** with the Molecular Graphs

Figure 2 shows the molecular graphs, exemplified by **4**, **7**, **9**, **11**, and **12**. Many BPs with BCPs are detected in the π···π interactions between the phenyl rings in close proximity to the helicenes. The molecular graphs for helicenes **3**–**12**, except for **4**, **7**, **9**, **11**, and **12**, are shown in Figure S3 of the Supporting Information.

The BPs corresponding to X-∗-Y (X, Y = C and H) appear almost straight, as shown in Figure 2 and Figure S4 of the Supporting Information, although some appear somewhat bent. To examine the linearity of the BPs further, the lengths of the BPs (*r*_BP_) were calculated for all X-∗-Y of **3**–**12**, along with the corresponding straight-line distances (*R*_SL_). The values are collected in Table S4 of the Supporting Information, along with the differences between them (Δ*r*_BP_ = *r*_BP_ – *R*_SL_). The averaged values of Δ*r*_BP_ were 0.2040, 0.4006, 0.0588, and 0.1451 Å for H_bay_-∗-H_bay_, C_bay_-∗-H_bay_, C_bay_-∗-C_bay_, and C_cape_-∗-C_cape_, respectively. As a result, Δ*r*_BP_ for H_bay_-∗-H_bay_ and C_bay_-∗-H_bay_ were larger than 0.20 Å, while those for C_bay_-∗-C_bay_ and C_cape_-∗-C_cape_ were less than 0.15 Å. Therefore, the BPs corresponding to C_bay_-∗-C_bay_ and C_cape_-∗-C_cape_ can be roughly approximated as straight lines since the Δ*r*_BP_ values are less than 0.20 Å (see also Figure S4 of the Supporting Information).

**Figure 3:**
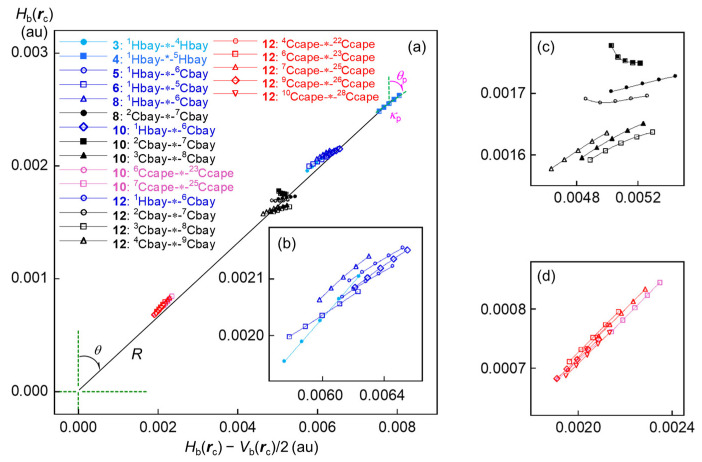
Plots of *H*_b_(***r***_c_) versus *H*_b_(***r***_c_) − *V*_b_(***r***_c_)/2 for H-∗-H, C-∗-H, and C-∗-C, exemplified by those in **3**–**6**, **8**, **10**, and **12**. (**a**) Whole picture; (**b**) Magnified picture of the C-∗-H area; (**c**) Magnified picture of the C-∗-C bay area; (**d**) Magnified picture of the C-∗-C cape area. The definitions of (*R*, *θ*) and (*θ*_p_, *κ*_p_) are illustrated, exemplified by H-∗-H in **4**.

The QTAIM functions were calculated at BCPs on X-∗-Y of **3**–**12** with M06-2X/6-311+G(3d,p). Table 1 collects the *ρ*_b_(***r***_c_), *H*_b_(***r***_c_) − *V*_b_(***r***_c_)/2, and *H*_b_(***r***_c_) values for one of the X-∗-Y if it is doubly degenerated due to the *C*_2_ symmetry of the optimized structures. Figure 3 shows the plots of *H*_b_(***r***_c_) versus *H*_b_(***r***_c_) – *V*_b_(***r***_c_)/2 for each X-∗-Y, exemplified by **3**–**6**, **8**, **10**, and **12**, where H-∗-H was detected in **3** and **4** and C-∗-H and C-∗-C were detected in **8**, **10**, and **12**. (See Figure S5 of the Supporting Information for **7**, **9**, and **11**).

The plots were analyzed according to Equations (SA3)–(SA6) of the Supporting Information. Table 1 also collects the QTAIM-DFA parameters of (*R*, *θ*) and (*θ*_p_, *κ*_p_) for each X-∗-Y of **3**–**12**, along with the *C_ii_* values corresponding to the interactions in question. The (*θ*_p_, *κ*_p_) values, evaluated with CIV, should be denoted by (*θ*_p:CIV_, *κ*_p:CIV_), respectively. However, (*θ*_p_, *κ*_p_) will be used in place of (*θ*_p:CIV_, *κ*_p:CIV_) to simplify the notation. The QTAIM functions and QTAIM-DFA parameters calculated with M06-2X/6-311+G(2d,p) and LC-ωPBE/6-311+G(2d,p) are collected in Tables S5 and S6 of the Supporting Information respectively.

### 3.3. Nature of Each X-∗-Y in ***3**–**12***

The criteria shown in Scheme SA3 and Table SA1 of the Supporting Information indicate that the interactions in the range of 45° < *θ* < 90° should be classified as pure closed-shell (*p*-CS) interactions. In the *p*-CS region of 45° < *θ* < 90°, the character of the interactions will be the vdW type for 45° < *θ*_p_ < 90° (45° < *θ* < 75°), whereas the character of the interactions will be the typical hydrogen bond type (*t*-HB) with no covalency (*t*-HB_nc_) for 90° < *θ*_p_ < 125° (75° < *θ* < 90°), where *θ* = 75° and *θ*_p_ = 125° are tentatively given for *θ*_p_ = 90° and *θ* = 90°, respectively.

The C atoms in helicenes **3**–**12** were subdivided into C_bay_ and C_cape_ based on the positions of the atoms in the species, as were the H atoms into H_bay_ and H_cape_. The bay and cape areas (positions) in the species are illustrated in Scheme 1. While both the C_bay_ and C_cape_ atoms of **3**–**12** participate in the interactions as BPs, only H_bay_ atoms participate as BPs. The *θ* and *θ*_p_ values for H-∗-H, C-∗-H, and C-∗-C of **3**–**12**, collected in Table 2, are all less than 90°, except for *θ*_p_ of ^2^C_bay_-∗-^7^C_bay_ in **10**, where (*θ*, *θ*_p_) = (70.5°, 94.2°). The ^2^C_bay_-∗-^7^C_bay_ interaction in **10** is denoted by **10** (^2^C_bay_-∗-^7^C_bay_) (see also Table 1). Therefore, the H-∗-H, C-∗-H, and C-∗-C interactions of **3**–**12** are all classified as *p*-CS interactions and characterized to have a vdW nature, which is denoted by *p*-CS/vdW, except for **10** (^2^C_bay_-∗-^7^C_bay_), which is predicted to have a *p*-CS/*t*-HB_nc_ nature.

Next, the interactions were individually examined. The (*θ*, *θ*_p_) values are (71.3°, 72.8°) and (71.8°, 74.5°) for **3** (^1^H_bay_-∗-^4^H_bay_) and **4** (^1^H_bay_-∗-^5^H_bay_), respectively. The *θ* values for **3** (^1^H_bay_-∗-^4^H_bay_) and **4** (^1^H_bay_-∗-^5^H_bay_) are larger than those of A-∗-HF (A = He, Ne, and Ar: (*θ*, *θ*_p_) = (59.9–70.9°, 64.0–88.0°)), whereas the *θ*_p_ values are larger than those of A-∗-HF (A = He and Ar). The interaction in **4** is estimated to be slightly stronger than that in **3**, although the real image of **3** (^1^H_bay_-∗-^4^H_bay_) has been much debated [69,70,71]. The detection of BPs with BCPs for **3** (^1^H_bay_-∗-^4^H_bay_) would not show enough strength for the interaction. It could be the mathematical results of the treatment. Nevertheless, **3** (^1^H_bay_-∗-^4^H_bay_) and **4** (^1^H_bay_-∗-^5^H_bay_) are discussed as very weak interactions in this work because the (*θ*, *θ*_p_) values are larger than those of A-∗-HF (A = He, Ne, and Ar). Double H_bay_-∗-C_bay_ interactions are detected for each of **5**–**12**, with (*θ*, *θ*_p_) values of (70.7–71.6°, 76.7–80.8°). The (*θ*, *θ*_p_) values are very close to those of A-∗-HF (A = He, Ne, and Ar). The BP (H_bay_-∗-C_bay_) in **6** and **9** connect the H_bay_ and C_bay_ atoms. However, they are not located at the nearest positions, as shown in Table 1 and Figure S3 of the Supporting Information. Therefore, the BP (H_bay_-∗-C_bay_) in **6** and **9** should be analyzed carefully.

One, one, four, four, seven, and eight different types of C-∗-C interactions are detected for **7**–**12**, respectively. The (*θ*, *θ*_p_) values for C-∗-C in **7**–**12** are (70.0–72.9°, 66.3–94.2°). It appears better to separately examine the values for two groups of C_bay_-∗-C_bay_ and C_cape_-∗-C_cape_. While the (*θ*, *θ*_p_) values of C_bay_-∗-C_bay_ in **7**–**12** are (71.5–72.9°, 79.5–87.8°), the values are (70.0–70.7°, 66.3–69.7°) for C_cape_-∗-C_cape_. The *θ* values for C_cape_-∗-C_cape_ are slightly smaller than those of C_bay_-∗-C_bay_ (by 0.5–2.2°), but the *θ*_p_ values for C_cape_-∗-C_cape_ are much smaller than those of C_bay_-∗-C_bay_ (by 13.2–24.5°). In this case, *θ*_p_ < *θ* for C_cape_-∗-C_cape_, whereas *θ*_p_ > *θ* for C_bay_-∗-C_bay_. Interactions with *θ*_p_ > *θ* are usually observed, but interactions with *θ*_p_ < *θ* are rare.

**Figure 4:**
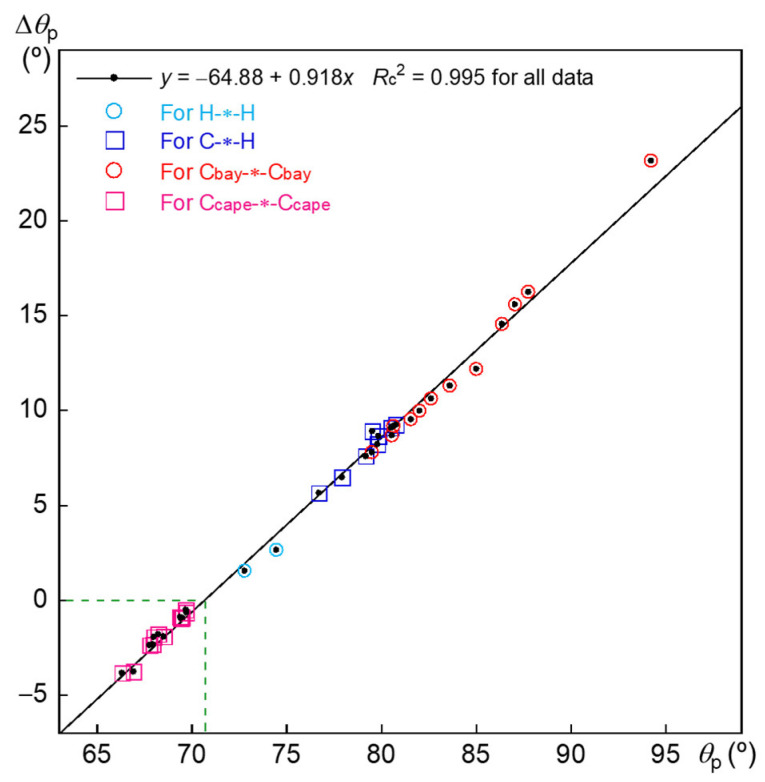
Plot of Δ*θ*_p_ versus *θ*_p_ for H-∗-H, C-∗-H, and C-∗-C in **3**–**12**, evaluated with M06-2X/6-311+G(3d,p), where Δ*θ*_p_ = (*θ*_p_ – *θ*).

Interactions with *θ* > *θ*_p_ occur under some specific conditions. To examine the behaviour of *θ* and *θ*_p_ in **7**–**12**, the Δ*θ*_p_ (=*θ*_p_ – *θ*) values are plotted versus *θ*_p_ for C-∗-C, H-∗-H and C-∗-H in **3**–**12**. Figure 4 shows this plot. The plot showed a very good correlation for all data (*y* = 0.918*x* – 64.88: *R*_c_^2^ = 0.995). (A substantial correlation was not found in the plot of Δ*θ*_p_ versus *θ* due to the very small range of *θ*). The two areas for C-∗-C interactions with Δ*θ*_p_ > 0 and Δ*θ*_p_ < 0 are clearly illustrated by the green dotted lines in Figure 4. The Δ*θ*_p_ values for the interactions are positive if the *θ*_p_ values are larger than 70.7°, whereas Δ*θ*_p_ < 0 if *θ*_p_ < 70.7°. Figure 4 clearly shows that C_bay_-∗-C_bay_ and C_cape_-∗-C_cape_ in **9**–**12** belong to the areas where Δ*θ*_p_ > 0 and Δ*θ*_p_ < 0, respectively.

It seems difficult to clearly explain the results shown in Figure 4; however, our explanation is as follows: The static nature of the interactions described by *θ* should be a measure of the strength of the interactions. If so, the steric compression on C_cape_-∗-C_cape_ in **9**–**12** appears to be similar to that on C_bay_-∗-C_bay_ in fully optimized structures. Namely, the C_cape_-∗-C_cape_ and C_bay_-∗-C_bay_ interactions in the fully optimized structures of **9**–**12** would be affected similarly to steric compression, according to the *θ* values. On the other hand, the dynamic nature of the interactions is defined by *θ*_p_ based on the behaviour of the interactions in the perturbed structures. The C_bay_-∗-C_bay_ interactions in the perturbed structures will be affected by steric compression, similar to the usual cases of interactions, whereas the C_cape_-∗-C_cape_ interactions will be inversely affected compared with the usual cases when measured by the *θ*_p_ values at the BCPs of the interactions.

### 3.4. Nature of Each X-∗-Y in ***6***:***6*** and ***7***:***7***

**Figure 5:**
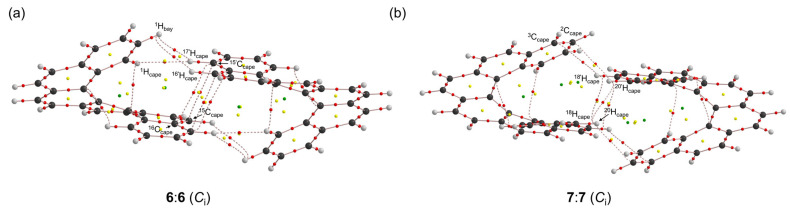
Molecular graphs for helicene dimers, **6**:**6** (**a**) and **7**:**7** (**b**), calculated with M06-2X/6-311+G(3d,p), where BPs with BCPs corresponding to intra- and intermolecular noncovalent interactions are detected. The BCPs are denoted by red dots, RCPs (ring critical points) by yellow dots, CCPs (cage critical points) by green dots, and BPs by pink lines. The carbon atoms are in black and the hydrogen atoms are in grey.

What is the behaviour of the interactions when the helicenes form concave-type dimers? The behaviour was elucidated, exemplified by **6**:**6** (*C*_i_) and **7**:**7** (*C*_i_) with M06-2X/6-311+G(3d,p). Figure 5 shows molecular graphs of **6**:**6** and **7**:**7**. Five and four independent BPs with BCPs were detected in **6**:**6** and **7**:**7**, respectively, between the components of H-∗-H and C-∗-H, as well as two independent BPs with BCPs for the intramolecular C-∗-H interactions in each component of **6**:**6** and **7**:**7**. The behaviour of the interactions was also investigated for **7**:**7** (*C*_i_), **8**:**8** (*C*_i_), and/or **10**:**10** (*C*_i_) with M06-2X/6-311+G(2d,p) and LC-ωPBE/6-311+G(2d,p). The results are collected in Tables S7 and S8 of the Supporting Information. The QTAIM functions were similarly calculated for the intermolecular interactions at the BCPs on the BPs of **6**:**6** and **7**:**7** with M06-2X/6-311+G(3d,p).

**Figure 6:**
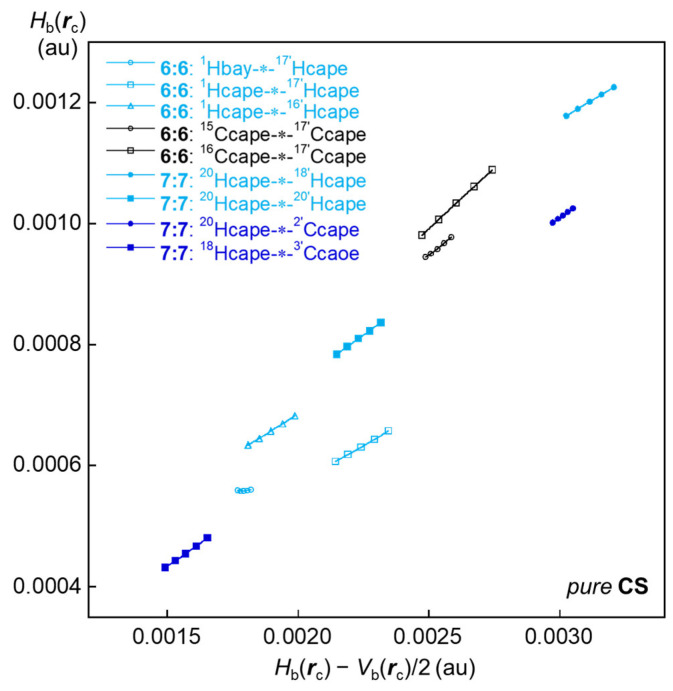
Plots of *H*_b_(***r***_c_) versus *H*_b_(***r***_c_) – *V*_b_(***r***_c_)/2 for H-∗-H, C-∗-H, and C-∗-C at **6**:**6** (*C*_i_) and **7**:**7** (*C*_i_), calculated with M06-2X/6-311+G(3d,p).

Table 2 collects the *ρ*_b_(***r***_c_), *H*_b_(***r***_c_) − *V*_b_(***r***_c_)/2, and *H*_b_(***r***_c_) values for one of the doubly degenerate interactions due to the *C*_i_ symmetry of the optimized structures. Figure 6 shows the plots of *H*_b_(***r***_c_) versus *H*_b_(***r***_c_) − *V*_b_(***r***_c_)/2 for each interaction between the components at **6**:**6** and **7**:**7**. (The plots for **8**:**8** and **10**:**10** are shown in Figure S7 of the Supporting Information, and the data are collected in Table S8 of the Supporting Information).

The plots were analyzed similarly to the case of **3**–**12**. Table 2 also collects the QTAIM-DFA parameters of (*R*, *θ*) and (*θ*_p_, *κ*_p_) for the intermolecular interactions in question at **6**:**6** and **7**:**7**, together with the *C_ii_* values corresponding to the interactions in question. The (*θ*, *θ*_p_) values for the three H-∗-H and two C-∗-C intermolecular independent interactions of **6**:**6** are (70.9–74.3°, 74.9–88.1°) and (68.3–69.2°, 68.0–70.8°), respectively. The (*θ*, *θ*_p_) values for the couple of H-∗-H and two C-∗-H intermolecular independent interactions at **7**:**7** are (68.9–70.0°, 72.7–75.5°) and (71.4–73.8°, 73.2–73.3°), respectively. The *θ* and *θ*_p_ values for the intermolecular H-∗-H, C-∗-H, and C-∗-C interactions at **6**:**6** and **7**:**7** are all less than 90°; therefore, the interactions are all predicted to have a *p*-CS/vdW nature (see Table 2). The interactions appear to be very weak, based on the (*θ*, *θ*_p_) values. However, (*θ*, *θ*_p_) = (72.6°, 88.1°) for **6**:**6** (^1^H_bay_-∗-^17’^H_cape_), of which nature seems close to *p*-CS/*t*-HB_nc_.

In the case of intramolecular interactions, ^1^H_bay_-∗-^5^C_bay_ and ^3^C_bay_-∗-^7^H_bay_ were detected at **6**:**6**. The former was also observed in **6**, whereas the latter was newly detected in **6**:**6**. The new appearance of **6** (^3^C_bay_-∗-^7^H_bay_) may be due to a structural change at **6**:**6** relative to **6**. Similarly, ^1^H_bay_-∗-^6^C_bay_ and ^3^C_bay_-∗-^8^H_bay_ were detected at **7**:**7**. The former was observed in **7**, while ^3^C_bay_-∗-^8^H_bay_ in **7**:**7** appeared in place of ^2^C_bay_-∗-^7^C_bay_ in **7**. The change in the optimized structures between **7** and **7**:**7** would again be responsible for the results. However, clarifying the reason for the appearance/disappearance of BPs is very complex and difficult in helicenes, and it is beyond the scope of this work.

Highly theoretical treatment must be necessary to clarify the reason for the appearance and disappearance of BPs/BCPs. Pendás and coworkers discussed BPs as privileged exchange channels, using the interacting quantum atom (IQA) framework [72]. They have investigated how BPs between an atom A and atoms B in its environment appear to be determined by competition among the A–B exchange correlation energies that always contribute to stabilize the A–B interactions. And they have predicted that a BP is found between two atoms by examining a number of archetypal simple systems: (1) there is no other competing atom in its vicinity, so there must be a direct exchange route between them or (2) its **V**_xc_ term is the largest among several possibilities, where **V**_xc_ stands for a quantum-mechanical correction coming from the exchange correlation second-order density [72]. It has also indicated that interaction energies between both atoms cannot be universally used to predict the existence of a BP between them [73]. Moreover, they are not correlated to distances or to the density values at BCPs. On the contrary, the exchange contribution is shown to be an appropriate descriptor [73]. Similarly, theoretical treatments are applied to various interactions, employing QTAIM-defined an atomic interaction line (AIL: Presence or absence), IQA-defined interaction energy and its components, NCI (non-covalent interactions)-defined isosurfaces, and deformation density [74]. The reason for the appearance and disappearance of BPs/BCPs in the helicenes would be rationalized by applying above theory [27].

The (*θ*, *θ*_p_) values for the intramolecular interactions at **6:6** and **7**:**7** are (70.8–71.9°, 78.3–80.0°). As a result, the interactions are all predicted to have a *p*-CS/vdW nature (see Table 2). The predicted natures of the interactions in **6**:**6** and **7**:**7** appear to be similar to those in **6** and **7**, perhaps due to the very weak nature of both dimers and monomers.

## 4. Basis Set and Level Dependence of the Predicted Natures

**Figure 7:**
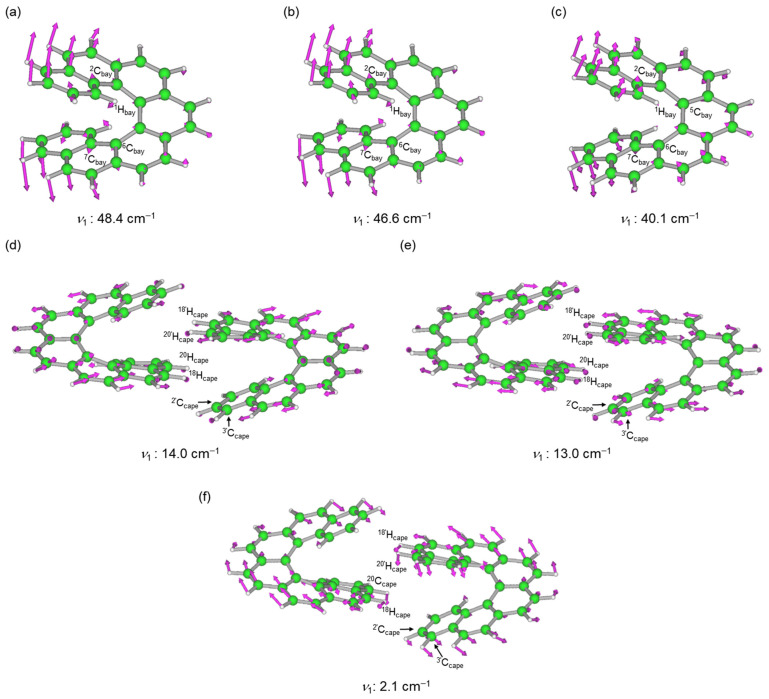
The internal vibrational motions of *ν*_1_ for **7** (*C*_2_) and **7**:**7** (*C*_i_). (**a**) For **7** (*C*_2_) calculated with M06-2X/6-311+G(3d,p); (**b**) for **7** (*C*_2_) calculated with M06-2X/6-311+G(2d,p); (**c**) for **7** (*C*_2_) calculated with LC-ωPBE/6-311+G(2d,p); (**d**) for **7**:**7** (*C*_i_) calculated with M06-2X/6-311+G(3d,p); (**e**) for **7**:**7** (*C*_i_) calculated with M06-2X/6-311+G(2d,p); (**f**) for **7**:**7** (*C*_i_) calculated with LC-ωPBE/6-311+G(2d,p).

The basis set and level dependence of the predicted natures was investigated, exemplified by **7** and **7**:**7**, to attempt to determine the reason why the optimized structures can easily change. Table 3 shows the QTAIM-DFA parameters of (*R*, *θ*) and (*θ*_p_, *κ*_p_) and the *C_ii_* values, calculated with M06-2X/6-311+G(3d,p), M06-2X/6-311+G(2d,p), and LC-ωPBE/6-311+G(2d,p). Table 3 includes the distances in question as well as some internal vibration(s) *ν_n_* corresponding to the interactions in question, which are closely related to (*θ*_p_, *κ*_p_). Figure 7 shows the motions of the internal vibrations for *ν*_1_ of **7** and **7**:**7** calculated with M06-2X/6-311+G(3d,p), M06-2X/6-311+G(2d,p), and LC-ωPBE/6-311+G(3d,p). (Other motions are shown in Figure S9 of the Supporting Information).

The calculated *r*(^1^H_bay_···^6^C_bay_) and *r*(^2^C_bay_···^7^C_bay_) distances were 2.590 Å and 2.975 Å, respectively, for **7**, when calculated with M06-2X/6-311+(3d,p), while the values were 2.590 Å and 3.003 Å, respectively, when calculated with M06-2X/6-311+(2d,p). The differences are less than 0.001 Å for *r*(^1^H_bay_···^6^C_bay_) and 0.028 Å for *r*(^2^C_bay_···^7^C_bay_). The results show that the structure of **7** optimized with M06-2X/6-311+(2d,p) appears to be nearly identical to that optimized with M06-2X/6-311+(3d,p). On the other hand, the *r*(^1^H_bay_···^6^C_bay_) and *r*(^2^C_bay_···^7^C_bay_) of **7** were 2.561 Å and 3.227 Å, respectively, if calculated with LC-ωPBE/6-311+(2d,p). The difference was −0.029 Å for the former but 0.224 Å for the latter relative to the corresponding values calculated with M06-2X/6-311+(2d,p). The structure of **7** optimized with LC-ωPBE/6-311+(2d,p) appears to be (very) different from that optimized with M06-2X/6-311+(2d,p), especially around *r*(^2^C_bay_···^7^C_bay_). In the case of ^1^H_bay_···^5^C_bay_, the distance was optimized to be 2.468 Å with LC-ωPBE/6-311+(2d,p), which is shorter than the *r*(^1^H_bay_···^6^C_bay_) distance optimized with M06-2X/6-311+(2d,p) (2.590 Å) by 0.122 Å. BPs (with BCPs) were detected for ^1^H_bay_-∗-^6^C_bay_ and ^2^C_bay_-∗-^7^C_bay_ in **7** if calculated with M06-2X/6-311+(3d,p) and M06-2X/6-311+(2d,p), while a BP was detected for ^1^H_bay_-∗-^5^C_bay_ if calculated with LC-ωPBE/6-311+(2d,p). The ^2^C_bay_-∗-^7^C_bay_ and ^1^H_bay_-∗-^5^C_bay_ distances in **7**, optimized with LC-ωPBE/6-311+(2d,p), were (much) longer and shorter than those optimized with M06-2X/6-311+(2d,p), respectively.

The differences in the optimized distances appear to be the main factor for the appearance/disappearance of the BPs, although predicting the appearance/disappearance of the BPs is very difficult and complex. Despite such different results, the motion of *ν*_1_ appears to be very similar when calculated at both the M06-2X and LC-ωPBE levels, indicating that *ν*_1_ is a good measure for imaging the dynamic nature of the π···π interactions in **7** among the internal vibrations. Small differences in the dynamic nature of the interactions predicted at both the M06-2X and LC-ωPBE levels result from the (very) similar motion of *ν*_1_. The magnitudes of the displacements in the cape area seem (much) larger than those in the bay area in *ν*_1_. This will be instructive if the relationship is clarified for that between the magnitudes of the displacements and the Δ*θ*_p_ values. This issue will be investigated in a future work. The very low energy of *ν*_1_ in **7** suggests the basis set and level dependence can easily change the optimized structure.

In the case of **7**:**7**, the *r*(^20^H_cape_···^18^^’^H_cape_), *r*(^20^H_cape_···^20^^’^H_bay_), *r*(^20^H_cape_···^2^^’^C_cape_), and *r*(^8^H_cape_···^3^^’^C_cape_) distances were 2.543 Å, 2.716 Å, 2.677 Å, and 2.964 Å, respectively, when calculated with M06-2X/6-311+(3d,p), while the values were 2.564 Å, 2.729 Å, 2.688 Å, and 2.986 Å, respectively, when calculated with M06-2X/6-311+(2d,p). The differences are 0.011–0.022 Å, which are less than approximately 0.02 Å. The results show that the optimized structures of **7**:**7** are very similar with both M06-2X/6-311+(3d,p) and M06-2X/6-311+(2d,p). On the other hand, the distances are optimized to be 2.947 Å, 3.015 Å, 3.066 Å, and 3.659 Å with LC-ωPBE/6-311+(2d,p). The differences with the corresponding values of M06-2X/6-311+(2d,p) are 0.286–0.674 Å. Namely, the structure of **7**:**7** optimized with LC-ωPBE/6-311+(2d,p) appears to be very different from that optimized with M06-2X/6-311+(2d,p), similar to the case of **7**.

The ^20^C_cape_-∗-^18’^H_cape_ distance was optimized to be 3.236 Å, which is longer than *r*(^20^H_cape_···^18’^H_cape_) (2.947 Å) by 0.288 Å with LC-ωPBE/6-311+(2d,p). However, a BP was detected for ^20^C_cape_-∗-^18’^H_cape_ but not for ^20^H_cape_-∗-^18’^H_cape_. The difference in the atomic size between C and H, such as the van der Waals radii, may be responsible for the predicted results, in this case. The ^20^C_cape_-∗-^18^^’^H_cape_, ^20^H_cape_-∗-^20^^’^H_bay_, ^20^H_cape_-∗-^2^^’^C_cape_, and ^8^H_cape_-∗-^3^^’^C_cape_ distances at **7**:**7**, optimized with LC-*ω*PBE/6-311+(2d,p), were much longer than the corresponding distances, optimized with M06-2X/6-311+(2d,p). BPs were detected for ^20^H_cape_-∗-^18^^’^H_cape_, ^20^H_cape_-∗-^20^^’^H_bay_, ^20^H_cape_-∗-^2^^’^C_cape_, and ^18^H_cape_-∗-^3^^’^C_cape_ when calculated with M06-2X/6-311+(2d,p), while they were detected for ^20^C_cape_-∗-^18^^’^H_cape_, ^20^H_cape_-∗-^20^^’^H_bay_, ^20^H_cape_-∗-^2^^’^C_cape_, and ^8^H_cape_-∗-^3^^’^C_cape_ when calculated with LC-*ω*PBE/6-311+(2d,p). The differences in the optimized distances appear to be the main factor for the appearance/disappearance of the BPs, similar to the case of **7**.

Table 3 contains the *ν*_1_ values for **7** and **7**:**7**, calculated with M06-2X/6-311+(3d,p), M06-2X/6-311+(2d,p), and LC-ωPBE/6-311+(2d,p). Table 3 also contains some vibrations closely related to the interactions in question (corresponding to the perturbed structures) at **7**:**7**, where most candidates were found to be less than *ν_n_* of *ν*_20_. The *ν*_1_ values for **7** were 48.4 cm^−1^, 46.6 cm^−1^, and 40.2 cm^−1^ when calculated with the three methods, respectively. The *ν*_1_ motion of **7** appears to be very similar when calculated with the three methods. In the case of **7**:**7**, the frequencies of *ν*_1_ calculated with M06-2X/6-311+G(3d,p) and M06-2X/6-311+G(2d,p) were 14.0 cm^−1^ and 13.1 cm^−1^, respectively, while the value calculated with LC-ωPBE was 2.1 cm^−1^. Very large differences are predicted for *ν*_1_ at **7**:**7** when calculated at the M06-2X and LC-*ω*PBE levels. The *ν*_1_ value with the motion should correspond to the strength of the interactions in the direction of the perturbed structures.

The (*θ*, *θ*_p_) values of **7**:**7** are (67.9–73.0°, 69.4–75.2°) and (62.9–67.2°, 67.4–73.9°) with M06-2X/6-311+(2d,p) and LC-ωPBE/6-311+(2d,p), respectively. The differences seem large relative to the case of **7**, with (*θ*, *θ*_p_) values of (70.4°, 81.6°) and (70.5°, 82.2°) when calculated with M06-2X/6-311+(2d,p) and LC-ωPBE/6-311+(2d,p), respectively, although **7** (^1^H_bay_-∗-^6^C_bay_) was detected with M06-2X/6-311+(2d,p) and **7** (^1^H_bay_-∗-^5^C_bay_) was detected with LC-ωPBE/6-311+(2d,p). The (optimized) structures of **7**:**7** would be affected more easily by surroundings containing the calculation methods than the case of **7**. The basis set and level dependence of the interactions in **7** and **7**:**7** can help us to better understand the interactions in helicenes.

The unit of *C_ii_* [Å mdyn^−1^] is the inverse of that of the force constant, which corresponds to the frequency. Therefore, the strengths of the interactions should be roughly inversely proportional to the *C_ii_* values. As shown in Table 3, the *C_ii_* values for the π···π interactions of **7** are 3.1–5.5 Å mdyn^−1^ for H_bay_-∗-C_bay_ and C_bay_-∗-C_bay_ with the three methods. The values for the π···π interactions of **7**:**7** are 10.0–27.9, 11.4–31.4, and 52.7–96.4 Å mdyn^−1^ for H_cape_-∗-H_cape_ and H_cape_-∗-C_cape_ when calculated with M06-2X/6-311+(3d,p), M06-2X/6-311+(2d,p), and LC-ωPBE/6-311+(2d,p), respectively. This consideration explains the above results.

## 5. Conclusions

It is challenging to clarify the natures of π···π interactions in helicenes since the interactions are factors that control the fine details of structures and are expected to give rise to specific functionalities for the species. The repulsive interactions between the benzene rings in helicenes must be very strong; therefore, the π···π interactions would be considered strong. The π···π interactions in the helicenes are described by the H-∗-H, C-∗-H, and C-∗-C forms with BPs and BCPs. The π···π interactions in helicenes **1**–**12**, as well as in dimers **6**:**6** and **7**:**7,** were analyzed with QTAIM-DFA after clarifying the structural features and the energy profile. *H*_b_(***r***_c_) was plotted versus *H*_b_(***r***_c_) − *V*_b_(***r***_c_)/2, and the data from the fully optimized structures and the perturbed structures around the fully optimized structures were used in QTAIM-DFA. Data from the fully optimized structures in the plots correspond to the static nature of the interactions, which are analyzed using polar coordinate (*R*, *θ*) representation. Data from both the perturbed and fully optimized structures are expressed by (*θ*_p_, *κ*_p_), where *θ*_p_ corresponds to the tangent line and *κ*_p_ is the curvature of the plot. The concept of the dynamic nature of the interactions was proposed based on (*θ*_p_, *κ*_p_).

The interactions were analyzed by dividing the C atoms of **3**–**12** into C_bay_ and C_cape_ and the H atoms into H_bay_ and H_cape_. While both C_bay_ and C_cape_ atoms of **3**–**12** take part in the interactions, only H_bay_ atoms participate as BPs. The *θ* and *θ*_p_ values for H-∗-H, C-∗-H, and C-∗-C of **3**–**12** are all less than 90°, except for **10** (^2^C_bay_-∗-^7^C_bay_), where (*θ*, *θ*_p_) = (70.5°, 94.2°). Therefore, the H-∗-H, C-∗-H, and C-∗-C interactions of **3**–**12** are all predicted to have a *p*-CS/vdW nature, except for **10** (^2^C_bay_-∗-^7^C_bay_), which is predicted to have a *p*-CS/*t*-HB_nc_ nature. While the (*θ*, *θ*_p_) values of C_bay_-∗-C_bay_ in **7**–**12** are (71.5–72.9°, 79.5–87.8°), the values are (70.0–70.7°, 66.3–69.7°) for C_cape_-∗-C_cape_. The *θ* values for C_cape_-∗-C_cape_ are slightly smaller than those of C_bay_-∗-C_bay_ (by 0.5–2.2°), but the *θ*_p_ values for C_cape_-∗-C_cape_ are much smaller than those of C_bay_-∗-C_bay_ (by 13.2–24.5°). In this case, *θ* < *θ*_p_ for C_bay_-∗-C_bay_, whereas *θ* > *θ*_p_ for C_cape_-∗-C_cape_. Interactions with *θ* < *θ*_p_ are usually observed, whereas interactions with *θ* > *θ*_p_ are rare.

The H-∗-H, C-∗-H, and C-∗-C interactions of dimers **6**:**6** and **7**:**7** were similarly analyzed. The interactions were predicted to have a *p*-CS/vdW nature, although **6**:**6** (^1^H_bay_-∗-^17’^H_cape_) has a nature close to *p*-CS/*t*-HB_nc_, since (*θ*, *θ*_p_) = (72.6°, 88.1°). The interactions at **3**–**12** and **6**:**6** and **7**:**7** were predicted to be much weaker than expected. The very low energy of *ν*_1_ of **7**:**7** supports the very weak nature predicted for interactions and the easy dependence of the levels on the nature of the interactions. The strength of the interactions can also be estimated by the *C_ii_*^−1^ values. Detecting the interactions and predicting the nature of helicenes will provide a solid basis for investigating and applying the interactions in helicenes.

## Figures and Tables

**Table 1:** QTAIM Functions and QTAIM-DFA Parameters Evaluated for the Fused Benzene-Type Helicenes of Monomers (**3**–**12**), Employing the Perturbed Structures Generated with CIV ^1–3^.

Species	*ρ*_b_(*r*_c_)	*c*∇^2^*ρ*_b_(*r*_c_) ^4^	*H*_b_(*r*_c_)	*R* ^5^	*θ* ^6^	*C_ii_* ^7^	*θ*_p_ ^8^	*κ*_p_ ^9^	Predicted Nature
X-∗-Y	(*ea*_o_^–3^)	(au)	(au)	(au)	(°)	(Å mdyn^−1^)	(°)	(au^−1^)	
3 (^1^H_bay_-∗-^4^H_bay_)	0.0130	0.0060	0.0020	0.0063	71.3	3.29	72.8	12.4	*p*-CS/vdW
4 (^1^H_bay_-∗-^5^H_bay_)	0.0165	0.0078	0.0026	0.0082	71.8	6.67	74.5	11.4	*p*-CS/vdW
5 (^1^H_bay_-∗-^6^C_bay_)	0.0131	0.0063	0.0021	0.0066	71.6	6.12	80.8	136	*p*-CS/vdW
6 (^1^H_bay_-∗-^5^C_bay_)	0.0131	0.0060	0.0020	0.0063	71.3	3.82	79.9	31.8	*p*-CS/vdW
7 (^1^H_bay_-∗-^6^C_bay_)	0.0135	0.0063	0.0021	0.0067	71.5	5.47	77.9	188	*p*-CS/vdW
7 (^2^C_bay_-∗-^7^C_bay_)	0.0114	0.0051	0.0017	0.0054	71.9	3.33	80.6	128	*p*-CS/vdW
8 (^1^H_bay_-∗-^6^C_bay_)	0.0130	0.0061	0.0021	0.0065	71.1	5.51	76.7	189	*p*-CS/vdW
8 (^2^C_bay_-∗-^7^C_bay_)	0.0117	0.0052	0.0017	0.0055	71.8	2.01	86.4	28.5	*p*-CS/vdW
9 (^1^H_bay_-∗-^5^C_bay_)	0.0134	0.0062	0.0022	0.0066	70.7	3.40	79.5	627	*p*-CS/vdW
9 (^2^C_bay_-∗-^7^C_bay_)	0.0113	0.0050	0.0016	0.0053	72.1	1.83	81.6	119	*p*-CS/vdW
9 (^3^C_bay_-∗-^8^C_bay_)	0.0122	0.0053	0.0016	0.0056	72.9	1.87	85.0	196	*p*-CS/vdW
9 (^4^C_cape_-∗-^22^C_cape_) ^10^	0.0055	0.0020	0.0007	0.0022	70.7	5.25	66.9	135	*p*-CS/vdW
9 (^6^C_cape_-∗-^23^C_cape_)	0.0061	0.0021	0.0007	0.0022	70.4	8.51	69.4	37.7	*p*-CS/vdW
10 (^1^H_bay_-∗-^6^C_bay_)	0.0137	0.0064	0.0021	0.0067	71.6	5.74	79.2	123	*p*-CS/vdW
10 (^2^C_bay_-∗-^7^C_bay_) ^11^	0.0113	0.0050	0.0018	0.0053	70.5	1.86	94.2	2890	*p*-CS/*t*-HB_nc_
10 (^3^C_bay_-∗-^8^C_bay_)	0.0114	0.0050	0.0016	0.0053	72.1	1.78	82.0	182	*p*-CS/vdW
10 (^6^C_cape_-∗-^23^C_cape_)	0.0059	0.0020	0.0007	0.0021	70.4	9.02	69.7	7.0	*p*-CS/vdW
10 (^7^C_cape_-∗-^25^C_cape_)	0.0061	0.0022	0.0008	0.0024	70.3	3.42	68.0	10.3	*p*-CS/vdW
11 (^1^H_bay_-∗-^6^C_bay_)	0.0136	0.0063	0.0021	0.0067	71.6	5.41	79.8	113	*p*-CS/vdW
11 (^2^C_bay_-∗-^7^C_bay_)	0.0116	0.0051	0.0017	0.0054	71.5	1.84	87.1	142	*p*-CS/vdW
11 (^3^C_bay_-∗-^8^C_bay_)	0.0115	0.0050	0.0016	0.0053	72.0	1.91	82.6	166	*p*-CS/vdW
11 (^4^C_bay_-∗-^9^C_bay_)	0.0111	0.0049	0.0016	0.0052	71.7	1.69	79.5	155	*p*-CS/vdW
11 (^4^C_cape_-∗-^22^C_cape_)	0.0053	0.0019	0.0007	0.0020	70.1	6.81	68.3	13.5	*p*-CS/vdW
11 (^6^C_cape_-∗-^23^C_cape_)	0.0059	0.0020	0.0007	0.0021	70.2	10.21	69.7	7.5	*p*-CS/vdW
11 (^7^C_cape_-∗-^25^C_cape_)	0.0059	0.0022	0.0008	0.0023	70.2	3.58	67.8	7.2	*p*-CS/vdW
11 (^9^C_cape_-∗-^26^C_cape_)	0.0062	0.0021	0.0007	0.0022	70.4	5.80	69.5	64.7	*p*-CS/vdW
12 (^1^H_bay_-∗-^6^C_bay_)	0.0136	0.0063	0.0021	0.0067	71.5	4.84	80.5	103	*p*-CS/vdW
12 (^2^C_bay_-∗-^7^C_bay_)	0.0115	0.0051	0.0017	0.0053	71.5	1.77	87.8	349	*p*-CS/vdW
12 (^3^C_bay_-∗-^8^C_bay_)	0.0117	0.0051	0.0016	0.0053	72.3	1.74	83.6	241	*p*-CS/vdW
12 (^4^C_bay_-∗-^9^C_bay_)	0.0110	0.0048	0.0016	0.0051	71.5	1.72	80.7	87.8	*p*-CS/vdW
12 (^4^C_cape_-∗-^22^C_cape_)	0.0055	0.0020	0.0007	0.0022	70.2	4.80	66.3	697	*p*-CS/vdW
12 (^6^C_cape_-∗-^23^C_cape_)	0.0060	0.0021	0.0008	0.0022	70.0	6.78	68.0	8.6	*p*-CS/vdW
12 (^7^C_cape_-∗-^25^C_cape_)	0.0059	0.0022	0.0008	0.0023	70.0	3.41	68.2	3.3	*p*-CS/vdW
12 (^9^C_cape_-∗-^26^C_cape_)	0.0059	0.0020	0.0007	0.0021	70.3	6.95	69.4	24.7	*p*-CS/vdW
12 (^10^C_cape_-∗-^28^C_cape_)	0.0056	0.0020	0.0007	0.0022	70.5	4.09	68.6	65.4	*p*-CS/vdW

^1^ Calculated with M06-2X/6-311+G(3d,p). ^2^ Data are given at the BCPs. ^3^ All interactions are predicted to have the *p*-CS/vdW nature, except for **10** (^2^C_bay_-∗-^7^C_bay_), which has the *p*-CS/*t*-HB_nc_ nature. ^4^ *c*∇^2^*ρ*_b_(***r***_c_) = *H*_b_(***r***_c_) − *V*_b_(***r***_c_)/2, where *c* = *ℏ*^2^/8*m*. ^5^ *R* = (*x*^2^ + *y*^2^)^1/2^, where (*x*, *y*) = (*H*_b_(***r***_c_) − *V*_b_(***r***_c_)/2, *H*_b_(***r***_c_)). ^6^ *θ* = 90° − tan^−1^ (*y*/*x*). ^7^ *C_ij_* = *∂*^2^*E*/*∂f_i_∂f_j_*, where *i* and *j* refer to internal coordinates, and the external force components acting on the system *f_i_* and *f_j_* correspond to *i* and *j*, respectively. ^8^ *θ*_p_ = 90° − tan^−1^ (d*y*/d*x*). ^9^ *κ*_p_ = ?d^2^*y*/d*x*^2^?/[1 + (d*y*/d*x*)^2^]^3/2^. ^10^ Data from *w* = ±0.0125, ±0.025, and 0 were used for the plot since BCPs were not detected at *w* = ±0.05. ^11^ Data from *w* = −0.05, −0.0375, −0.025, −0.0125, and 0 were used for the plot since BCPs were not detected when *w* > 0.

**Table 2:** QTAIM Functions and QTAIM-DFA Parameters Evaluated for the Fused Benzene-Type Helicenes of Concave-Type Dimers (**6**:**6** and **7**:**7**), Employing the Perturbed Structures Generated with CIV ^1–3^.

Species	*ρ*_b_(*r*_c_)	*c*∇^2^*ρ*_b_(*r*_c_)	*H*_b_(*r*_c_)	*R*	*θ*	*C_ii_*	*θ*_p_	*κ*_p_	Predicted Nature
X-∗-Y	(*ea*_o_^–3^)	(au)	(au)	(au)	(°)	(Å mdyn^−1^)	(°)	(au^−1^)	
**6**:**6** (^1^H_bay_-∗-^17’^H_cape_) ^4^	0.0045	0.0018	0.0006	0.0019	72.6	25.02	88.1	5163	*p*-CS/vdW
**6**:**6** (^1^H_cape_-∗-^17’^H_cape_)	0.0061	0.0022	0.0006	0.0023	74.3	35.63	76.2	184.2	*p*-CS/vdW
**6**:**6** (^1^H_cape_-∗-^16’^H_cape_)	0.0051	0.0019	0.0007	0.0020	70.9	42.01	74.9	69.9	*p*-CS/vdW
**6**:**6** (^15^C_cape_-∗-^17’^C_cape_) ^5^	0.0065	0.0025	0.0009	0.0027	69.2	12.64	70.8	1066	*p*-CS/vdW
**6**:**6** (^16^C_cape_-∗-^17’^C_cape_)	0.0066	0.0026	0.0010	0.0028	68.3	8.63	68.0	53.5	*p*-CS/vdW
**6**:**6** (^1^H_bay_-∗-^5^C_bay_)	0.0128	0.0057	0.0019	0.0060	71.9	3.817	78.3	32.3	*p*-CS/vdW
**6**:**6** (^3^C_bay_-∗-^7^H_bay_)	0.0134	0.0061	0.0020	0.0064	71.7	3.414	79.3	29.5	*p*-CS/vdW
**7**:**7** (^20^H_cape_-∗-^18’^H_cape_)	0.0073	0.0031	0.0012	0.0033	68.9	14.61	75.5	24.6	*p*-CS/vdW
**7**:**7** (^20^H_cape_-∗-^20’^H_cape_)	0.0054	0.0022	0.0008	0.0024	70.0	27.90	72.7	36.5	*p*-CS/vdW
**7**:**7** (^20^H_cape_-∗-^2’^C_cape_)	0.0079	0.0030	0.0010	0.0032	71.4	10.01	73.3	125.7	*p*-CS/vdW
**7**:**7** (^18^H_cape_-∗-^3’^C_cape_)	0.0050	0.0016	0.0005	0.0016	73.8	17.36	73.2	181.8	*p*-CS/vdW
**7**:**7** (^1^H_bay_-∗-^6^C_bay_)	0.0132	0.0062	0.0021	0.0065	70.8	5.557	80.0	93.5	*p*-CS/vdW
**7**:**7** (^3^C_bay_-∗-^8^H_bay_)	0.0138	0.0065	0.0021	0.0068	71.9	5.024	78.8	154.1	*p*-CS/vdW

^1^ Calculated with M06-2X/6-311+G(3d,p). ^2^ Data are given at the BCPs. ^3^ See footnotes of Table 1 for the QTAIM-DFA parameters and *C_ii_*. ^4^ Data from *w* = −0.0375, −0.025, −0.0125, 0, and 0.0125 were used for the plot, since BCPs for **6**:**6** (^1^H_bay_-∗-^17’^H_cape_) were not detected when *w* > 0.0125. ^5^ Data from *w* = −0.05, −0.0375, −0.025, −0.0125, and 0 were used for the plot, since BCPs for **6**:**6** (^15^C_cape_-∗-^17’^C_cape_) were not detected when *w* > 0.

**Table 3:** QTAIM Functions and QTAIM-DFA Parameters Evaluated for the Fused Benzene-Type Helicene (**7**) and the Concave-Type Dimer (**7**:**7**), Employing the Perturbed Structures Generated with CIV, together with the X-∗-Y Distances and the Corresponding Internal Vibrations, with the Frequencies Closely Related to the Interactions in Question ^1,2^.

Species	*r*(X···Y)	*R*	*θ*	*C_ii_*	*θ*_p_	*κ*_p_	Predicted Nature
X-∗-Y	(Å)	(au)	(°)	(Å mdyn^−1^)	(°)	(au^−1^)	
**7** (M06-2X/6-311+G(3d,p): *ν*_1_ = 43.4 cm^−1^)							
^1^H_bay_-∗-^6^C_bay_	2.5902	0.0067	71.5	5.47	77.9	187.5	*p*-CS/vdW
^2^C_bay_-∗-^7^C_bay_	2.9751	0.0054	71.9	3.33	80.6	128.2	*p*-CS/vdW
**7** (M06-2X/6-311+G(2d,p): *ν*_1_ = 46.6 cm^−1^)							
^1^H_bay_-∗-^6^C_bay_	2.5896	0.0067	70.4	5.49	81.6	39.9	*p*-CS/vdW
^2^C_bay_-∗-^7^C_bay_	3.0025	0.0054	69.9	3.06	78.7	120.2	*p*-CS/vdW
**7** (LC-ωPBE/6-311+G(2d,p): *ν*_1_ = 40.1 cm^−1^) ^3,4^							
^1^H_bay_-∗-^5^C_bay_	2.4681	0.0066	70.5	3.72	82.2	46.4	*p*-CS/vdW
**7**:**7** (M06-2X/6-311+G(3d,p): *ν*_1_ = 14.0 cm^−1^; *ν*_4_ = 24.2 cm^−1^; *ν*_5_ = 29.3 cm^−1^; *ν*_11_ = 81.2 cm^−1^)							
^20^H_cape_-∗-^18’^H_cape_	2.5423	0.0033	68.9	14.61	75.5	24.6	*p*-CS/vdW
^20^H_cape_-∗-^20’^H_cape_	2.7155	0.0024	70.0	27.90	72.7	36.5	*p*-CS/vdW
^20^H_cape_-∗-^2’^C_cape_	2.6769	0.0032	71.4	10.01	73.3	125.7	*p*-CS/vdW
^18^H_cape_-∗-^3’^C_cape_	2.9640	0.0016	73.8	17.36	73.2	181.8	*p*-CS/vdW
**7**:**7** (M06-2X/6-311+G(2d,p): *ν*_1_ = 13.0 cm^−1^; *ν*_4_ = 22.1 cm^−1^; *ν*_5_ = 27.3 cm^−1^; *ν*_11_ = 78.6 cm^−1^)							
^20^H_cape_-∗-^18’^H_cape_	2.5643	0.0032	67.9	16.63	75.2	60.1	*p*-CS/vdW
^20^H_cape_-∗-^20’^H_cape_	2.7287	0.0024	68.6	29.20	71.9	42.2	*p*-CS/vdW
^20^H_cape_-∗-^2’^C_cape_	2.6881	0.0031	70.2	11.44	69.4	12.4	*p*-CS/vdW
^18^H_cape_-∗-^3’^C_cape_	2.9857	0.0016	73.0	31.43	73.2	181.9	*p*-CS/vdW
**7**:**7** (LC-ωPBE/6-311+G(2d,p): *ν*_1_ = 2.1 cm^−1^; *ν*_5_ = 15.8 cm^−1^; *ν*_6_ = 28.8 cm^−1^; *ν*_8_ = 48.2 cm^−1^) ^5,6^							
^20^C_cape_-∗-^18’^H_cape_	3.2357	0.0015	63.8	65.63	67.4	40.4	*p*-CS/vdW
^20^H_cape_-∗-^20’^H_cape_	3.0148	0.0014	63.6	96.42	66.9	90.7	*p*-CS/vdW
^20^H_cape_-∗-^2’^C_cape_	3.0656	0.0014	67.2	52.66	70.0	10.9	*p*-CS/vdW
^18^H_cape_-∗-^3’^C_cape_	3.6594	0.0005	62.9	73.99	73.9	394.0	*p*-CS/vdW

^1^ See footnotes of Table 1 for the QTAIM-DFA parameters and *C_ii_*. ^2^ The motions of the internal vibrations are shown in Figure 7 and Figure S9 of the Supporting Information. ^3^ BPs and BCPs were not detected for ^1^H_bay_-∗-^6^C_bay_ and ^2^C_bay_-∗-^7^C_bay_. ^4^ *r*(^1^H_bay_-∗-^6^C_bay_) = 2.5609 Å and *r*(^2^C_bay_-∗-^7^C_bay_) = 3.2265 Å. ^5^ BPs and BCPs were not detected for ^20^H_cape_-∗-^18’^H_cape_. ^6^ *r*(^20^H_cape_-∗-^18’^H_cape_) = 2.9473 Å.

## Data Availability

Data are contained within the article or the Supplementary Materials.

## Supplementary Materials

The following are available online at https://www.mdpi.com/article/10.3390/nano12030321/s1. Table S1: The observed and calculated C···C length (*r*_obsd_ and *r*_calcd_, respectively), which are located in the bay area for **3**–**5** and the bay and cape area between adjacent aromatic rings for **6**–**12**, together with the differences, Δ*r*_calcd_ (=*r*_calcd:XY_ − *r*_obsd:XY_) in each C···C for **3**–**12**, elucidated with various methods. Table S2: The total energy *E*_ES_ and zero-point energy *E*_ZP_ values for ***n***; ***n*_p_**, where *n* = 1 to 12, along with the Δ*E*_ES_ and Δ*E*_ZP_(***n***; ***n*_p_**) values, evaluated with M06-2X/6-311+G(3d,p). Table S3: The HOMA indices for [*n*]acenes, [*n*]phenacenes, and [*n*]helicenes, evaluated with M06-2X/6-311+G(3d,p). Table S4: The observed and calculated X-∗-Y lengths (*R*_obsd:XY_ and *R*_calcd:XY_, respectively; X, Y = C and H) and the length of the bond paths (*r*_BP:XY_) and the corresponding straight-line distances (*R*_SL:XY_), together with the differences, Δ*R*_calcd:XY_ (=*R*_calcd:XY_ – *R*_obsd:XY_) in each X-∗-Y for **3**–**12**, **6**:**6** and **7**:**7,** evaluated with M06-2X/6-311+G(3d,p), where *R*_SL:XY_ = *R*_calcd:XY_, together with **8**:**8** and **10**:**10,** calculated with M06-2X/6-311+G(2d,p). Table S5: QTAIM functions and QTAIM-DFA parameters for the fused benzene-type helicenes of monomers (**3**–**12** (*C*_2_)), together with the nature of each noncovalent interaction, elucidated with M06-2X/6-311+G(2d,p). Table S6: QTAIM functions and QTAIM-DFA parameters for the fused benzene-type helicenes of monomers (**3**–**12** (*C*_2_)), along with the nature of each noncovalent interaction, elucidated with LC-ωPBE/6-311+G(2d,p). Table S7: The *E*_ES_ (au), Δ*E*_ES_ (kJ mol^−1^), and Δ*E*_ZP_ (kJ mol^−1^) values for **6**:**6**–**8**:**8** and **10**:**10**, evaluated with various methods. Table S8: QTAIM functions and QTAIM-DFA parameters for the concave-type dimer of helicenes (**8**:**8**–**10**:**10** (*C*_i_)), together with the nature of each noncovalent interaction, elucidated with M06-2X/6-311+G(2d,p). Figure S1: Plots of Δ*E*_ZP_(***n***) versus Δ*E*_ES_(***n***), calculated with M06-2X/6-311+G(3d,p). Figure S2: Plots of HOMA indices for [*n*]acenes (*n* = 4–12) at closed-shell singlet state, [*n*]acenes (*n* = 7–12) at open-shell singlet state, and [*n*]phenacenes (*n* = 4–12) versus those for [*n*]helicenes (*n* = 4–12), calculated with MP2/6-311+G(3d,p). Figure S3: Molecular graphs for **3**–**6**, **8**, and **10** calculated with M06-2X/6-311+G(3d,p). Figure S4: Plots of *r*_BP_ versus *R*_SL_ for H_bay_-∗-H_bay_, C_bay_-∗-H_bay_, C_bay_-∗-C_bay_, and C_cape_-∗-C_cape_ in **3**–**12**, calculated with M06-2X/6-311+G(3d,p). Figure S5: Plots of *H*_b_(***r***_c_) versus *H*_b_(***r***_c_) − *V*_b_(***r***_c_)/2 for H-∗-H, C-∗-H, and C-∗-C for **7**, **9**, and **11**. Figure S6: Molecular graphs for **8**:**8** (*C*_i_) and **10**:**10** (*C*_i_) calculated with M06-2X/6-311+G(2d,p). Figure S7: Plots of *H*_b_(***r***_c_) versus *H*_b_(***r***_c_) − *V*_b_(***r***_c_)/2 for H-∗-H, C-∗-H, and C-∗-C for **8**:**8** and **10**:**10**. Figure S8: The internal vibration motions of *ν_n_* for **7**:**7** (*C*_i_) from the top view. Figure S9: The internal vibration motions of *ν_n_* for **7**:**7** (*C*_i_).
