# Supplementary material for: Intrinsic Dynamic and Static Nature of π···π Interactions in Fused Benzene-Type Helicenes and Dimers, Elucidated with QTAIM Dual Functional Analysis
Source: Nanomaterials (Basel). 2022 Jan 19;12(3):321. doi: 10.3390/nano12030321 (PMC8838236; doi:10.3390/nano12030321)
Supplement: Supplementary file 1 [file nanomaterials-12-00321-s001.zip › nanomaterials-1532663-supplementary.pdf]

nanomaterials

MDPI

*Supplementary Information*

# Intrinsic Dynamic and Static Nature of π···π Interactions in Fused Benzene–Type Helicenes and Dimers, Elucidated with QTAIM Dual Functional Analysis

**Taro Nishide and Satoko Hayashi ***

Faculty of Systems Engineering, Wakayama University, 930 Sakaedani, Wakayama 640–8510, Japan; s209004@wakayama–u.ac.jp
* Correspondence: hayashi3@sys.wakayama–u.ac.jp; Tel.: +81–73–457–8252

**Table S1.** The observed and calculated C···C length ($r_{obsd}$ and $r_{calcd}$, respectively), which are located in the bay area for **3–5** and the bay and cape area between adjacent aromatic rings for **6–12**, together with the differences, $\Delta r_{calcd}$ (= $r_{calcd:XY} - r_{obsd:XY}$) in each C···C for **3–12**, elucidated with various methods.

| Compds | $r_{obsd}$/Å [a] | $r_{calcd}$/Å [b] | $\Delta r_{calcd}$/Å [c] | $r_{calcd}$/Å [b] | $\Delta r_{calcd}$/Å [c] | $r_{calcd}$/Å [b] | $\Delta r_{calcd}$/Å [c] |
|---|---|---|---|---|---|---|---|
| C···C | | M06–2X/6–311+G(3d,p) | | M06–2X/6–311+G(2d,p) | | LC–ωPBE/6–311+G(2d,p) | |
| **3** | | | | | | | |
| $^{1}C_{bay}\cdots{}^{4}C_{bay}$ | 2.9574 | 2.9860 | 0.0286 | 2.9842 | 0.0268 | 2.9763 | 0.0189 |
| **4** | | | | | | | |
| $^{1}C_{bay}\cdots{}^{5}C_{bay}$ | 3.0387 | 3.0252 | –0.0134 | 3.0237 | –0.0149 | 3.0175 | –0.0212 |
| **5** | | | | | | | |
| $^{1}C_{bay}\cdots{}^{6}C_{bay}$ | 2.9216 | 2.9191 | –0.0025 | 2.9212 | –0.0004 | 2.9189 | –0.0027 |
| $^{1}C_{cape}\cdots{}^{16}C_{cape}$ | 4.5765 | 4.5735 | –0.0030 | 4.5823 | 0.0058 | 4.5798 | 0.0034 |
| **6** | | | | | | | |
| $^{1}C_{bay}\cdots{}^{7}C_{bay}$ | 3.2496 | 3.0813 | –0.1683 | 3.0862 | –0.1634 | 3.1262 | –0.1234 |
| $^{1}C_{cape}\cdots{}^{19}C_{cape}$ | 4.6285 | 4.1555 | –0.4729 | 4.1751 | –0.4534 | 4.3244 | –0.3041 |
| **7** | | | | | | | |
| $^{1}C_{bay}\cdots{}^{7}C_{bay}$ | 3.1120 | 3.1234 | 0.0114 | 3.1276 | 0.0155 | 3.2265 | 0.1145 |
| $^{1}C_{cape}\cdots{}^{19}C_{cape}$ | 3.8256 | 3.7521 | –0.0736 | 3.7682 | –0.0574 | 4.0750 | 0.2493 |
| $^{2}C_{cape}\cdots{}^{20}C_{cape}$ | 4.4119 | 4.2392 | –0.1727 | 4.2704 | –0.1415 | 4.7973 | 0.3854 |
| **8** | | | | | | | |
| $^{1}C_{bay}\cdots{}^{7}C_{bay}$ | *d* | 3.1381 | | 3.1402 | | 3.1914 | |
| $^{2}C_{bay}\cdots{}^{8}C_{bay}$ | *d* | 3.2329 | | 3.2363 | | 3.3357 | |
| $^{1}C_{cape}\cdots{}^{19}C_{cape}$ | *d* | 3.6197 | | 3.6271 | | 3.8147 | |
| $^{2}C_{cape}\cdots{}^{20}C_{cape}$ | *d* | 3.9039 | | 3.9183 | | 4.2797 | |
| $^{3}C_{cape}\cdots{}^{21}C_{cape}$ | *d* | 3.8336 | | 3.8495 | | 4.2516 | |
| $^{4}C_{cape}\cdots{}^{22}C_{cape}$ | *d* | 3.5915 | | 3.6019 | | 3.8866 | |
| **9** | | | | | | | |
| $^{1}C_{bay}\cdots{}^{7}C_{bay}$ | 3.1722 | 3.1704 | –0.0018 | 3.1721 | –0.0001 | 3.2160 | 0.0438 |
| $^{2}C_{bay}\cdots{}^{8}C_{bay}$ | 3.2816 | 3.2510 | –0.0306 | 3.2546 | –0.0270 | 3.3437 | 0.0620 |
| $^{1}C_{cape}\cdots{}^{19}C_{cape}$ | 3.7598 | 3.6490 | –0.1109 | 3.6551 | –0.1047 | 3.8212 | 0.0614 |
| $^{2}C_{cape}\cdots{}^{20}C_{cape}$ | 4.1287 | 3.8984 | –0.2303 | 3.9105 | –0.2182 | 4.2264 | 0.0977 |
| $^{3}C_{cape}\cdots{}^{21}C_{cape}$ | 3.9874 | 3.7392 | –0.2482 | 3.7529 | –0.2345 | 4.0909 | 0.1034 |
| $^{4}C_{cape}\cdots{}^{22}C_{cape}$ | 3.6385 | 3.4874 | –0.1511 | 3.4973 | –0.1413 | 3.7322 | 0.0937 |
| $^{5}C_{cape}\cdots{}^{23}C_{cape}$ | 3.7783 | 3.5574 | –0.2209 | 3.5739 | –0.2044 | 3.9030 | 0.1247 |
| **10** | | | | | | | |

| | | | | | | | |
|---|---|---|---|---|---|---|---|
| $^{1}C_{bay}\cdots{}^{7}C_{bay}$ | 3.1762 | 3.1650 | −0.0112 | 3.1682 | −0.0080 | 3.2230 | 0.0468 |
| $^{2}C_{bay}\cdots{}^{8}C_{bay}$ | 3.3432 | 3.2954 | −0.0478 | 3.2995 | −0.0436 | 3.3860 | 0.0428 |
| $^{3}C_{bay}\cdots{}^{9}C_{bay}$ | 3.3756 | 3.2894 | −0.0862 | 3.2929 | −0.0827 | 3.3659 | −0.0097 |
| $^{1}C_{cape}\cdots{}^{19}C_{cape}$ | 3.6952 | 3.6328 | −0.0624 | 3.6407 | −0.0545 | 3.8208 | 0.1256 |
| $^{2}C_{cape}\cdots{}^{20}C_{cape}$ | 4.0115 | 3.9069 | −0.1046 | 3.9210 | −0.0905 | 4.2513 | 0.2398 |
| $^{3}C_{cape}\cdots{}^{21}C_{cape}$ | 3.9003 | 3.7707 | −0.1295 | 3.7857 | −0.1146 | 4.1196 | 0.2193 |
| $^{4}C_{cape}\cdots{}^{22}C_{cape}$ | 3.6520 | 3.5250 | −0.1269 | 3.5349 | −0.1170 | 3.7505 | 0.0985 |
| $^{5}C_{cape}\cdots{}^{23}C_{cape}$ | 3.8432 | 3.5855 | −0.2578 | 3.5985 | −0.2447 | 3.8632 | 0.0200 |
| $^{6}C_{cape}\cdots{}^{24}C_{cape}$ | 3.8282 | 3.5254 | −0.3028 | 3.5362 | −0.2919 | 3.7621 | −0.0660 |
| $^{7}C_{cape}\cdots{}^{25}C_{cape}$ | 3.6419 | 3.4321 | −0.2098 | 3.4387 | −0.2032 | 3.5921 | −0.0498 |
| **11** | | | | | | | |
| $^{1}C_{bay}\cdots{}^{7}C_{bay}$ | 3.0854 | 3.1484 | 0.0629 | 3.1517 | 0.0663 | 3.2017 | 0.1162 |
| $^{2}C_{bay}\cdots{}^{8}C_{bay}$ | 3.3389 | 3.2965 | −0.0424 | 3.3015 | −0.0374 | 3.3913 | 0.0525 |
| $^{3}C_{bay}\cdots{}^{9}C_{bay}$ | 3.4422 | 3.3277 | −0.1144 | 3.3312 | −0.1109 | 3.4041 | −0.0380 |
| $^{1}C_{cape}\cdots{}^{19}C_{cape}$ | 3.5310 | 3.6144 | 0.0834 | 3.6229 | 0.0919 | 3.7677 | 0.2367 |
| $^{2}C_{cape}\cdots{}^{20}C_{cape}$ | 3.9002 | 3.8963 | −0.0039 | 3.9109 | 0.0107 | 4.1924 | 0.2921 |
| $^{3}C_{cape}\cdots{}^{21}C_{cape}$ | 3.8595 | 3.7663 | −0.0932 | 3.7828 | −0.0767 | 4.0909 | 0.2315 |
| $^{4}C_{cape}\cdots{}^{22}C_{cape}$ | 3.6578 | 3.5279 | −0.1300 | 3.5395 | −0.1183 | 3.7550 | 0.0972 |
| $^{5}C_{cape}\cdots{}^{23}C_{cape}$ | 3.9128 | 3.6038 | −0.3089 | 3.6194 | −0.2934 | 3.8931 | −0.0197 |
| $^{6}C_{cape}\cdots{}^{24}C_{cape}$ | 3.9267 | 3.5461 | −0.3806 | 3.5580 | −0.3686 | 3.7954 | −0.1312 |
| $^{7}C_{cape}\cdots{}^{25}C_{cape}$ | 3.7478 | 3.4523 | −0.2955 | 3.4583 | −0.2895 | 3.6140 | −0.1339 |
| $^{8}C_{cape}\cdots{}^{26}C_{cape}$ | 3.9754 | 3.5190 | −0.4564 | 3.5258 | −0.4496 | 3.7333 | −0.2420 |
| **12** | | | | | | | |
| $^{1}C_{bay}\cdots{}^{7}C_{bay}$ | *d* | 3.1408 | | 3.1436 | | 3.2004 | |
| $^{2}C_{bay}\cdots{}^{8}C_{bay}$ | *d* | 3.2687 | | 3.2722 | | 3.3704 | |
| $^{3}C_{bay}\cdots{}^{9}C_{bay}$ | *d* | 3.3218 | | 3.3256 | | 3.4091 | |
| $^{4}C_{bay}\cdots{}^{10}C_{bay}$ | *d* | 3.3609 | | 3.3646 | | 3.4395 | |
| $^{1}C_{cape}\cdots{}^{19}C_{cape}$ | *d* | 3.6102 | | 3.6173 | | 3.7649 | |
| $^{2}C_{cape}\cdots{}^{20}C_{cape}$ | *d* | 3.8752 | | 3.8865 | | 4.1672 | |
| $^{3}C_{cape}\cdots{}^{21}C_{cape}$ | *d* | 3.7273 | | 3.7383 | | 4.0410 | |
| $^{4}C_{cape}\cdots{}^{22}C_{cape}$ | *d* | 3.4895 | | 3.4967 | | 3.7129 | |
| $^{5}C_{cape}\cdots{}^{23}C_{cape}$ | *d* | 3.5687 | | 3.5783 | | 3.8538 | |
| $^{6}C_{cape}\cdots{}^{24}C_{cape}$ | *d* | 3.5248 | | 3.5337 | | 3.7794 | |
| $^{7}C_{cape}\cdots{}^{25}C_{cape}$ | *d* | 3.4478 | | 3.4539 | | 3.6199 | |
| $^{8}C_{cape}\cdots{}^{26}C_{cape}$ | *d* | 3.5330 | | 3.5414 | | 3.7592 | |
| $^{9}C_{cape}\cdots{}^{27}C_{cape}$ | *d* | 3.5397 | | 3.5481 | | 3.7592 | |
| $^{10}C_{cape}\cdots{}^{28}C_{cape}$ | *d* | 3.4744 | | 3.4806 | | 3.6294 | |
| $r_{MAE}$/Å [e] | | | 0.1419 | | 0.1344 | | 0.1167 |

[a] Observed values for C···C length. [b] Calculated values for C···C length. [c] $\Delta r_{calcd} = r_{obsd} - r_{calcd}$. [d] No X–ray crystal structure. [e] The mean absolute error, defined by $[1/n\sum_i^n |r_{obsd} - r_{calcd}|]$.

**Table S2.** The total energy $E_{ES}$ and zero–point energy $E_{ZP}$ values for $\boldsymbol{n}$; $\boldsymbol{n}_P$, where $n$ = 1 to 12, along with the $\Delta E_{ES}$ and $\Delta E_{ZP}(\boldsymbol{n}; \boldsymbol{n}_P)$ values, evaluated with M06–2X/6–311+G(3d,p).

| [$n$] | $E_{ES}(\boldsymbol{n})$[a] (au) | $E_{ZP}(\boldsymbol{n})$[b] (au) | $E_{ES}(\boldsymbol{n}_P)$[c] (au) | $E_{ZP}(\boldsymbol{n}_P)$[d] (au) |
|---|---|---|---|---|
| 1 | –232.204406 | –232.103648 | –232.204406 | –232.103648 |
| 2 | –385.826436 | –385.678728 | –385.826436 | –385.678728 |
| 3 | –539.451928 | –539.257087 | –539.451928 | –539.257087 |
| 4 | –693.065985 | –692.823727 | –693.074499 | –692.832694 |
| 5 | –846.682614 | –846.393864 | –846.697891 | –846.409144 |
| 6 | –1000.301653 | –999.966434 | –1000.321148 | –999.985238 |
| 7 | –1153.921671 | –1153.538937 | –1153.944463 | –1153.561852 |
| 8 | –1307.543065 | –1307.113731 | –1307.567690 | –1307.137460 |
| 9 | –1461.164313 | –1460.688171 | –1461.190980 | –1460.714495 |
| 10 | –1614.784600 | –1614.261465 | –1614.814222 | –1614.290352 |
| 11 | –1768.405064 | –1767.835590 | –1768.437538 | –1767.867216 |
| 12 | –1922.025696 | –1921.408804 | –1922.060826 | –1921.443585 |
| [$n$] | $\Delta E_{ES}(\boldsymbol{n})$[a] (au) | $\Delta E_{ZP}(\boldsymbol{n})$[b] (au) | $\Delta E_{ES}(\boldsymbol{n}_P)$[c] (au) | $\Delta E_{ZP}(\boldsymbol{n}_P)$[d] (au) |
| 1 | | | | |
| 2 | –153.622030 | –153.575080 | –153.622030 | –153.575080 |
| 3 | –153.625492 | –153.578359 | –153.625492 | –153.578359 |
| 4 | –153.614057 | –153.566640 | –153.622571 | –153.575607 |
| 5 | –153.616629 | –153.570137 | –153.623392 | –153.576450 |
| 6 | –153.619039 | –153.572570 | –153.623257 | –153.576094 |
| 7 | –153.620018 | –153.572503 | –153.623315 | –153.576614 |
| 8 | –153.621394 | –153.574794 | –153.623227 | –153.575608 |
| 9 | –153.621248 | –153.574440 | –153.623290 | –153.577035 |
| 10 | –153.620287 | –153.573294 | –153.623242 | –153.575857 |
| 11 | –153.620464 | –153.574125 | –153.623316 | –153.576864 |
| 12 | –153.620632 | –153.573214 | –153.623288 | –153.576369 |

[a] $\Delta E_{ES}(\boldsymbol{n}) = E_{ES}(\boldsymbol{n}) - E_{ES}(\boldsymbol{n} - 1)$. [b] $\Delta E_{ZP}(\boldsymbol{n}) = E_{ZP}(\boldsymbol{n}) - E_{ZP}(\boldsymbol{n} - 1)$. [c] $\Delta E_{ES}(\boldsymbol{n}_P) = E_{ES}(\boldsymbol{n}_P) - E_{ES}(\boldsymbol{n}_P - 1)$. [d] $\Delta E_{ZP}(\boldsymbol{n}_P) = E_{ZP}(\boldsymbol{n}_P) - E_{ZP}(\boldsymbol{n}_P - 1)$.

**Table S3.** The HOMA indices for [$n$]acenes, [$n$]phenacenes, and [$n$]helicenes, evaluated with M06–2X/6–311+G(3d,p).[a,b]

| [$n$] | ring[c] | Acene[d] | Acene[e] | Phenacene | Helicene |
|---|---|---|---|---|---|
| 1 | A | 0.9989 | | | |
| 2 | A | 0.8150 | | | |
| 3 | A | 0.6325 | | 0.9072 | |
| 3 | B | 0.8178 | | 0.3181 | |
| 4 | A | 0.5106 | | 0.8801 | 0.8646 |
| 4 | B | 0.7263 | | 0.6393 | 0.5836 |
| 5 | A | 0.4342 | | 0.8923 | 0.8709 |
| 5 | B | 0.6408 | | 0.5742 | 0.5402 |
| 5 | C | 0.7019 | | 0.7284 | 0.6924 |
| 6 | A | 0.3862 | | 0.8898 | 0.8789 |
| 6 | B | 0.5787 | | 0.5910 | 0.5566 |
| 6 | C | 0.6535 | | 0.6954 | 0.6587 |
| 7 | A | 0.3557 | 0.4776 | 0.8900 | 0.8807 |
| 7 | B | 0.5360 | 0.6395 | 0.5861 | 0.5807 |
| 7 | C | 0.6111 | 0.6284 | 0.7099 | 0.6784 |
| 7 | D | 0.6296 | 0.6003 | 0.6594 | 0.6311 |
| 8 | A | 0.3362 | 0.5751 | 0.8897 | 0.8740 |
| 8 | B | 0.5073 | 0.7165 | 0.5881 | 0.5872 |
| 8 | C | 0.5791 | 0.6362 | 0.7066 | 0.6948 |
| 8 | D | 0.6024 | 0.5528 | 0.6760 | 0.6495 |
| 9 | A | 0.3236 | 0.5843 | 0.8898 | 0.8783 |
| 9 | B | 0.4881 | 0.7305 | 0.5869 | 0.5664 |
| 9 | C | 0.5560 | 0.6461 | 0.7070 | 0.6953 |
| 9 | D | 0.5793 | 0.5493 | 0.6707 | 0.6689 |
| 9 | E | 0.5853 | 0.5223 | 0.6909 | 0.6655 |
| 10 | A | 0.3151 | 0.5754 | 0.8764 | 0.8745 |
| 10 | B | 0.4750 | 0.7295 | 0.5875 | 0.5944 |
| 10 | C | 0.5398 | 0.6548 | 0.7076 | 0.6777 |
| 10 | D | 0.5615 | 0.5562 | 0.6730 | 0.6666 |
| 10 | E | 0.5686 | 0.5224 | 0.6874 | 0.6824 |
| 11 | A | 0.3094 | 0.5644 | 0.8898 | 0.8775 |
| 11 | B | 0.4663 | 0.7239 | 0.5870 | 0.5635 |
| 11 | C | 0.5287 | 0.6599 | 0.7068 | 0.6804 |
| 11 | D | 0.5487 | 0.5648 | 0.6714 | 0.6541 |
| 11 | E | 0.5551 | 0.5305 | 0.6879 | 0.6842 |
| 11 | F | 0.5565 | 0.5282 | 0.6819 | 0.7037 |
| 12 | A | 0.3055 | 0.5567 | 0.8898 | 0.8763 |
| 12 | B | 0.4603 | 0.7186 | 0.5872 | 0.5638 |
| 12 | C | 0.5210 | 0.6613 | 0.7066 | 0.6729 |
| 12 | D | 0.5394 | 0.5713 | 0.6718 | 0.6546 |
| 12 | E | 0.5449 | 0.5396 | 0.6872 | 0.6691 |
| 12 | F | 0.5460 | 0.5414 | 0.6834 | 0.6997 |

[a] The HOMA index is defined by $[1 - \alpha/m\sum_i^n (R_{\mathrm{opt}} - R_i)^2]$, where $m$, α, $R_{\mathrm{opt}}$, and $R_i$ are the number of bonds considered, empirical constant for CC bonds α = 257.7, 1.388 Å for CC bonds, and running bond length, respectively. [b] The one of HOMA indices collected, due to the $D_{2h}$, $C_{2v}$ or $C_{2h}$, $C_2$ symmetry for acene, phenacene, and helicene, respectively. [c] Defined alphabetically, starting with the terminal ring, shown in Chart S1. [d] At the closed–shell singlet state. [e] At the open–shell singlet state.

**Table S4.** The observed and calculated X–∗–Y lengths ($R_{obsd:XY}$ and $R_{calcd:XY}$, respectively; X, Y = C and H) and the length of the bond paths ($r_{BP:XY}$) and the corresponding straight–line distances ($R_{SL:XY}$), together with the differences, $\Delta R_{calcd:XY}$ (= $R_{calcd:XY} - R_{obsd:XY}$) in each X–∗–Y for **3**–**12**, **6**:**6** and **7**:**7**, evaluated with M06–2X/6–311+G(3d,p), where $R_{SL:XY} = R_{calcd:XY}$, together with **8**:**8** and **10**:**10**, calculated with M06–2X/6–311+G(2d,p).

| Compd X–∗–Y | $R_{calcd:XY}$ (= $R_{SL:XY}$ [a]) (Å) | $R_{obsd:XY}$ [b] (Å) | $\Delta R_{calcd:XY}$ [c] (Å) | $r_{BP:XY}$ [d] (Å) | $\Delta r_{BP:XY}$ [e] (Å) |
|---|---|---|---|---|---|
| **3** | | | | | |
| $^{1}H_{bay}$–∗–$^{4}H_{bay}$ | 2.0020 | (2.0349) | (–0.0329) | 2.1901 | 0.1882 |
| **4** | | | | | |
| $^{1}H_{bay}$–∗–$^{5}H_{bay}$ | 1.9931 | (1.8963) | (0.0969) | 2.2127 | 0.2196 |
| **5** | | | | | |
| $^{1}H_{bay}$–∗–$^{6}H_{bay}$ | 2.5512 | (2.8170) | (–0.2658) | 2.8426 | 0.2914 |
| **6** | | | | | |
| $^{1}H_{bay}$–∗–$^{5}C_{bay}$ | 2.4796 | (2.5059) | (–0.0263) | 2.7315 | 0.2519 |
| **7** | | | | | |
| $^{1}H_{bay}$–∗–$^{6}C_{bay}$ | 2.5902 | (2.5356) | (0.0547) | 2.9988 | 0.4086 |
| $^{2}C_{bay}$–∗–$^{7}C_{bay}$ | 2.9751 | 3.0374 | –0.0623 | 3.0126 | 0.0374 |
| **8** | | | | | |
| $^{1}H_{bay}$–∗–$^{6}C_{bay}$ | 2.5193 | *f* | | 3.0250 | 0.5057 |
| $^{2}C_{bay}$–∗–$^{7}C_{bay}$ | 2.9947 | *f* | | 3.0239 | 0.0292 |
| **9** | | | | | |
| $^{1}H_{bay}$–∗–$^{5}C_{bay}$ | 2.4930 | (2.4976) | (–0.0046) | 3.2466 | 0.7535 |
| $^{2}C_{bay}$–∗–$^{7}C_{bay}$ | 2.9977 | 3.0504 | –0.0527 | 3.0258 | 0.0281 |
| $^{3}C_{bay}$–∗–$^{8}C_{bay}$ | 2.9422 | 2.9820 | –0.0398 | 2.9642 | 0.0220 |
| $^{4}C_{cape}$–∗–$^{22}C_{cape}$ | 3.4874 | 3.6385 | –0.1511 | 3.5596 | 0.0723 |
| $^{6}C_{cape}$–∗–$^{23}C_{cape}$ | 3.4400 | 3.6886 | –0.2487 | 3.5176 | 0.0776 |
| **10** | | | | | |
| $^{1}H_{bay}$–∗–$^{6}C_{bay}$ | 2.5813 | (2.5782) | (0.0031) | 2.9305 | 0.3493 |
| $^{2}C_{bay}$–∗–$^{7}C_{bay}$ | 3.0042 | 3.0427 | –0.0385 | 3.1844 | 0.1802 |
| $^{3}C_{bay}$–∗–$^{8}C_{bay}$ | 2.9889 | 3.0443 | –0.0553 | 3.0272 | 0.0383 |
| $^{6}C_{cape}$–∗–$^{23}C_{cape}$ | 3.4723 | 3.6982 | –0.2260 | 3.6152 | 0.1430 |
| $^{7}C_{cape}$–∗–$^{25}C_{cape}$ | 3.4321 | 3.6419 | –0.2098 | 3.5442 | 0.1121 |
| **11** | | | | | |
| $^{1}H_{bay}$–∗–$^{6}C_{bay}$ | 2.5795 | (2.5038) | (0.0758) | 2.9102 | 0.3306 |
| $^{2}C_{bay}$–∗–$^{7}C_{bay}$ | 2.9877 | 3.0425 | –0.0548 | 3.0686 | 0.0809 |
| $^{3}C_{bay}$–∗–$^{8}C_{bay}$ | 2.9888 | 3.1037 | –0.1149 | 3.0296 | 0.0408 |
| $^{4}C_{bay}$–∗–$^{9}C_{bay}$ | 3.0149 | 3.1655 | –0.1506 | 3.0712 | 0.0563 |
| $^{4}C_{cape}$–∗–$^{22}C_{cape}$ | 3.5279 | 3.6578 | –0.1300 | 3.8606 | 0.3328 |
| $^{6}C_{cape}$–∗–$^{23}C_{cape}$ | 3.4710 | 3.9133 | –0.4423 | 3.6022 | 0.1312 |
| $^{7}C_{cape}$–∗–$^{25}C_{cape}$ | 3.4523 | 3.7059 | –0.2536 | 3.6740 | 0.2217 |
| $^{9}C_{cape}$–∗–$^{26}C_{cape}$ | 3.4429 | 4.0056 | –0.5627 | 3.5337 | 0.0908 |
| **12** | | | | | |
| $^{1}H_{bay}$–∗–$^{6}C_{bay}$ | 2.5753 | *f* | | 2.8893 | 0.3140 |
| $^{2}C_{bay}$–∗–$^{7}C_{bay}$ | 2.9947 | *f* | | 3.0789 | 0.0842 |
| $^{3}C_{bay}$–∗–$^{8}C_{bay}$ | 2.9736 | *f* | | 3.0015 | 0.0279 |
| $^{4}C_{bay}$–∗–$^{9}C_{bay}$ | 3.0269 | *f* | | 3.1074 | 0.0805 |
| $^{4}C_{cape}$–∗–$^{22}C_{cape}$ | 3.4895 | *f* | | 3.6126 | 0.1232 |
| $^{6}C_{cape}$–∗–$^{23}C_{cape}$ | 3.4535 | *f* | | 3.5720 | 0.1185 |
| $^{7}C_{cape}$–∗–$^{25}C_{cape}$ | 3.4478 | *f* | | 3.6954 | 0.2476 |
| $^{9}C_{cape}$–∗–$^{26}C_{cape}$ | 3.4756 | *f* | | 3.5788 | 0.1032 |
| $^{10}C_{cape}$–∗–$^{28}C_{cape}$ | 3.4744 | *f* | | 3.5873 | 0.1129 |
| **6:6** | | | | | |
| $^{1}H_{bay}$–∗–$^{17'}H_{cape}$ | 2.4840 | | | 2.7464 | 0.2624 |
| $^{1}H_{cape}$–∗–$^{16'}H_{cape}$ | 2.5169 | | | 2.7517 | 0.2347 |
| $^{1}H_{cape}$–∗–$^{17'}H_{cape}$ | 2.3329 | | | 2.4122 | 0.0793 |
| $^{15}C_{cape}$–∗–$^{17'}C_{cape}$ | 3.3236 | | | 3.3712 | 0.0476 |
| $^{16}C_{cape}$–∗–$^{17'}C_{cape}$ | 3.3531 | | | 3.4838 | 0.1307 |
| **7:7** | | | | | |

| | | | | | |
|---|---|---|---|---|---|
| $^{20}H_{bay}$–*–$^{18'}H_{cape}$ | 2.5423 | | | 2.9823 | 0.4400 |
| $^{20}H_{cape}$–*–$^{20'}H_{cape}$ | 2.7155 | | | 3.1351 | 0.4196 |
| $^{18}H_{cape}$–*–$^{3'}C_{cape}$ | 2.6769 | | | 2.7251 | 0.2726 |
| $^{20}C_{cape}$–*–$^{2'}C_{cape}$ | 2.9640 | | | 3.2255 | 0.2615 |
| **8:8** | | | | | |
| $^{21}H_{cape}$–*–$^{21'}H_{cape}$ | 2.3000 | | | 2.5725 | 0.2726 |
| $^{3}C_{cape}$–*–$^{23'}H_{cape}$ | 2.8407 | | | 3.0958 | 0.2550 |
| $^{5}C_{cape}$–*–$^{21'}H_{cape}$ | 2.7037 | | | 3.0824 | 0.3787 |
| $^{21}C_{cape}$–*–$^{23'}H_{cape}$ | 3.1228 | | | 3.4954 | 0.3726 |
| **10:10** | | | | | |
| $^{21}H_{cape}$–*–$^{21'}H_{cape}$ | 2.2151 | | | 2.3799 | 0.1648 |
| $^{3}C_{cape}$–*–$^{23'}H_{cape}$ | 2.9636 | | | 3.2375 | 0.2739 |
| $^{5}C_{cape}$–*–$^{21'}H_{cape}$ | 2.6685 | | | 3.0243 | 0.3558 |
| $^{21}C_{cape}$–*–$^{23'}H_{cape}$ | 3.0891 | | | 3.1308 | 0.0417 |

[a] Straight–line distances. [b] Average values were calculated, since all X–ray crystal structures for **3**–**7**, **9**–**11** are $C_1$ symmetry. [c] $\Delta R_{calcd:XY} = R_{calcd:XY} - R_{obsd:XY}$. [d] The length of bond paths. [e] $\Delta r_{BP} = r_{BP} - R_{SL}$. [f] No X–ray crystal structure.

**Table S5.** QTAIM functions and QTAIM–DFA parameters for the fused–benzene type helicenes of monomers (**3**–**12** ($C_2$)), together with the nature of each noncovalent interaction, elucidated with M06–2X/6–311+G(2d,p).[a]

| Compds | Interaction (X–*–Y) | $\rho_b(\boldsymbol{r}_c)$ (au) | $c\nabla^2\rho_b(\boldsymbol{r}_c)$[b] (au) | $H_b(\boldsymbol{r}_c)$ (au) | $k_b(\boldsymbol{r}_c)$[c] | $R$[d] (au) | $\theta$[e] (°) | $C_{ii}$[f] (unit [i]) | $\theta_p$[g] (°) | $\kappa_p$[h] ($au^{-1}$) | Predicted Nature |
|---|---|---|---|---|---|---|---|---|---|---|---|
| **3** | $^{1}H_{bay}$–*–$^{4}H_{bay}$ | 0.0129 | 0.0060 | 0.0022 | –0.780 | 0.0064 | 70.2 | 3.25 | 73.8 | 15.4 | *p*–CS/vdW |
| **4** | $^{1}H_{bay}$–*–$^{5}H_{bay}$ | 0.0164 | 0.0078 | 0.0026 | –0.796 | 0.0082 | 71.3 | 6.66 | 75.1 | 13.7 | *p*–CS/vdW |
| **5** | $^{1}H_{bay}$–*–$^{6}C_{bay}$ | 0.0129 | 0.0063 | 0.0022 | –0.781 | 0.0066 | 70.2 | 9.88 | 81.3 | 15.3 | *p*–CS/vdW |
| **6** | $^{1}H_{bay}$–*–$^{5}C_{bay}$ | 0.0130 | 0.0060 | 0.0021 | –0.786 | 0.0063 | 70.6 | 3.83 | 64.6 | 91.9 | *p*–CS/vdW |
| **7** | $^{1}H_{bay}$–*–$^{6}C_{bay}$ | 0.0134 | 0.0063 | 0.0022 | –0.784 | 0.0067 | 70.4 | 5.49 | 81.6 | 39.9 | *p*–CS/vdW |
| | $^{2}C_{bay}$–*–$^{7}C_{bay}$ | 0.0111 | 0.0051 | 0.0019 | –0.777 | 0.0054 | 69.9 | 3.06 | 78.7 | 120.2 | *p*–CS/vdW |
| **8** | $^{1}H_{bay}$–*–$^{6}C_{bay}$ | 0.0130 | 0.0061 | 0.0022 | –0.780 | 0.0065 | 70.2 | 5.77 | 80.2 | 35.2 | *p*–CS/vdW |
| | $^{2}C_{bay}$–*–$^{7}C_{bay}$ | 0.0116 | 0.0052 | 0.0019 | –0.777 | 0.0056 | 70.0 | 2.09 | 82.9 | 12.2 | *p*–CS/vdW |
| **9** | $^{1}H_{bay}$–*–$^{5}C_{bay}$ | 0.0133 | 0.0062 | 0.0022 | –0.784 | 0.0066 | 70.4 | 3.43 | 81.3 | 91.4 | *p*–CS/vdW |
| | $^{2}C_{bay}$–*–$^{7}C_{bay}$ | 0.0111 | 0.0050 | 0.0018 | –0.777 | 0.0054 | 69.9 | 1.90 | 79.8 | 12.8 | *p*–CS/vdW |
| | $^{3}C_{bay}$–*–$^{8}C_{bay}$ | 0.0120 | 0.0054 | 0.0019 | –0.788 | 0.0057 | 70.7 | 2.09 | 82.9 | 165.1 | *p*–CS/vdW |
| | $^{4}C_{cape}$–*–$^{22}C_{cape}$ | 0.0054 | 0.0020 | 0.0008 | –0.761 | 0.0021 | 68.9 | 6.17 | 65.8 | 152.5 | *p*–CS/vdW |
| | $^{6}C_{cape}$–*–$^{23}C_{cape}$ | 0.0059 | 0.0020 | 0.0007 | –0.778 | 0.0021 | 70.0 | 11.60 | 68.6 | 5.9 | *p*–CS/vdW |
| **10** | $^{1}H_{bay}$–*–$^{6}C_{bay}$ | 0.0136 | 0.0063 | 0.0022 | –0.788 | 0.0067 | 70.7 | 5.94 | 82.0 | 50.1 | *p*–CS/vdW |
| | $^{3}C_{bay}$–*–$^{8}C_{bay}$ | 0.0112 | 0.0050 | 0.0018 | –0.776 | 0.0054 | 69.9 | 1.85 | 79.8 | 157.8 | *p*–CS/vdW |
| | $^{4}C_{cape}$–*–$^{22}C_{cape}$[j] | 0.0051 | 0.0018 | 0.0007 | –0.763 | 0.0020 | 69.0 | 7.55 | 65.2 | 505.4 | *p*–CS/vdW |
| | $^{6}C_{cape}$–*–$^{23}C_{cape}$ | 0.0059 | 0.0020 | 0.0007 | –0.780 | 0.0021 | 70.2 | 9.85 | 68.2 | 22.2 | *p*–CS/vdW |
| | $^{7}C_{cape}$–*–$^{25}C_{cape}$ | 0.0059 | 0.0022 | 0.0008 | –0.757 | 0.0023 | 68.7 | 3.93 | 66.3 | 15.2 | *p*–CS/vdW |
| **11** | $^{1}H_{bay}$–*–$^{6}C_{bay}$ | 0.0135 | 0.0063 | 0.0022 | –0.788 | 0.0067 | 70.7 | 5.50 | 81.8 | 48.2 | *p*–CS/vdW |
| | $^{2}C_{bay}$–*–$^{7}C_{bay}$[k] | 0.0114 | 0.0051 | 0.0019 | –0.772 | 0.0054 | 69.7 | 1.89 | 84.5 | 682.9 | *p*–CS/vdW |
| | $^{3}C_{bay}$–*–$^{8}C_{bay}$ | 0.0113 | 0.0050 | 0.0018 | –0.775 | 0.0054 | 69.8 | 1.97 | 80.2 | 148.8 | *p*–CS/vdW |
| | $^{4}C_{bay}$–*–$^{9}C_{bay}$ | 0.0109 | 0.0049 | 0.0018 | –0.772 | 0.0053 | 69.6 | 1.72 | 77.4 | 137.1 | *p*–CS/vdW |
| | $^{4}C_{cape}$–*–$^{22}C_{cape}$ | 0.0051 | 0.0018 | 0.0007 | –0.759 | 0.0020 | 68.8 | 7.67 | 66.6 | 18.8 | *p*–CS/vdW |
| | $^{6}C_{cape}$–*–$^{23}C_{cape}$ | 0.0058 | 0.0019 | 0.0007 | –0.778 | 0.0021 | 70.0 | 11.51 | 68.1 | 14.3 | *p*–CS/vdW |
| | $^{7}C_{cape}$–*–$^{25}C_{cape}$ | 0.0058 | 0.0021 | 0.0008 | –0.759 | 0.0023 | 68.7 | 3.91 | 66.1 | 5.2 | *p*–CS/vdW |
| | $^{9}C_{cape}$–*–$^{26}C_{cape}$ | 0.0062 | 0.0021 | 0.0008 | –0.778 | 0.0022 | 70.0 | 6.14 | 68.6 | 42.0 | *p*–CS/vdW |
| **12** | $^{1}H_{bay}$–*–$^{6}C_{bay}$ | 0.0136 | 0.0063 | 0.0022 | –0.787 | 0.0067 | 70.6 | 4.89 | 81.4 | 63.6 | *p*–CS/vdW |
| | $^{2}C_{bay}$–*–$^{7}C_{bay}$[l] | 0.0113 | 0.0050 | 0.0019 | –0.770 | 0.0054 | 69.5 | 1.79 | 84.6 | 587.9 | *p*–CS/vdW |
| | $^{3}C_{bay}$–*–$^{8}C_{bay}$ | 0.0115 | 0.0051 | 0.0018 | –0.780 | 0.0054 | 70.2 | 1.75 | 80.8 | 193.2 | *p*–CS/vdW |
| | $^{4}C_{bay}$–*–$^{9}C_{bay}$[m] | 0.0108 | 0.0048 | 0.0018 | –0.767 | 0.0052 | 69.3 | 1.73 | 78.2 | 86.8 | *p*–CS/vdW |
| | $^{4}C_{cape}$–*–$^{22}C_{cape}$ | 0.0054 | 0.0020 | 0.0008 | –0.755 | 0.0021 | 68.5 | 5.05 | 65.5 | 62.0 | *p*–CS/vdW |
| | $^{6}C_{cape}$–*–$^{23}C_{cape}$ | 0.0060 | 0.0020 | 0.0008 | –0.774 | 0.0022 | 69.7 | 7.11 | 68.0 | 33.1 | *p*–CS/vdW |
| | $^{7}C_{cape}$–*–$^{25}C_{cape}$ | 0.0058 | 0.0021 | 0.0008 | –0.756 | 0.0023 | 68.6 | 3.61 | 66.0 | 9.3 | *p*–CS/vdW |
| | $^{9}C_{cape}$–*–$^{26}C_{cape}$ | 0.0059 | 0.0020 | 0.0007 | –0.779 | 0.0021 | 70.1 | 7.28 | 68.6 | 16.2 | *p*–CS/vdW |
| | $^{10}C_{cape}$–*–$^{28}C_{cape}$ | 0.0054 | 0.0020 | 0.0008 | –0.758 | 0.0021 | 68.7 | 4.40 | 66.3 | 24.7 | *p*–CS/vdW |

[a] Data are given at BCPs. [b] $c\nabla^2\rho_b(\boldsymbol{r}_c) = H_b(\boldsymbol{r}_c) - V_b(\boldsymbol{r}_c)/2$, where $c = \hbar^2/8m$. [c] $k_b(\boldsymbol{r}_c) = V_b(\boldsymbol{r}_c)/G_b(\boldsymbol{r}_c)$. [d] $R = (x + y)^{1/2}$, where $(x, y = H_b(\boldsymbol{r}_c) - V_b(\boldsymbol{r}_c)/2, H_b(\boldsymbol{r}_c))$. [e] $\theta = 90^{\circ} - \tan^{-1}(y/x)$. [f] Compliance force constants. [g] $\theta_p = 90^{\circ} - \tan^{-1}(dy/dx)$. [h] $\kappa_p = |d^2y/dx^2|/[1 + (dy/dx)^2]^{3/2}$. [i] Å $mdyn^{-1}$. [j] Data from $w$ = –0.05, –0.0375, –0.025, –0.0125, and 0, were employed for the evaluation of dynamic nature, since BCP for $^{4}C_{cape}$–*–$^{22}C_{cape}$ in the case of $w > 0$ were not detected. [k] Data from $w$ = 0, ±0.0125, and ±0.025 were employed for the evaluation of dynamic nature, since BCP for $^{2}C_{bay}$–*–$^{7}C_{bay}$ in the case of $w > 0.05$ were not detected. [l] Data from $w$ = –0.0375, –0.025, –0.0125, 0, 0.0125 were employed for the evaluation of dynamic nature, since BCP for $^{2}C_{bay}$–*–$^{7}C_{bay}$ in the case of $w > 0.025$ were not detected. [m] Data from $w$ = –0.0375, –0.025, –0.0125, 0, 0.0125 were employed for the evaluation of dynamic nature, since BCP for $^{4}C_{bay}$–*–$^{9}C_{bay}$ in the case of $w > 0.025$ were not detected.

**Table S6.** QTAIM functions and QTAIM–DFA parameters for the fused benzene–type helicenes of monomers (**3**–**12** ($C_2$)), along with the nature of each noncovalent interaction, elucidated with LC–ωPBE/6–311+G(2d,p).[a]

| Compds | Interaction (X–*–Y) | $\rho_b(\boldsymbol{r}_c)$ (au) | $c\nabla^2\rho_b(\boldsymbol{r}_c)$[b] (au) | $H_b(\boldsymbol{r}_c)$ (au) | $k_b(\boldsymbol{r}_c)$[c] | $R$[d] (au) | $\theta$[e] (°) | $C_{ii}$[f] (unit[i]) | $\theta_p$[g] (°) | $\kappa_p$[h] ($au^{-1}$) | Predicted Nature |
|---|---|---|---|---|---|---|---|---|---|---|---|
| **3** | $^1H_{bay}$–*–$^4H_{bay}$ | 0.0132 | 0.0062 | 0.0023 | –0.776 | 0.0066 | 69.9 | 3.47 | 76.0 | 9.4 | *p*–CS/vdW |
| **4** | $^1H_{bay}$–*–$^5H_{bay}$ | 0.0169 | 0.0079 | 0.0026 | –0.800 | 0.0083 | 71.6 | 6.72 | 77.9 | 22.8 | *p*–CS/vdW |
| **5** | $^1H_{bay}$–*–$^6C_{bay}$ | 0.0130 | 0.0062 | 0.0022 | –0.778 | 0.0066 | 70.0 | 6.71 | 83.7 | 55.5 | *p*–CS/vdW |
| **6** | $^1H_{bay}$–*–$^5C_{bay}$ | 0.0134 | 0.0060 | 0.0020 | –0.793 | 0.0063 | 71.1 | 3.86 | 81.6 | 46.3 | *p*–CS/vdW |
| **7** | $^1H_{bay}$–*–$^5C_{bay}$ | 0.0139 | 0.0062 | 0.0022 | –0.789 | 0.0066 | 70.8 | 3.70 | 82.2 | 45.7 | *p*–CS/vdW |
| **8** | $^1H_{bay}$–*–$^5C_{bay}$[j] | 0.0135 | 0.0061 | 0.0021 | –0.786 | 0.0064 | 70.6 | 3.77 | 96.0 | 12303.9 | *p*–CS/*t*–$HB_{nc}$ |
| **9** | $^1H_{bay}$–*–$^5C_{bay}$ | 0.0141 | 0.0062 | 0.0021 | –0.797 | 0.0066 | 71.4 | 3.47 | 83.2 | 42.9 | *p*–CS/vdW |
| | $^3C_{bay}$–*–$^8C_{bay}$ | 0.0106 | 0.0046 | 0.0018 | –0.758 | 0.0050 | 68.7 | 2.49 | 81.2 | 176.1 | *p*–CS/vdW |
| | $^6C_{cape}$-*–$^{23}C_{cape}$ | 0.0033 | 0.0011 | 0.0005 | –0.741 | 0.0012 | 67.7 | 12.43 | 68.2 | 22.8 | *p*–CS/vdW |
| **10** | $^1H_{bay}$–*–$^6C_{bay}$ | 0.0143 | 0.0064 | 0.0022 | –0.796 | 0.0067 | 71.3 | 5.74 | 82.8 | 191.9 | *p*–CS/vdW |
| | $^3C_{bay}$–*–$^8C_{bay}$ | 0.0101 | 0.0044 | 0.0018 | –0.750 | 0.0048 | 68.2 | 2.67 | 78.3 | 323.9 | *p*–CS/vdW |
| | $^6C_{cape}$–*–$^{23}C_{cape}$ | 0.0037 | 0.0013 | 0.0005 | –0.746 | 0.0014 | 67.9 | 13.90 | 68.7 | 404.7 | *p*–CS/vdW |
| **11** | $^1H_{bay}$–*–$^6C_{bay}$ | 0.0141 | 0.0063 | 0.0021 | –0.795 | 0.0067 | 71.2 | 5.63 | 83.6 | 171.4 | *p*–CS/vdW |
| | $^3C_{bay}$–*–$^8C_{bay}$ | 0.0099 | 0.0044 | 0.0018 | –0.747 | 0.0047 | 68.0 | 2.70 | 78.4 | 174.0 | *p*–CS/vdW |
| | $^4C_{bay}$–*–$^9C_{bay}$ | 0.0097 | 0.0043 | 0.0018 | –0.745 | 0.0047 | 67.9 | 2.76 | 77.3 | 163.4 | *p*–CS/vdW |
| | $^6C_{cape}$–*–$^{24}C_{cape}$[k] | 0.0036 | 0.0013 | 0.0005 | –0.743 | 0.0014 | 67.7 | 13.86 | 70.0 | 2078.2 | *p*–CS/vdW |
| | $^9C_{cape}$–*–$^{26}C_{cape}$ | 0.0040 | 0.0014 | 0.0006 | –0.745 | 0.0015 | 67.9 | 14.90 | 68.2 | 10.0 | *p*–CS/vdW |
| **12** | $^1H_{bay}$–*–$^6C_{bay}$ | 0.0143 | 0.0064 | 0.0022 | –0.796 | 0.0067 | 71.3 | 5.68 | 81.9 | 180.6 | *p*–CS/vdW |
| | $^3C_{bay}$–*–$^8C_{bay}$ | 0.0102 | 0.0045 | 0.0018 | –0.753 | 0.0048 | 68.4 | 2.64 | 80.2 | 178.3 | *p*–CS/vdW |
| | $^6C_{cape}$–*–$^{23}C_{cape}$ | 0.0038 | 0.0013 | 0.0006 | –0.739 | 0.0014 | 67.5 | 13.59 | 67.6 | 150.4 | *p*–CS/vdW |
| | $^9C_{cape}$–*–$^{26}C_{cape}$ | 0.0039 | 0.0013 | 0.0005 | –0.743 | 0.0014 | 67.8 | 14.52 | 68.3 | 11.7 | *p*–CS/vdW |

[a] Data are given at BCPs. [b] $c\nabla^2\rho_b(\boldsymbol{r}_c) = H_b(\boldsymbol{r}_c) - V_b(\boldsymbol{r}_c)/2$, where $c = \hbar^2/8m$. [c] $k_b(\boldsymbol{r}_c) = V_b(\boldsymbol{r}_c)/G_b(\boldsymbol{r}_c)$. [d] $R = (x + y)^{1/2}$, where $(x, y = H_b(\boldsymbol{r}_c) - V_b(\boldsymbol{r}_c)/2, H_b(\boldsymbol{r}_c))$. [e] $\theta = 90º - \tan^{-1}(y/x)$. [f] Compliance force constants. [g] $\theta_p = 90º - \tan^{-1}(dy/dx)$. [h] $\kappa_p = |d^2y/dx^2|/[1 + (dy/dx)^2]^{3/2}$. [i] Å $mdyn^{-1}$. [j] No intrinsic dynamic behavior for $^1H_{bay}$–*–$^5C_{bay}$ was observed, since BCPs were detected between $^1H_{bay}$ and $^6C_{bay}$ in the case of $0.0 < w$, while if $w \le 0.0$, BCP were detected between $^1H_{bay}$ and $^5C_{bay}$. [k] Data from $w$ = –0.05, –0.0375, –0.025, –0.0125, and 0 were employed for the evaluation of dynamic nature, since BCPs for $^6C_{cape}$–*–$^{24}C_{cape}$ in the case of $w > 0$ were not detected.

**Table S7.** The $E_{ES}$ (au), $\Delta E_{ES}$ (kJ mol$^{-1}$), and $\Delta E_{ZP}$ (kJ mol$^{-1}$) values for **6**:**6**–**8**:**8** and **10**:**10**, evaluated with various methods.[a]

| Compd (symm) | $E_{ES:dim}$[b] (au) | $E_{ES:mono}$[c] (au) | $\Delta E_{ES}$[d] (kJ mol$^{-1}$) |
|---|---|---|---|
| M06–2X/6–311+G(3d,p) | | | |
| **6**:**6** ($C_i$) | –2000.613340 | –1000.301869 | –25.2 |
| **7**:**7** ($C_i$) | –2307.855458 | –1153.921673 | –31.8 |
| M06–2X/6–311+G(2d,p) | | | |
| **7**:**7** ($C_i$) | –2307.865224 | –1153.926727 | –30.9 |
| **8**:**8** ($C_i$) | –2615.109370 | –1307.548324 | –33.4 |
| **10**:**10** ($C_i$) | –3229.593523 | –1614.790462 | –33.1 |
| LC–ωPBE/6–311+G(2d,p) | | | |
| **7**:**7** ($C_i$) | –2306.940209 | –1153.468598 | –7.9 |

[a] Ultrafine integration grid (corresponding to Int=ultrafine keyword) was employed for the calculations, since very low imaginary frequencies were predicted for frequency analysis under default condition (finegrid) of the Gaussian 09 program. [b] For concave–type dimers. [c] For monomers. [d] $\Delta E_{ES} = E_{ES:dim} - 2E_{ES:mono}$.

**Table S8.** QTAIM functions and QTAIM–DFA parameters for the concave–type dimer of helicenes (**8**:**8**–**10**:**10** ($C_i$)), together with the nature of each noncovalent interaction, elucidated with M06–2X/6–311+G(2d,p).[a]

| Compds (symm) | Interaction (X–*–Y) | $\rho_b(\boldsymbol{r}_c)$ (au) | $c\nabla^2\rho_b(\boldsymbol{r}_c)$[b] (au) | $H_b(\boldsymbol{r}_c)$ (au) | $k_b(\boldsymbol{r}_c)$[c] | $R$[d] (au) | $\theta$[e] (°) | $C_{ii}$[f] (unit[i]) | $\theta_p$[g] (°) | $\kappa_p$[h] (au$^{-1}$) | Predicted Nature |
|---|---|---|---|---|---|---|---|---|---|---|---|
| **8**:**8** ($C_i$) | $^{21}H_{cape}$–*–$^{21'}H_{cape}$ | 0.0103 | 0.0045 | 0.0016 | –0.776 | 0.0048 | 69.9 | 9.12 | 75.0 | 44.8 | *p*–CS/vdW |
| | $^{3}C_{cape}$–*–$^{23'}H_{cape}$ | 0.0063 | 0.0024 | 0.0009 | –0.773 | 0.0026 | 69.7 | 14.48 | 70.6 | 14.2 | *p*–CS/vdW |
| | $^{5}C_{cape}$–*–$^{21'}H_{cape}$ | 0.0077 | 0.0032 | 0.0011 | –0.782 | 0.0034 | 70.3 | 8.83 | 70.6 | 16.2 | *p*–CS/vdW |
| | $^{21}C_{cape}$–*–$^{23'}H_{cape}$ | 0.0045 | 0.0015 | 0.0005 | –0.803 | 0.0016 | 71.8 | 24.95 | 73.2 | 197.1 | *p*–CS/vdW |
| **10**:**10** ($C_i$) | $^{21}H_{cape}$–*–$^{21'}H_{cape}$ | 0.0104 | 0.0043 | 0.0015 | –0.792 | 0.0046 | 71.0 | 7.85 | 72.5 | 39.1 | *p*–CS/vdW |
| | $^{3}C_{cape}$–*–$^{23'}H_{cape}$ | 0.0052 | 0.0020 | 0.0008 | –0.762 | 0.0021 | 69.0 | 24.13 | 71.5 | 26.9 | *p*–CS/vdW |
| | $^{5}C_{cape}$–*–$^{21'}H_{cape}$ | 0.0083 | 0.0035 | 0.0013 | –0.779 | 0.0037 | 70.1 | 7.62 | 70.3 | 2.6 | *p*–CS/vdW |
| | $^{21}C_{cape}$–*–$^{23'}H_{cape}$ | 0.0045 | 0.0016 | 0.0006 | –0.791 | 0.0017 | 70.9 | 28.07 | 76.2 | 502.6 | *p*–CS/vdW |

[a] Data are given at BCPs. [b] $c\nabla^2\rho_b(\boldsymbol{r}_c) = H_b(\boldsymbol{r}_c) - V_b(\boldsymbol{r}_c)/2$, where $c = \hbar^2/8m$. [c] $k_b(\boldsymbol{r}_c) = V_b(\boldsymbol{r}_c)/G_b(\boldsymbol{r}_c)$. [d] $R = (x + y)^{1/2}$, where $(x, y = H_b(\boldsymbol{r}_c) - V_b(\boldsymbol{r}_c)/2, H_b(\boldsymbol{r}_c))$. [e] $\theta = 90° - \tan^{-1}(y/x)$. [f] Compliance force constants. [g] $\theta_p = 90° - \tan^{-1}(dy/dx)$. [h] $\kappa_p = |d^2y/dx^2|/[1 + (dy/dx)^2]^{3/2}$. [i] Å mdyn$^{-1}$.

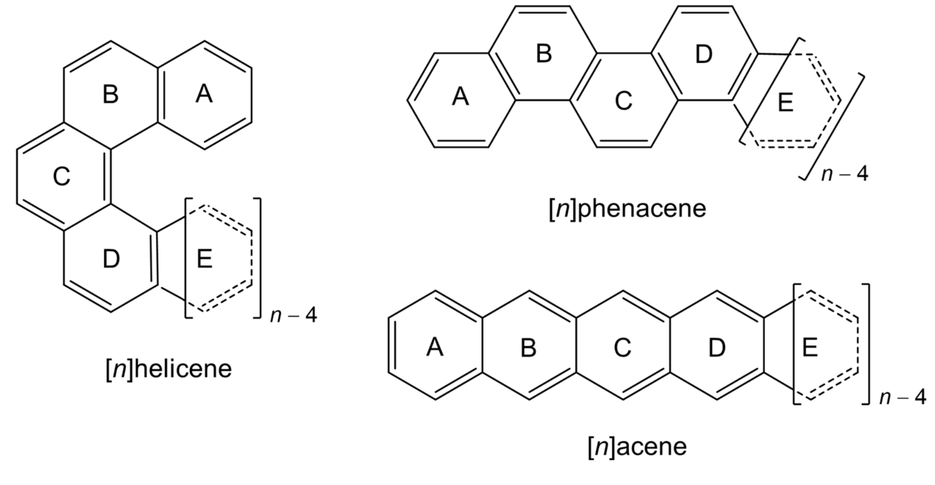


**Chart S1.** Definition for the position of aromatic rings, employed in the HOMA indices.

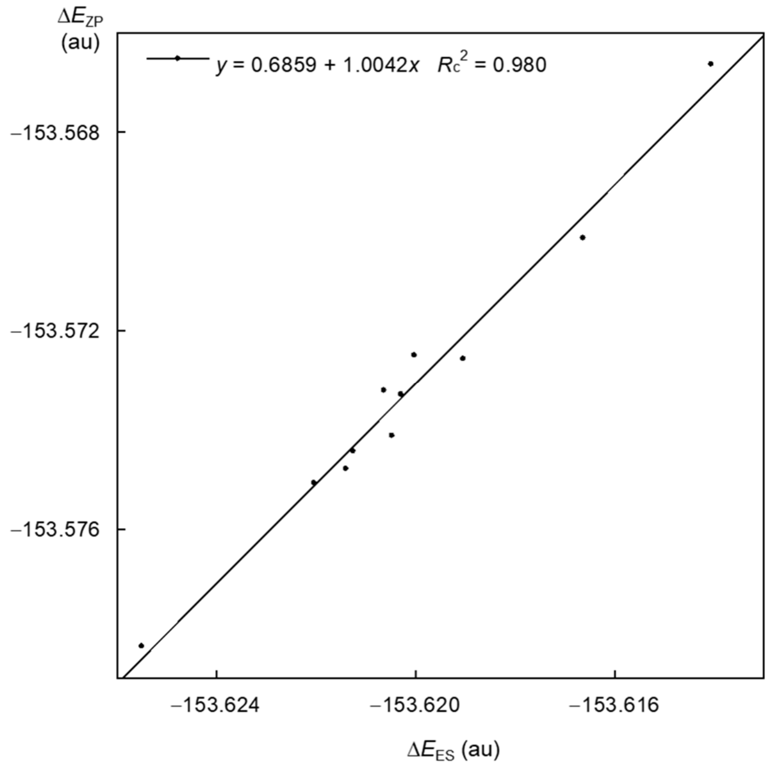


**Figure S1.** Plots of $\Delta E_{ZP}(\boldsymbol{n})$ versus $\Delta E_{ES}(\boldsymbol{n})$, calculated with M06–2X/6–311+G(3d,p), where $\Delta E_{ES}(\boldsymbol{n})$ (= $E_{ES}(\boldsymbol{n}) - E_{ES}(\boldsymbol{n} - 1)$) and $\Delta E_{ZP}(\boldsymbol{n})$ (= $E_{ZP}(\boldsymbol{n}) - E_{ZP}(\boldsymbol{n} - 1)$). Correlations are given in the figure.

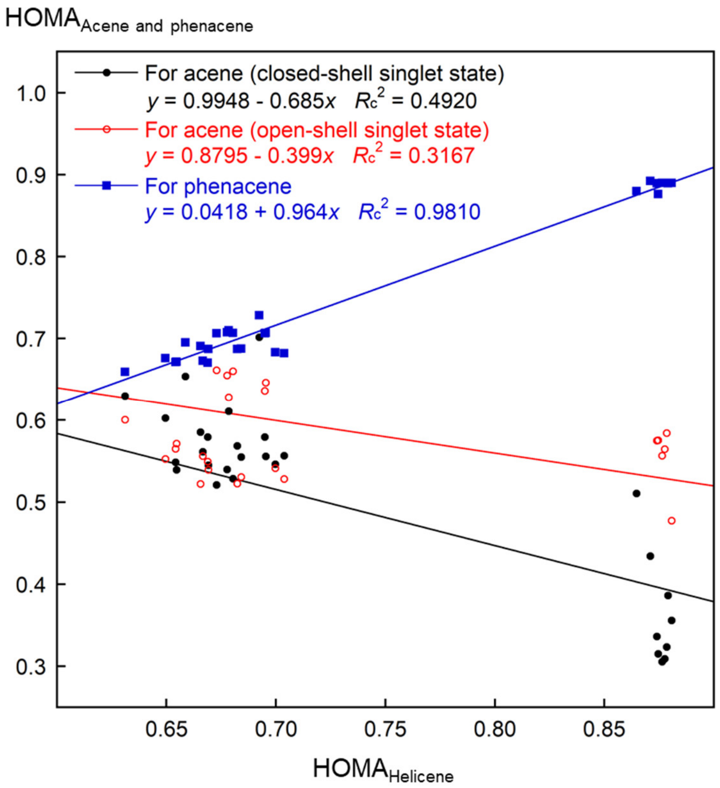


**Figure S2.** Plots of HOMA indices for [$n$]acenes ($n$ = 4–12) at closed–shell singlet state, [$n$]acenes ($n$ = 7–12) at open–shell singlet state and [$n$]phenacenes ($n$ = 4–12) versus those for [$n$]helicenes ($n$ = 4–12), calculated with MP2/6–311+G(3d,p). Correlations are given in the figure.

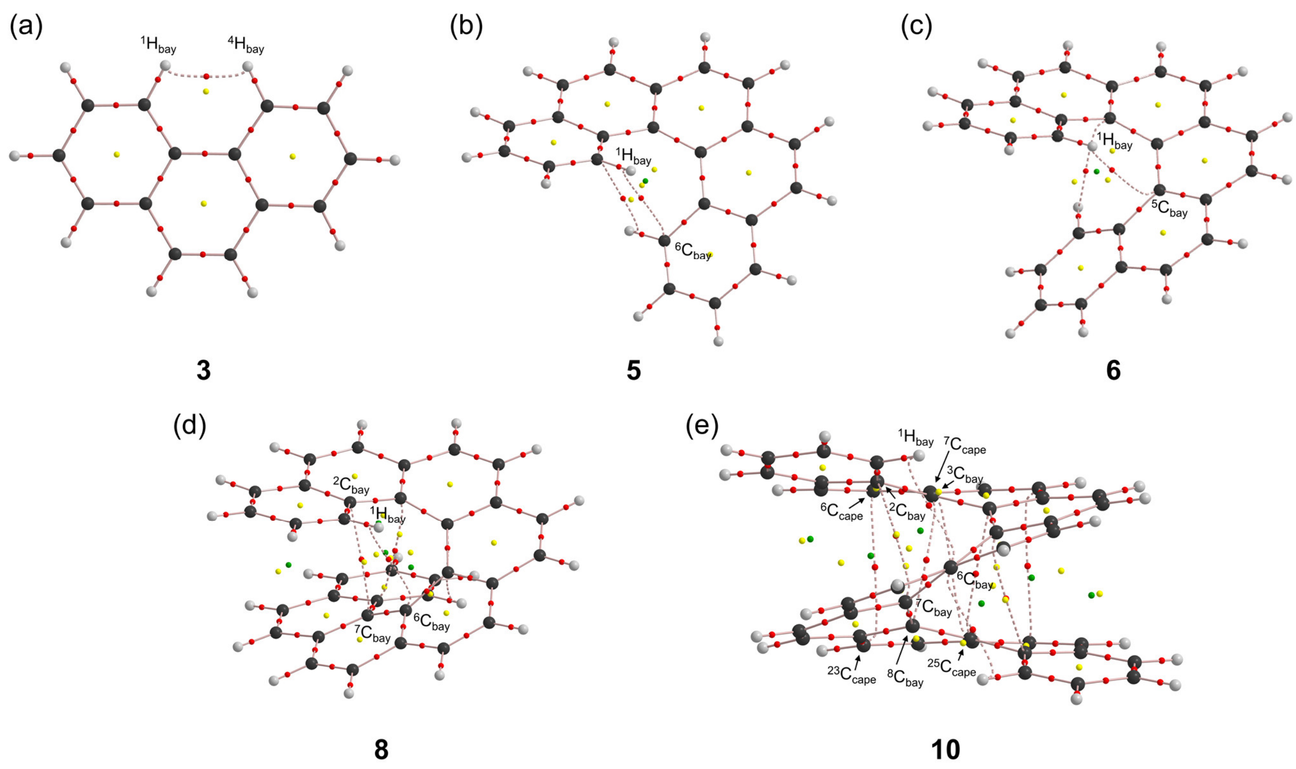


**Figure S3.** Molecular graphs for **3**–**6**, **8**, and **10** calculated with M06–2X/6–311+G(3d,p), retaining $C_2$ symmetry, (shown by (**a**–**e**), respectively, in the figure), where BPs with BCPs corresponding to intramolecular non–covalent interactions are detected. The BCPs are denoted by red dots, RCPs (ring critical points) by yellow dots, CCPs (cage critical points) by green dots and BPs by pink lines. The carbon atoms are in black with hydrogen atoms in grey.

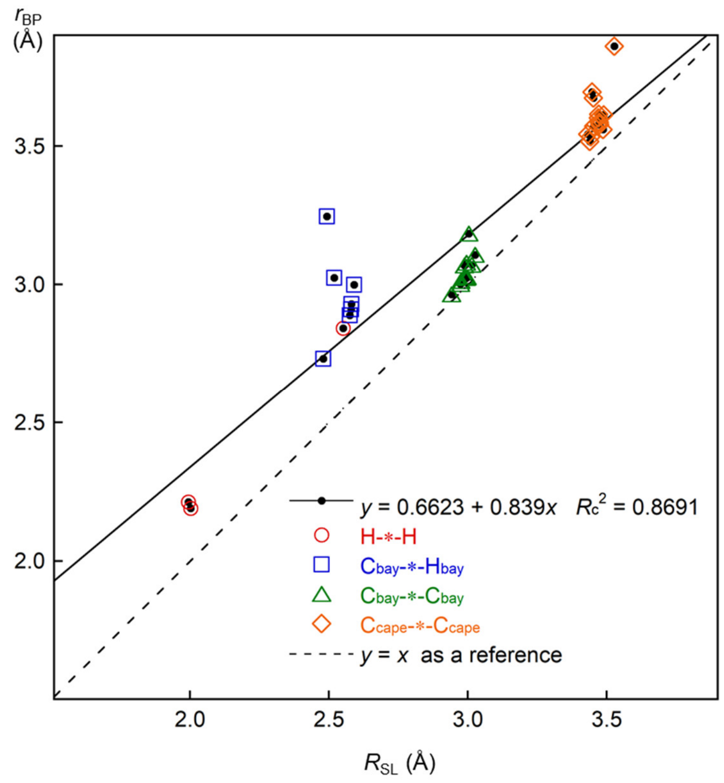


**Figure S4.** Plots of $r_{BP}$ versus $R_{SL}$ for $H_{bay}$–*–$H_{bay}$, $C_{bay}$–*–$H_{bay}$, $C_{bay}$–*–$C_{bay}$, and $C_{cape}$–*–$C_{cape}$ in **3**–**12**, calculated with M06–2X/6–311+G(3d,p). The correlation is given in the figure.

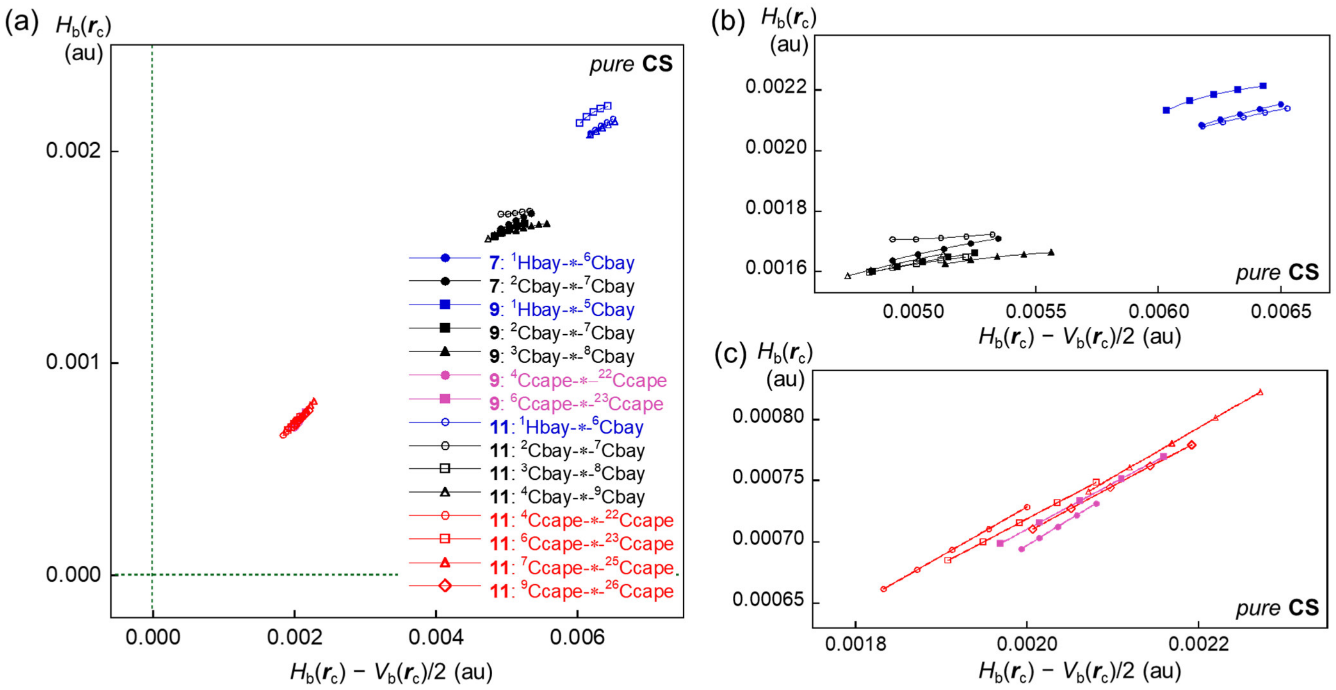


**Figure S5.** Plots of $H_b(\mathbf{r}_c)$ versus $H_b(\mathbf{r}_c) - V_b(\mathbf{r}_c)/2$ for H–∗–H, C–∗–H, and C–∗–C for **7**, **9**, and **11**. Whole picture (**a**), magnified picture for C–∗–H and C–∗–C in the bay area (**b**), magnified picture for C–∗–C in the cape area (**c**).

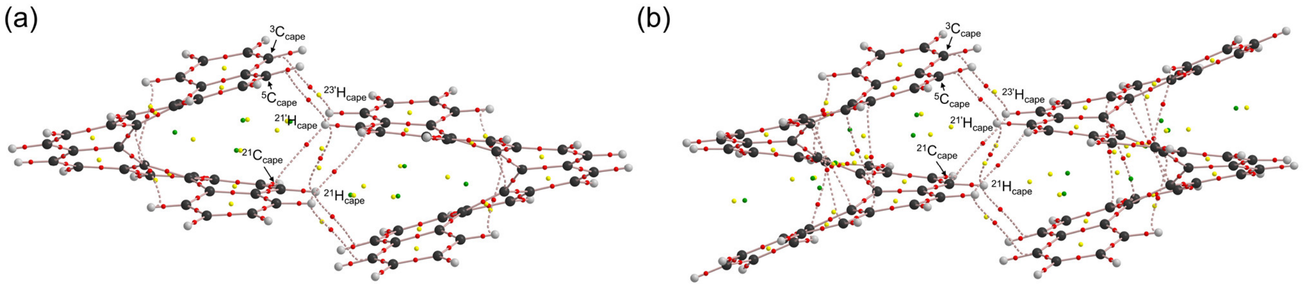


**8**:**8** ($C_i$)

**10**:**10** ($C_i$)

**Figure S6.** Molecular graphs for **8**:**8** ($C_i$) and **10**:**10** ($C_i$) calculated with M06–2X/6–311+G(2d,p) (shown by (**a**) and (**b**), respectively, in the figure), where BPs with BCPs corresponding to intramolecular non–covalent interactions are detected. The BCPs are denoted by red dots, RCPs (ring critical points) by yellow dots, CCPs (cage critical points) by green dots and BPs by pink lines. The carbon atoms are in black with hydrogen atoms in grey.

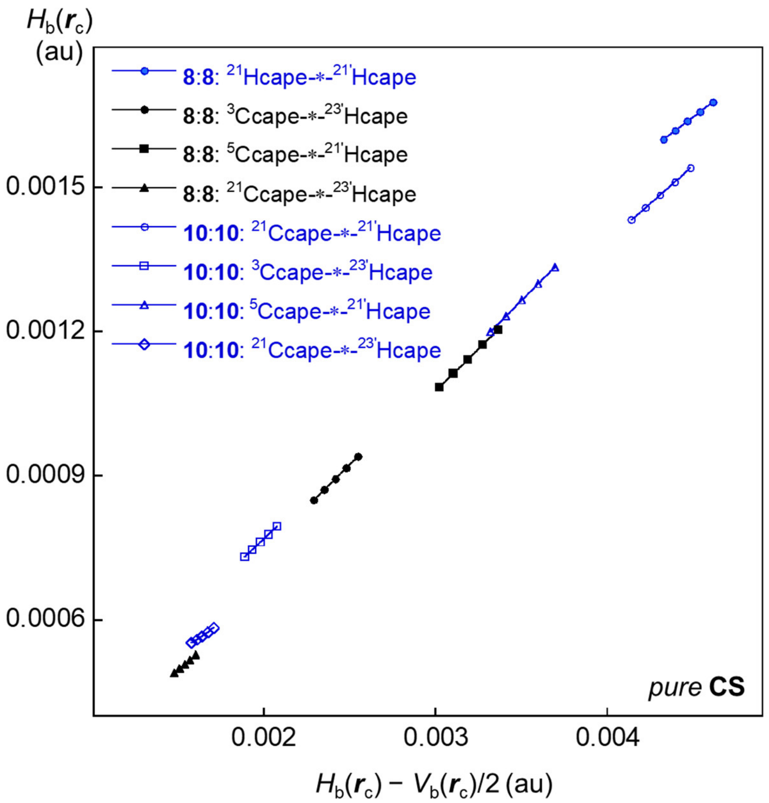


**Figure S7.** Plots of $H_b(r_c)$ versus $H_b(r_c) - V_b(r_c)/2$ for H–∗–H, C–∗–H, and C–∗–C for **8**:**8** and **10**:**10**.

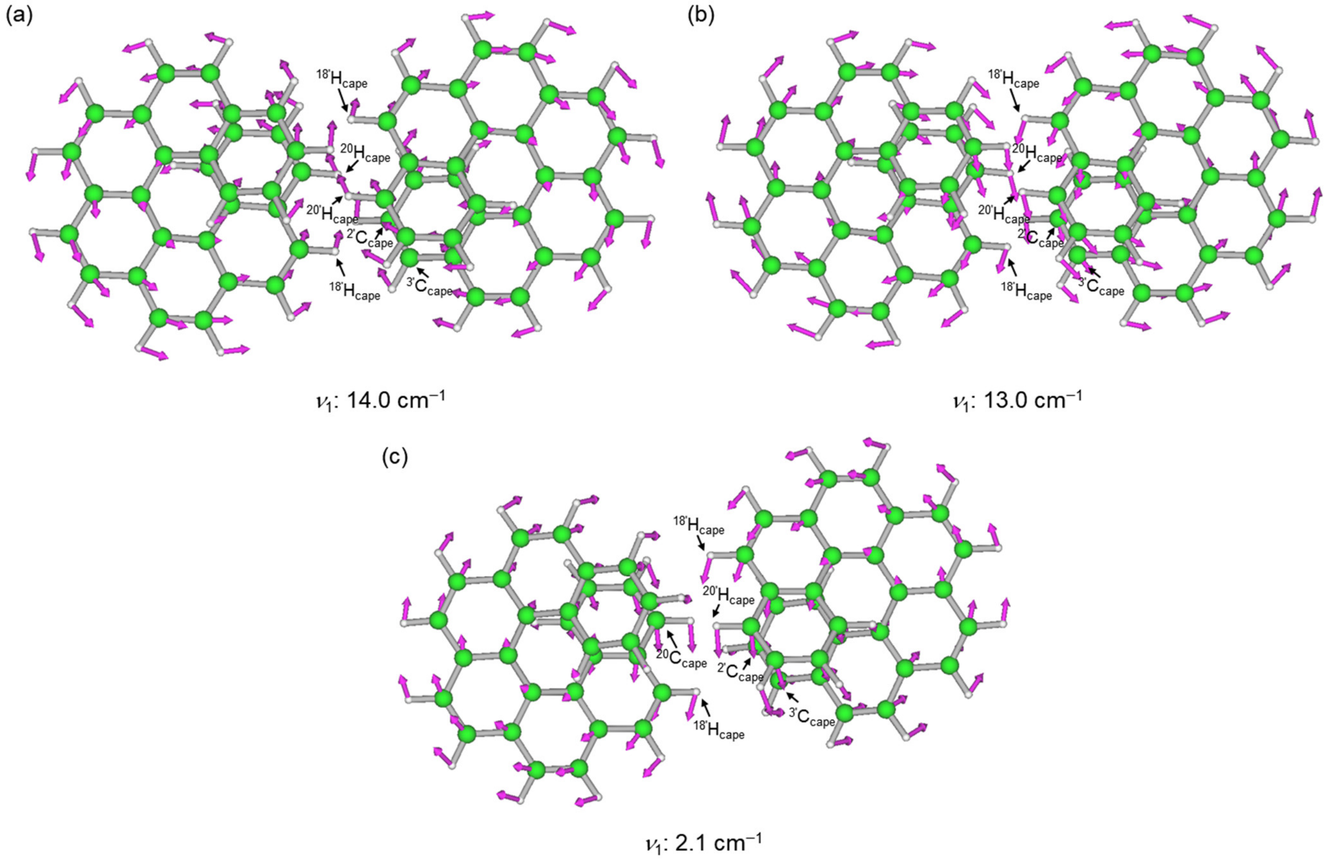


**Figure S8.** The internal vibration motions of $\nu_1$ for **7**:**7** ($C_i$) from the top view. $\nu_1$ for **7**:**7** ($C_i$) calculated with M06–2X/6–311+G(3d,p) (**a**), $\nu_1$ for **7**:**7** ($C_i$) calculated with M06–2X/6–311+G(2d,p) (**b**), $\nu_1$ for **7**:**7** ($C_i$) calculated with LC–ωPBE/6–311+G(2d,p) (**c**).

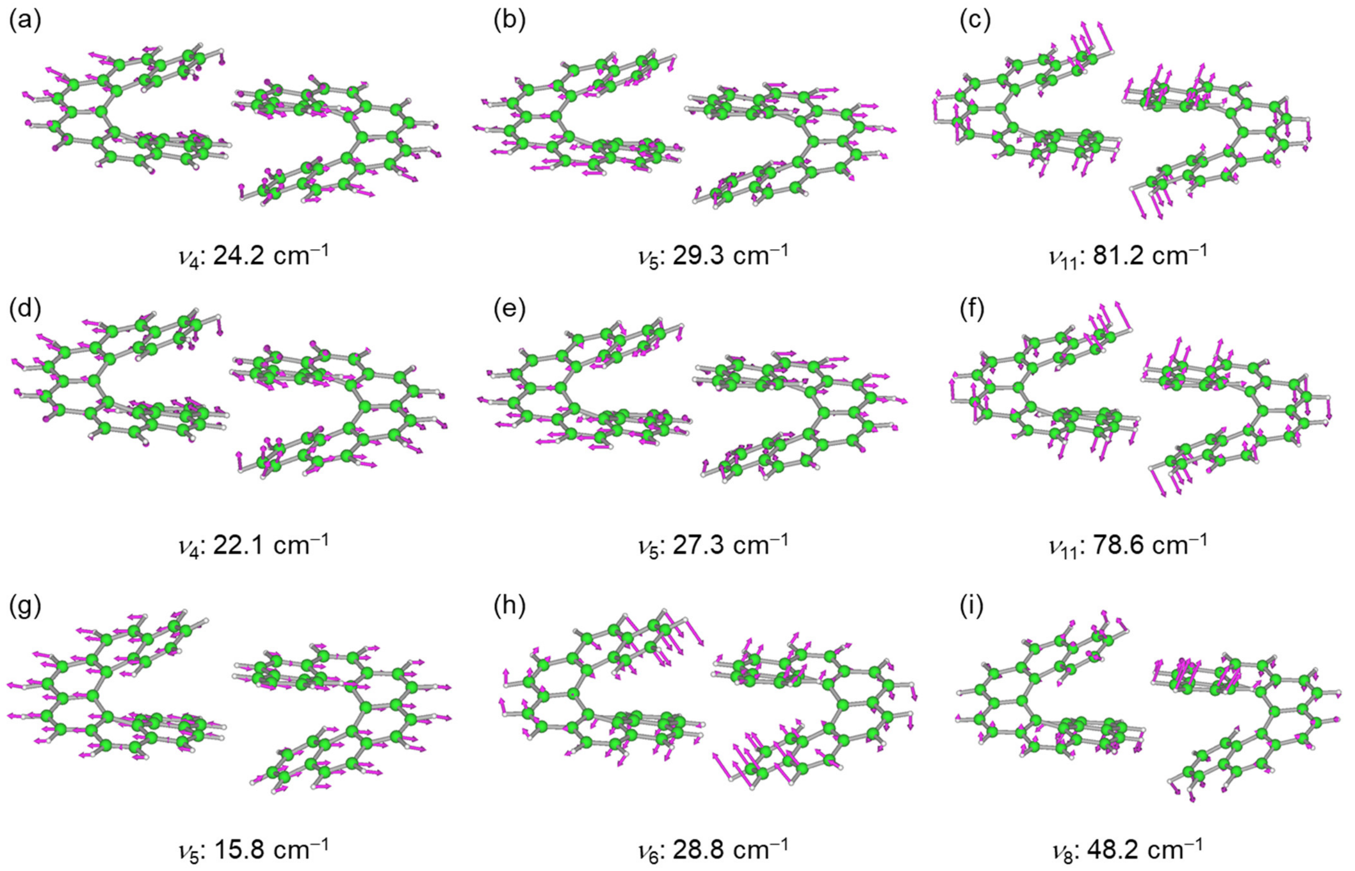


**Figure S9.** The internal vibration motions of $\nu_n$ for **7**:**7** ($C_i$). $\nu_4$ for **7**:**7** ($C_i$) calculated with M06–2X/6–311+G(3d,p) (**a**), $\nu_5$ for **7**:**7** ($C_i$) calculated with M06–2X/6–311+G(3d,p) (**b**), $\nu_{11}$ for **7**:**7** ($C_i$) calculated with M06–2X/6–311+G(3d,p) (**c**), $\nu_4$ for **7**:**7** ($C_i$) calculated with M06–2X/6–311+G(2d,p) (**d**), $\nu_5$ for **7**:**7** ($C_i$) calculated with M06–2X/6–311+G(2d,p) (**e**), $\nu_{11}$ for **7**:**7** ($C_i$) calculated with M06–2X/6–311+G(2d,p) (**f**), $\nu_5$ for **7**:**7** ($C_i$) calculated with LC–ωPBE/6–311+G(2d,p) (**g**), $\nu_6$ for **7**:**7** ($C_i$) calculated with LC–ωPBE/6–311+G(2d,p) (**h**), $\nu_8$ for **7**:**7** ($C_i$) calculated with LC–ωPBE/6–311+G(2d,p) (**i**).

## Optimized structures given by Cartesian coordinates

Optimized structures given by Cartesian coordinates for examined molecules, together with the total energies for **1**–**12**, **6**:**6** and **7**:**7**, calculated with M06–2X/6–311+G(3d,p) method of the Gaussian 09 program, together with **7**, **8**:**8**, and **10**:**10** calculated with M06–2X/6–311+G(2d,p), and **7** and **7**:**7** calculated with LC–ωPBE/6–311+G(2d,p). The optimized structures were confirmed by the frequency analysis. The ultrafine integration grid values (corresponding to Int=ultrafine keyword) were employed for the calculations of **6**:**6**–**8**:**8**, and **10**:**10**, since very low imaginary frequencies were predicted for the frequency analysis under the default condition (fine grid) of Gaussian 09 program.

M06–2X/6–311+G(3d,p)
Compound **1**
Symmetry $D_{6h}$
Energy HF = –232.204406 au
Standard orientation

| | | | | |
|---|---|---|---|---|
| 6 | 0 | 0.000000 | 1.390114 | 0.000000 |
| 6 | 0 | −1.203874 | 0.695057 | 0.000000 |
| 6 | 0 | 1.203874 | 0.695057 | 0.000000 |
| 6 | 0 | −1.203874 | −0.695057 | 0.000000 |
| 6 | 0 | 1.203874 | −0.695057 | 0.000000 |
| 6 | 0 | 0.000000 | −1.390114 | 0.000000 |
| 1 | 0 | 0.000000 | 2.473544 | 0.000000 |

| | | | | |
|---|---|---|---|---|
| 1 | 0 | −2.142152 | 1.236772 | 0.000000 |
| 1 | 0 | 2.142152 | 1.236772 | 0.000000 |
| 1 | 0 | −2.142152 | −1.236772 | 0.000000 |
| 1 | 0 | 2.142152 | −1.236772 | 0.000000 |
| 1 | 0 | 0.000000 | −2.473544 | 0.000000 |

M06–2X/6–311+G(3d,p)
Compound **2**
Symmetry $D_{2h}$
Energy HF = –385.826436 au
Standard orientation

| | | | | |
|---|---|---|---|---|
| 6 | 0 | 0.000000 | 0.000000 | 0.709942 |
| 6 | 0 | 0.000000 | 0.000000 | –0.709942 |
| 6 | 0 | 0.000000 | 1.240125 | 1.396821 |
| 6 | 0 | 0.000000 | 1.240125 | –1.396821 |
| 6 | 0 | 0.000000 | –1.240125 | 1.396821 |
| 6 | 0 | 0.000000 | –1.240125 | –1.396821 |
| 6 | 0 | 0.000000 | 2.421243 | 0.707294 |
| 6 | 0 | 0.000000 | 2.421243 | –0.707294 |
| 6 | 0 | 0.000000 | –2.421243 | 0.707294 |
| 6 | 0 | 0.000000 | –2.421243 | –0.707294 |
| 1 | 0 | 0.000000 | 1.234952 | 2.481438 |
| 1 | 0 | 0.000000 | 1.234952 | –2.481438 |
| 1 | 0 | 0.000000 | –1.234952 | 2.481438 |
| 1 | 0 | 0.000000 | –1.234952 | –2.481438 |
| 1 | 0 | 0.000000 | 3.363088 | 1.242550 |
| 1 | 0 | 0.000000 | 3.363088 | –1.242550 |
| 1 | 0 | 0.000000 | –3.363088 | 1.242550 |
| 1 | 0 | 0.000000 | –3.363088 | –1.242550 |

M06–2X/6–311+G(3d,p)
Compound **3**
Symmetry $C_{2v}$
Energy HF = –539.451928 au
Standard orientation

| | | | | |
|---|---|---|---|---|
| 6 | 0 | 0.000000 | 0.727921 | –0.377300 |
| 6 | 0 | 0.000000 | 1.493001 | –1.561399 |
| 6 | 0 | 0.000000 | 2.868141 | –1.523268 |
| 6 | 0 | 0.000000 | 3.544389 | –0.294189 |
| 6 | 0 | 0.000000 | 2.823866 | 0.874881 |
| 6 | 0 | 0.000000 | 1.414973 | 0.858118 |
| 1 | 0 | 0.000000 | 1.000979 | –2.524660 |
| 1 | 0 | 0.000000 | 3.431123 | –2.448803 |
| 1 | 0 | 0.000000 | 4.627220 | –0.269954 |
| 1 | 0 | 0.000000 | 3.331807 | 1.833262 |
| 6 | 0 | 0.000000 | 0.674717 | 2.088132 |
| 6 | 0 | 0.000000 | –0.674717 | 2.088132 |
| 6 | 0 | 0.000000 | –1.414973 | 0.858118 |
| 6 | 0 | 0.000000 | –0.727921 | –0.377300 |
| 1 | 0 | 0.000000 | 1.228985 | 3.020297 |
| 1 | 0 | 0.000000 | –1.228985 | 3.020297 |
| 6 | 0 | 0.000000 | –2.823866 | 0.874881 |
| 6 | 0 | 0.000000 | –3.544389 | –0.294189 |
| 6 | 0 | 0.000000 | –2.868141 | –1.523268 |

| | | | | |
|---|---|---|---|---|
| 6 | 0 | 0.000000 | –1.493001 | –1.561399 |
| 1 | 0 | 0.000000 | –3.331807 | 1.833262 |
| 1 | 0 | 0.000000 | –4.627220 | –0.269954 |
| 1 | 0 | 0.000000 | –3.431123 | –2.448803 |
| 1 | 0 | 0.000000 | –1.000979 | –2.524660 |

M06–2X/6–311+G(3d,p)
Compound **4**
Symmetry $C_2$
Energy HF = –693.065985 au
Standard orientation

| | | | | |
|---|---|---|---|---|
| 6 | 0 | –0.015680 | 1.283315 | –0.276342 |
| 6 | 0 | –0.412160 | 1.455372 | –1.625191 |
| 6 | 0 | –0.402665 | 2.690742 | –2.225824 |
| 6 | 0 | 0.000000 | 3.830880 | –1.510292 |
| 6 | 0 | 0.308742 | 3.711925 | –0.180083 |
| 6 | 0 | 0.269409 | 2.458449 | 0.467502 |
| 1 | 0 | –0.787633 | 0.610554 | –2.183309 |
| 1 | 0 | –0.729780 | 2.787050 | –3.254148 |
| 1 | 0 | 0.023620 | 4.798937 | –1.995566 |
| 1 | 0 | 0.560533 | 4.588272 | 0.407422 |
| 6 | 0 | 0.433220 | 2.381152 | 1.883471 |
| 6 | 0 | 0.237316 | 1.208399 | 2.528397 |
| 6 | 0 | 0.000000 | 0.000000 | 1.803585 |
| 6 | 0 | 0.000000 | 0.000000 | 0.400898 |
| 1 | 0 | 0.655540 | 3.289862 | 2.431652 |
| 1 | 0 | 0.282034 | 1.156420 | 3.610668 |
| 6 | 0 | –0.237316 | –1.208399 | 2.528397 |
| 6 | 0 | –0.433220 | –2.381152 | 1.883471 |
| 6 | 0 | –0.269409 | –2.458449 | 0.467502 |
| 6 | 0 | 0.015680 | –1.283315 | –0.276342 |
| 1 | 0 | –0.282034 | –1.156420 | 3.610668 |
| 1 | 0 | –0.655540 | –3.289862 | 2.431652 |
| 6 | 0 | –0.308742 | –3.711925 | –0.180083 |
| 6 | 0 | 0.000000 | –3.830880 | –1.510292 |
| 6 | 0 | 0.402665 | –2.690742 | –2.225824 |
| 6 | 0 | 0.412160 | –1.455372 | –1.625191 |
| 1 | 0 | –0.560533 | –4.588272 | 0.407422 |
| 1 | 0 | –0.023620 | –4.798937 | –1.995566 |
| 1 | 0 | 0.729780 | –2.787050 | –3.254148 |
| 1 | 0 | 0.787633 | –0.610554 | –2.183309 |

M06–2X/6–311+G(3d,p)
Compound **5**
Symmetry $C_2$
Energy HF = –846.682614 au
Standard orientation

| | | | | |
|---|---|---|---|---|
| 6 | 0 | –0.185532 | 1.574423 | –0.366176 |
| 6 | 0 | –0.906274 | 1.144073 | –1.504975 |
| 6 | 0 | –1.137851 | 1.983569 | –2.565933 |
| 6 | 0 | –0.662181 | 3.306661 | –2.546680 |
| 6 | 0 | –0.025911 | 3.775623 | –1.427136 |
| 6 | 0 | 0.185532 | 2.940412 | –0.308020 |

| | | | | |
|---|---|---|---|---|
| 1 | 0 | −1.299402 | 0.137590 | −1.534905 |
| 1 | 0 | −1.702532 | 1.625545 | −3.418374 |
| 1 | 0 | −0.832471 | 3.958909 | −3.394597 |
| 1 | 0 | 0.296597 | 4.809518 | −1.368078 |
| 6 | 0 | 0.683111 | 3.490937 | 0.913583 |
| 6 | 0 | 0.684532 | 2.745121 | 2.040790 |
| 6 | 0 | 0.326757 | 1.359817 | 2.012308 |
| 6 | 0 | 0.056644 | 0.720002 | 0.787317 |
| 1 | 0 | 0.980143 | 4.533708 | 0.932714 |
| 1 | 0 | 0.963994 | 3.179804 | 2.994214 |
| 6 | 0 | 0.206424 | 0.646391 | 3.235600 |
| 6 | 0 | −0.206424 | −0.646391 | 3.235600 |
| 6 | 0 | −0.326757 | −1.359817 | 2.012308 |
| 6 | 0 | −0.056644 | −0.720002 | 0.787317 |
| 1 | 0 | 0.390662 | 1.173496 | 4.164956 |
| 1 | 0 | −0.390662 | −1.173496 | 4.164956 |
| 6 | 0 | −0.684532 | −2.745121 | 2.040790 |
| 6 | 0 | −0.683111 | −3.490937 | 0.913583 |
| 6 | 0 | −0.185532 | −2.940412 | −0.308020 |
| 6 | 0 | 0.185532 | −1.574423 | −0.366176 |
| 1 | 0 | −0.963994 | −3.179804 | 2.994214 |
| 1 | 0 | −0.980143 | −4.533708 | 0.932714 |
| 6 | 0 | 0.025911 | −3.775623 | −1.427136 |
| 6 | 0 | 0.662181 | −3.306661 | −2.546680 |
| 6 | 0 | 1.137851 | −1.983569 | −2.565933 |
| 6 | 0 | 0.906274 | −1.144073 | −1.504975 |
| 1 | 0 | −0.296597 | −4.809518 | −1.368078 |
| 1 | 0 | 0.832471 | −3.958909 | −3.394597 |
| 1 | 0 | 1.702532 | −1.625545 | −3.418374 |
| 1 | 0 | 1.299402 | −0.137590 | −1.534905 |

M06–2X/6–311+G(3d,p)
Compound **6**
Symmetry $C_2$
Energy HF = −1000.301653 au
Standard orientation

| | | | | |
|---|---|---|---|---|
| 6 | 0 | −0.391054 | 1.571617 | −0.532161 |
| 6 | 0 | −1.342267 | 0.756225 | −1.186379 |
| 6 | 0 | −1.790887 | 1.053496 | −2.449158 |
| 6 | 0 | −1.312488 | 2.187110 | −3.128276 |
| 6 | 0 | −0.446275 | 3.037458 | −2.491085 |
| 6 | 0 | 0.000000 | 2.769019 | −1.178975 |
| 1 | 0 | −1.742505 | −0.110203 | −0.678710 |
| 1 | 0 | −2.524100 | 0.410418 | −2.920741 |
| 1 | 0 | −1.654294 | 2.404356 | −4.132958 |
| 1 | 0 | −0.109674 | 3.947747 | −2.975521 |
| 6 | 0 | 0.784217 | 3.734179 | −0.472996 |
| 6 | 0 | 1.066206 | 3.554177 | 0.837052 |
| 6 | 0 | 0.707571 | 2.342632 | 1.510141 |
| 6 | 0 | 0.127369 | 1.280936 | 0.794022 |
| 1 | 0 | 1.083331 | 4.639529 | −0.989503 |
| 1 | 0 | 1.579774 | 4.321919 | 1.405255 |
| 6 | 0 | 0.903429 | 2.236677 | 2.915547 |
| 6 | 0 | 0.467648 | 1.138957 | 3.583148 |

| | | | | |
|---|---|---|---|---|
| 6 | 0 | 0.000000 | 0.000000 | 2.869624 |
| 6 | 0 | 0.000000 | 0.000000 | 1.455749 |
| 1 | 0 | 1.335078 | 3.078583 | 3.445125 |
| 1 | 0 | 0.506419 | 1.090934 | 4.665649 |
| 6 | 0 | –0.467648 | –1.138957 | 3.583148 |
| 6 | 0 | –0.903429 | –2.236677 | 2.915547 |
| 6 | 0 | –0.707571 | –2.342632 | 1.510141 |
| 6 | 0 | –0.127369 | –1.280936 | 0.794022 |
| 1 | 0 | –0.506419 | –1.090934 | 4.665649 |
| 1 | 0 | –1.335078 | –3.078583 | 3.445125 |
| 6 | 0 | –1.066206 | –3.554177 | 0.837052 |
| 6 | 0 | –0.784217 | –3.734179 | –0.472996 |
| 6 | 0 | 0.000000 | –2.769019 | –1.178975 |
| 6 | 0 | 0.391054 | –1.571617 | –0.532161 |
| 1 | 0 | –1.579774 | –4.321919 | 1.405255 |
| 1 | 0 | –1.083331 | –4.639529 | –0.989503 |
| 6 | 0 | 0.446275 | –3.037458 | –2.491085 |
| 6 | 0 | 1.312488 | –2.187110 | –3.128276 |
| 6 | 0 | 1.790887 | –1.053496 | –2.449158 |
| 6 | 0 | 1.342267 | –0.756225 | –1.186379 |
| 1 | 0 | 0.109674 | –3.947747 | –2.975521 |
| 1 | 0 | 1.654294 | –2.404356 | –4.132958 |
| 1 | 0 | 2.524100 | –0.410418 | –2.920741 |
| 1 | 0 | 1.742505 | 0.110203 | –0.678710 |

M06–2X/6–311+G(3d,p)
Compound **7**
Symmetry *C*2
Energy HF = –1153.921671 au
Standard orientation

| | | | | |
|---|---|---|---|---|
| 6 | 0 | 1.037792 | 1.079368 | –0.684060 |
| 6 | 0 | –0.110581 | 1.903121 | –0.657945 |
| 6 | 0 | –0.567188 | 2.523614 | –1.794322 |
| 6 | 0 | 0.110581 | 2.361854 | –3.015619 |
| 6 | 0 | 1.278046 | 1.643329 | –3.050123 |
| 6 | 0 | 1.782869 | 1.024885 | –1.885796 |
| 1 | 0 | –0.631867 | 2.061498 | 0.276276 |
| 1 | 0 | –1.451802 | 3.147354 | –1.745922 |
| 1 | 0 | –0.267642 | 2.834878 | –3.913791 |
| 1 | 0 | 1.846686 | 1.559442 | –3.969965 |
| 6 | 0 | 3.072266 | 0.406250 | –1.890941 |
| 6 | 0 | 3.621989 | –0.033949 | –0.735503 |
| 6 | 0 | 2.870030 | –0.040471 | 0.482220 |
| 6 | 0 | 1.524821 | 0.361575 | 0.478638 |
| 1 | 0 | 3.625895 | 0.365862 | –2.822501 |
| 1 | 0 | 4.636695 | –0.416051 | –0.712423 |
| 6 | 0 | 3.487340 | –0.453046 | 1.697804 |
| 6 | 0 | 2.794261 | –0.418913 | 2.864557 |
| 6 | 0 | 1.393013 | –0.163963 | 2.861515 |
| 6 | 0 | 0.722857 | 0.054891 | 1.639858 |
| 1 | 0 | 4.535731 | –0.729903 | 1.680288 |
| 1 | 0 | 3.280868 | –0.629568 | 3.810345 |
| 6 | 0 | 0.665814 | –0.129391 | 4.086676 |
| 6 | 0 | –0.665814 | 0.129391 | 4.086676 |

| | | | | |
|---|---|---|---|---|
| 6 | 0 | −1.393013 | 0.163963 | 2.861515 |
| 6 | 0 | −0.722857 | −0.054891 | 1.639858 |
| 1 | 0 | 1.209752 | −0.256588 | 5.015927 |
| 1 | 0 | −1.209752 | 0.256588 | 5.015927 |
| 6 | 0 | −2.794261 | 0.418913 | 2.864557 |
| 6 | 0 | −3.487340 | 0.453046 | 1.697804 |
| 6 | 0 | −2.870030 | 0.040471 | 0.482220 |
| 6 | 0 | −1.524821 | −0.361575 | 0.478638 |
| 1 | 0 | −3.280868 | 0.629568 | 3.810345 |
| 1 | 0 | −4.535731 | 0.729903 | 1.680288 |
| 6 | 0 | −3.621989 | 0.033949 | −0.735503 |
| 6 | 0 | −3.072266 | −0.406250 | −1.890941 |
| 6 | 0 | −1.782869 | −1.024885 | −1.885796 |
| 6 | 0 | −1.037792 | −1.079368 | −0.684060 |
| 1 | 0 | −4.636695 | 0.416051 | −0.712423 |
| 1 | 0 | −3.625895 | −0.365862 | −2.822501 |
| 6 | 0 | −1.278046 | −1.643329 | −3.050123 |
| 6 | 0 | −0.110581 | −2.361854 | −3.015619 |
| 6 | 0 | 0.567188 | −2.523614 | −1.794322 |
| 6 | 0 | 0.110581 | −1.903121 | −0.657945 |
| 1 | 0 | −1.846686 | −1.559442 | −3.969965 |
| 1 | 0 | 0.267642 | −2.834878 | −3.913791 |
| 1 | 0 | 1.451802 | −3.147354 | −1.745922 |
| 1 | 0 | 0.631867 | −2.061498 | 0.276276 |

M06–2X/6–311+G(3d,p)
Compound **8**
Symmetry $C_2$
Energy HF = −1307.543065 au
Standard orientation

| | | | | |
|---|---|---|---|---|
| 6 | 0 | 0.763200 | 1.424919 | −0.715512 |
| 6 | 0 | 0.000000 | 2.379722 | −0.002862 |
| 6 | 0 | −0.726872 | 3.341472 | −0.657703 |
| 6 | 0 | −0.719358 | 3.406230 | −2.063292 |
| 6 | 0 | 0.081815 | 2.551122 | −2.774655 |
| 6 | 0 | 0.863150 | 1.574707 | −2.118546 |
| 1 | 0 | −0.000227 | 2.360745 | 1.078745 |
| 1 | 0 | −1.305082 | 4.057585 | −0.086131 |
| 1 | 0 | −1.311967 | 4.154521 | −2.575775 |
| 1 | 0 | 0.145471 | 2.624799 | −3.855128 |
| 6 | 0 | 1.805803 | 0.784749 | −2.848884 |
| 6 | 0 | 2.669109 | −0.026612 | −2.194679 |
| 6 | 0 | 2.565051 | −0.225713 | −0.781343 |
| 6 | 0 | 1.513900 | 0.368316 | −0.065976 |
| 1 | 0 | 1.857550 | 0.900863 | −3.926008 |
| 1 | 0 | 3.444517 | −0.562468 | −2.731173 |
| 6 | 0 | 3.534253 | −1.013176 | −0.096430 |
| 6 | 0 | 3.476310 | −1.154522 | 1.253299 |
| 6 | 0 | 2.337865 | −0.695254 | 1.975169 |
| 6 | 0 | 1.274694 | −0.083571 | 1.282636 |
| 1 | 0 | 4.352337 | −1.440269 | −0.665925 |
| 1 | 0 | 4.265385 | −1.663505 | 1.795819 |
| 6 | 0 | 2.253603 | −0.866678 | 3.389438 |
| 6 | 0 | 1.162094 | −0.435727 | 4.071635 |

| | | | | |
|---|---|---|---|---|
| 6 | 0 | 0.000000 | 0.000000 | 3.367865 |
| 6 | 0 | 0.000000 | 0.000000 | 1.961307 |
| 1 | 0 | 3.101972 | –1.297642 | 3.909449 |
| 1 | 0 | 1.130063 | –0.471554 | 5.154844 |
| 6 | 0 | –1.162094 | 0.435727 | 4.071635 |
| 6 | 0 | –2.253603 | 0.866678 | 3.389438 |
| 6 | 0 | –2.337865 | 0.695254 | 1.975169 |
| 6 | 0 | –1.274694 | 0.083571 | 1.282636 |
| 1 | 0 | –1.130063 | 0.471554 | 5.154844 |
| 1 | 0 | –3.101972 | 1.297642 | 3.909449 |
| 6 | 0 | –3.476310 | 1.154522 | 1.253299 |
| 6 | 0 | –3.534253 | 1.013176 | –0.096430 |
| 6 | 0 | –2.565051 | 0.225713 | –0.781343 |
| 6 | 0 | –1.513900 | –0.368316 | –0.065976 |
| 1 | 0 | –4.265385 | 1.663505 | 1.795819 |
| 1 | 0 | –4.352337 | 1.440269 | –0.665925 |
| 6 | 0 | –2.669109 | 0.026612 | –2.194679 |
| 6 | 0 | –1.805803 | –0.784749 | –2.848884 |
| 6 | 0 | –0.863150 | –1.574707 | –2.118546 |
| 6 | 0 | –0.763200 | –1.424919 | –0.715512 |
| 1 | 0 | –3.444517 | 0.562468 | –2.731173 |
| 1 | 0 | –1.857550 | –0.900863 | –3.926008 |
| 6 | 0 | –0.081815 | –2.551122 | –2.774655 |
| 6 | 0 | 0.719358 | –3.406230 | –2.063292 |
| 6 | 0 | 0.726872 | –3.341472 | –0.657703 |
| 6 | 0 | 0.000000 | –2.379722 | –0.002862 |
| 1 | 0 | –0.145471 | –2.624799 | –3.855128 |
| 1 | 0 | 1.311967 | –4.154521 | –2.575775 |
| 1 | 0 | 1.305082 | –4.057585 | –0.086131 |
| 1 | 0 | 0.000227 | –2.360745 | 1.078745 |

M06–2X/6–311+G(3d,p)
Compound **9**
Symmetry $C_2$
Energy HF = –1461.164313 au
Standard orientation

| | | | | |
|---|---|---|---|---|
| 6 | 0 | –2.035651 | –0.170624 | –0.552272 |
| 6 | 0 | –2.628941 | –0.635593 | 0.644939 |
| 6 | 0 | –3.468255 | –1.721949 | 0.649554 |
| 6 | 0 | –3.767728 | –2.396206 | –0.547390 |
| 6 | 0 | –3.279057 | –1.912770 | –1.733885 |
| 6 | 0 | –2.441104 | –0.777041 | –1.764484 |
| 1 | 0 | –2.436737 | –0.115235 | 1.573225 |
| 1 | 0 | –3.906131 | –2.055845 | 1.582737 |
| 1 | 0 | –4.413256 | –3.266296 | –0.534921 |
| 1 | 0 | –3.547857 | –2.383096 | –2.673781 |
| 6 | 0 | –2.061035 | –0.188655 | –3.012058 |
| 6 | 0 | –1.404076 | 0.993428 | –3.037983 |
| 6 | 0 | –0.944875 | 1.609339 | –1.830264 |
| 6 | 0 | –1.118912 | 0.955063 | –0.600460 |
| 1 | 0 | –2.371075 | –0.674218 | –3.931171 |
| 1 | 0 | –1.185983 | 1.487648 | –3.978489 |
| 6 | 0 | –0.326916 | 2.892006 | –1.873288 |
| 6 | 0 | 0.038895 | 3.519405 | –0.725667 |

| | | | | |
|---|---|---|---|---|
| 6 | 0 | –0.007112 | 2.826116 | 0.516984 |
| 6 | 0 | –0.424747 | 1.482258 | 0.548809 |
| 1 | 0 | –0.213700 | 3.380817 | –2.834919 |
| 1 | 0 | 0.419555 | 4.534519 | –0.740204 |
| 6 | 0 | 0.375766 | 3.474627 | 1.728948 |
| 6 | 0 | 0.326916 | 2.811382 | 2.913023 |
| 6 | 0 | 0.105756 | 1.402401 | 2.936177 |
| 6 | 0 | –0.091405 | 0.716016 | 1.726818 |
| 1 | 0 | 0.652124 | 4.522543 | 1.688972 |
| 1 | 0 | 0.519481 | 3.323588 | 3.849209 |
| 6 | 0 | 0.102218 | 0.671459 | 4.163428 |
| 6 | 0 | –0.102218 | –0.671459 | 4.163428 |
| 6 | 0 | –0.105756 | –1.402401 | 2.936177 |
| 6 | 0 | 0.091405 | –0.716016 | 1.726818 |
| 1 | 0 | 0.207737 | 1.218480 | 5.093770 |
| 1 | 0 | –0.207737 | –1.218480 | 5.093770 |
| 6 | 0 | –0.326916 | –2.811382 | 2.913023 |
| 6 | 0 | –0.375766 | –3.474627 | 1.728948 |
| 6 | 0 | 0.007112 | –2.826116 | 0.516984 |
| 6 | 0 | 0.424747 | –1.482258 | 0.548809 |
| 1 | 0 | –0.519481 | –3.323588 | 3.849209 |
| 1 | 0 | –0.652124 | –4.522543 | 1.688972 |
| 6 | 0 | –0.038895 | –3.519405 | –0.725667 |
| 6 | 0 | 0.326916 | –2.892006 | –1.873288 |
| 6 | 0 | 0.944875 | –1.609339 | –1.830264 |
| 6 | 0 | 1.118912 | –0.955063 | –0.600460 |
| 1 | 0 | –0.419555 | –4.534519 | –0.740204 |
| 1 | 0 | 0.213700 | –3.380817 | –2.834919 |
| 6 | 0 | 1.404076 | –0.993428 | –3.037983 |
| 6 | 0 | 2.061035 | 0.188655 | –3.012058 |
| 6 | 0 | 2.441104 | 0.777041 | –1.764484 |
| 6 | 0 | 2.035651 | 0.170624 | –0.552272 |
| 1 | 0 | 1.185983 | –1.487648 | –3.978489 |
| 1 | 0 | 2.371075 | 0.674218 | –3.931171 |
| 6 | 0 | 3.279057 | 1.912770 | –1.733885 |
| 6 | 0 | 3.767728 | 2.396206 | –0.547390 |
| 6 | 0 | 3.468255 | 1.721949 | 0.649554 |
| 6 | 0 | 2.628941 | 0.635593 | 0.644939 |
| 1 | 0 | 3.547857 | 2.383096 | –2.673781 |
| 1 | 0 | 4.413256 | 3.266296 | –0.534921 |
| 1 | 0 | 3.906131 | 2.055845 | 1.582737 |
| 1 | 0 | 2.436737 | 0.115235 | 1.573225 |

M06–2X/6–311+G(3d,p)
Compound **10**
Symmetry $C_2$
Energy HF = –1614.784600 au
Standard orientation

| | | | | |
|---|---|---|---|---|
| 6 | 0 | 0.934737 | 2.325724 | –0.204510 |
| 6 | 0 | 1.312885 | 2.529175 | 1.143743 |
| 6 | 0 | 0.997005 | 3.688823 | 1.806399 |
| 6 | 0 | 0.293241 | 4.715322 | 1.150807 |
| 6 | 0 | 0.000000 | 4.585432 | –0.182142 |
| 6 | 0 | 0.349901 | 3.415667 | –0.891681 |

| | | | | |
|---|---|---|---|---|
| 1 | 0 | 1.881203 | 1.766872 | 1.658406 |
| 1 | 0 | 1.299803 | 3.814042 | 2.839107 |
| 1 | 0 | 0.026251 | 5.618892 | 1.685701 |
| 1 | 0 | –0.484232 | 5.392571 | –0.721734 |
| 6 | 0 | 0.199363 | 3.356863 | –2.312933 |
| 6 | 0 | 0.702912 | 2.311289 | –3.008087 |
| 6 | 0 | 1.252203 | 1.173368 | –2.335688 |
| 6 | 0 | 1.220462 | 1.102516 | –0.934355 |
| 1 | 0 | –0.258471 | 4.197878 | –2.822553 |
| 1 | 0 | 0.675337 | 2.299933 | –4.092355 |
| 6 | 0 | 1.847826 | 0.120884 | –3.088958 |
| 6 | 0 | 2.434786 | –0.928866 | –2.460114 |
| 6 | 0 | 2.296995 | –1.093177 | –1.052183 |
| 6 | 0 | 1.546659 | –0.159883 | –0.311419 |
| 1 | 0 | 1.876337 | 0.209486 | –4.169697 |
| 1 | 0 | 2.970294 | –1.687675 | –3.020021 |
| 6 | 0 | 2.910229 | –2.196473 | –0.386639 |
| 6 | 0 | 2.802376 | –2.339613 | 0.959154 |
| 6 | 0 | 1.908528 | –1.510900 | 1.699194 |
| 6 | 0 | 1.148417 | –0.543090 | 1.023474 |
| 1 | 0 | 3.506666 | –2.886126 | –0.973893 |
| 1 | 0 | 3.340639 | –3.121838 | 1.482862 |
| 6 | 0 | 1.764337 | –1.651882 | 3.113249 |
| 6 | 0 | 0.917507 | –0.843905 | 3.803445 |
| 6 | 0 | 0.000000 | 0.000000 | 3.107045 |
| 6 | 0 | 0.000000 | 0.000000 | 1.704639 |
| 1 | 0 | 2.387651 | –2.374766 | 3.627765 |
| 1 | 0 | 0.878626 | –0.872534 | 4.886818 |
| 6 | 0 | –0.917507 | 0.843905 | 3.803445 |
| 6 | 0 | –1.764337 | 1.651882 | 3.113249 |
| 6 | 0 | –1.908528 | 1.510900 | 1.699194 |
| 6 | 0 | –1.148417 | 0.543090 | 1.023474 |
| 1 | 0 | –0.878626 | 0.872534 | 4.886818 |
| 1 | 0 | –2.387651 | 2.374766 | 3.627765 |
| 6 | 0 | –2.802376 | 2.339613 | 0.959154 |
| 6 | 0 | –2.910229 | 2.196473 | –0.386639 |
| 6 | 0 | –2.296995 | 1.093177 | –1.052183 |
| 6 | 0 | –1.546659 | 0.159883 | –0.311419 |
| 1 | 0 | –3.340639 | 3.121838 | 1.482862 |
| 1 | 0 | –3.506666 | 2.886126 | –0.973893 |
| 6 | 0 | –2.434786 | 0.928866 | –2.460114 |
| 6 | 0 | –1.847826 | –0.120884 | –3.088958 |
| 6 | 0 | –1.252203 | –1.173368 | –2.335688 |
| 6 | 0 | –1.220462 | –1.102516 | –0.934355 |
| 1 | 0 | –2.970294 | 1.687675 | –3.020021 |
| 1 | 0 | –1.876337 | –0.209486 | –4.169697 |
| 6 | 0 | –0.702912 | –2.311289 | –3.008087 |
| 6 | 0 | –0.199363 | –3.356863 | –2.312933 |
| 6 | 0 | –0.349901 | –3.415667 | –0.891681 |
| 6 | 0 | –0.934737 | –2.325724 | –0.204510 |
| 1 | 0 | –0.675337 | –2.299933 | –4.092355 |
| 1 | 0 | 0.258471 | –4.197878 | –2.822553 |
| 6 | 0 | 0.000000 | –4.585432 | –0.182142 |
| 6 | 0 | –0.293241 | –4.715322 | 1.150807 |
| 6 | 0 | –0.997005 | –3.688823 | 1.806399 |

| | | | | |
|---|---|---|---|---|
| 6 | 0 | −1.312885 | −2.529175 | 1.143743 |
| 1 | 0 | 0.484232 | −5.392571 | −0.721734 |
| 1 | 0 | −0.026251 | −5.618892 | 1.685701 |
| 1 | 0 | −1.299803 | −3.814042 | 2.839107 |
| 1 | 0 | −1.881203 | −1.766872 | 1.658406 |

M06–2X/6–311+G(3d,p)
Compound **11**
Symmetry $C_2$
Energy HF = −1768.405064 au
Standard orientation

| | | | | |
|---|---|---|---|---|
| 6 | 0 | 2.755719 | −0.589388 | −0.251277 |
| 6 | 0 | 2.930875 | 0.261511 | −1.367941 |
| 6 | 0 | 3.566655 | −0.173819 | −2.503611 |
| 6 | 0 | 4.074268 | −1.483233 | −2.577882 |
| 6 | 0 | 4.003476 | −2.297154 | −1.476567 |
| 6 | 0 | 3.384124 | −1.855966 | −0.286690 |
| 1 | 0 | 2.574951 | 1.281429 | −1.320835 |
| 1 | 0 | 3.682948 | 0.501203 | −3.343046 |
| 1 | 0 | 4.556187 | −1.829069 | −3.484578 |
| 1 | 0 | 4.446793 | −3.287176 | −1.493043 |
| 6 | 0 | 3.464969 | −2.638141 | 0.908930 |
| 6 | 0 | 3.041470 | −2.125867 | 2.086969 |
| 6 | 0 | 2.362787 | −0.866406 | 2.144184 |
| 6 | 0 | 2.066809 | −0.175093 | 0.958802 |
| 1 | 0 | 3.941287 | −3.611594 | 0.862155 |
| 1 | 0 | 3.184693 | −2.670898 | 3.013871 |
| 6 | 0 | 2.013815 | −0.299971 | 3.403732 |
| 6 | 0 | 1.447310 | 0.932036 | 3.469206 |
| 6 | 0 | 1.010358 | 1.588105 | 2.283277 |
| 6 | 0 | 1.167679 | 0.953398 | 1.036461 |
| 1 | 0 | 2.271692 | −0.843441 | 4.306418 |
| 1 | 0 | 1.266844 | 1.413637 | 4.424060 |
| 6 | 0 | 0.413033 | 2.883015 | 2.347303 |
| 6 | 0 | 0.016031 | 3.518982 | 1.215878 |
| 6 | 0 | −0.001761 | 2.825179 | −0.029849 |
| 6 | 0 | 0.412238 | 1.484035 | −0.077347 |
| 1 | 0 | 0.333308 | 3.366939 | 3.314908 |
| 1 | 0 | −0.355758 | 4.537130 | 1.248662 |
| 6 | 0 | −0.432246 | 3.468096 | −1.230683 |
| 6 | 0 | −0.412238 | 2.804021 | −2.415968 |
| 6 | 0 | −0.166258 | 1.397814 | −2.449404 |
| 6 | 0 | 0.059105 | 0.716825 | −1.244737 |
| 1 | 0 | −0.720945 | 4.512555 | −1.183386 |
| 1 | 0 | −0.644259 | 3.313908 | −3.344404 |
| 6 | 0 | −0.134947 | 0.666457 | −3.675242 |
| 6 | 0 | 0.134947 | −0.666457 | −3.675242 |
| 6 | 0 | 0.166258 | −1.397814 | −2.449404 |
| 6 | 0 | −0.059105 | −0.716825 | −1.244737 |
| 1 | 0 | −0.269274 | 1.206515 | −4.606041 |
| 1 | 0 | 0.269274 | −1.206515 | −4.606041 |
| 6 | 0 | 0.412238 | −2.804021 | −2.415968 |
| 6 | 0 | 0.432246 | −3.468096 | −1.230683 |
| 6 | 0 | 0.001761 | −2.825179 | −0.029849 |

| | | | | |
|---|---|---|---|---|
| 6 | 0 | −0.412238 | −1.484035 | −0.077347 |
| 1 | 0 | 0.644259 | −3.313908 | −3.344404 |
| 1 | 0 | 0.720945 | −4.512555 | −1.183386 |
| 6 | 0 | −0.016031 | −3.518982 | 1.215878 |
| 6 | 0 | −0.413033 | −2.883015 | 2.347303 |
| 6 | 0 | −1.010358 | −1.588105 | 2.283277 |
| 6 | 0 | −1.167679 | −0.953398 | 1.036461 |
| 1 | 0 | 0.355758 | −4.537130 | 1.248662 |
| 1 | 0 | −0.333308 | −3.366939 | 3.314908 |
| 6 | 0 | −1.447310 | −0.932036 | 3.469206 |
| 6 | 0 | −2.013815 | 0.299971 | 3.403732 |
| 6 | 0 | −2.362787 | 0.866406 | 2.144184 |
| 6 | 0 | −2.066809 | 0.175093 | 0.958802 |
| 1 | 0 | −1.266844 | −1.413637 | 4.424060 |
| 1 | 0 | −2.271692 | 0.843441 | 4.306418 |
| 6 | 0 | −3.041470 | 2.125867 | 2.086969 |
| 6 | 0 | −3.464969 | 2.638141 | 0.908930 |
| 1 | 0 | −3.184693 | 2.670898 | 3.013871 |
| 1 | 0 | −3.941287 | 3.611594 | 0.862155 |
| 6 | 0 | −3.384124 | 1.855966 | −0.286690 |
| 6 | 0 | −2.755719 | 0.589388 | −0.251277 |
| 6 | 0 | −4.003476 | 2.297154 | −1.476567 |
| 6 | 0 | −4.074268 | 1.483233 | −2.577882 |
| 6 | 0 | −3.566655 | 0.173819 | −2.503611 |
| 6 | 0 | −2.930875 | −0.261511 | −1.367941 |
| 1 | 0 | −4.446793 | 3.287176 | −1.493043 |
| 1 | 0 | −4.556187 | 1.829069 | −3.484578 |
| 1 | 0 | −3.682948 | −0.501203 | −3.343046 |
| 1 | 0 | −2.574951 | −1.281429 | −1.320835 |

M06–2X/6–311+G(3d,p)
Compound **12**
Symmetry $C_2$
Energy HF = −1922.025696 au
Standard orientation

| | | | | |
|---|---|---|---|---|
| 6 | 0 | −2.901953 | −0.548724 | 0.674131 |
| 6 | 0 | −3.053865 | 0.727883 | 1.264826 |
| 6 | 0 | −3.503986 | 0.868889 | 2.553972 |
| 6 | 0 | −3.839635 | −0.262289 | 3.319221 |
| 6 | 0 | −3.794367 | −1.504428 | 2.740589 |
| 6 | 0 | −3.366573 | −1.667767 | 1.404933 |
| 1 | 0 | −2.828346 | 1.612978 | 0.686212 |
| 1 | 0 | −3.605877 | 1.860171 | 2.979425 |
| 1 | 0 | −4.173660 | −0.146613 | 4.343353 |
| 1 | 0 | −4.109918 | −2.382870 | 3.293654 |
| 6 | 0 | −3.479777 | −2.938069 | 0.757009 |
| 6 | 0 | −3.248043 | −3.047359 | −0.571238 |
| 6 | 0 | −2.737197 | −1.943214 | −1.325679 |
| 6 | 0 | −2.402438 | −0.744434 | −0.676252 |
| 1 | 0 | −3.825865 | −3.788262 | 1.335036 |
| 1 | 0 | −3.420624 | −3.984293 | −1.089975 |
| 6 | 0 | −2.595224 | −2.051351 | −2.739182 |
| 6 | 0 | −2.197477 | −0.980563 | −3.472154 |
| 6 | 0 | −1.725770 | 0.198896 | −2.828228 |

| | | | | |
|---|---|---|---|---|
| 6 | 0 | −1.661961 | 0.248474 | −1.421914 |
| 1 | 0 | −2.877843 | −2.981811 | −3.219816 |
| 1 | 0 | −2.182940 | −1.019695 | −4.555860 |
| 6 | 0 | −1.322515 | 1.332639 | −3.594804 |
| 6 | 0 | −0.904858 | 2.468038 | −2.979483 |
| 6 | 0 | −0.660065 | 2.476124 | −1.574922 |
| 6 | 0 | −0.868288 | 1.303877 | −0.830303 |
| 1 | 0 | −1.413011 | 1.284605 | −4.674731 |
| 1 | 0 | −0.685413 | 3.364447 | −3.549115 |
| 6 | 0 | −0.206833 | 3.657392 | −0.911824 |
| 6 | 0 | 0.000000 | 3.659092 | 0.430148 |
| 6 | 0 | −0.028801 | 2.436452 | 1.166590 |
| 6 | 0 | −0.288518 | 1.235100 | 0.490043 |
| 1 | 0 | −0.087280 | 4.564417 | −1.494702 |
| 1 | 0 | 0.254940 | 4.573912 | 0.953777 |
| 6 | 0 | 0.182670 | 2.411043 | 2.578348 |
| 6 | 0 | 0.090981 | 1.243110 | 3.266990 |
| 6 | 0 | 0.000000 | 0.000000 | 2.571543 |
| 6 | 0 | 0.000000 | 0.000000 | 1.170006 |
| 1 | 0 | 0.357718 | 3.349364 | 3.093485 |
| 1 | 0 | 0.149488 | 1.228709 | 4.349712 |
| 6 | 0 | −0.090981 | −1.243110 | 3.266990 |
| 6 | 0 | −0.182670 | −2.411043 | 2.578348 |
| 6 | 0 | 0.028801 | −2.436452 | 1.166590 |
| 6 | 0 | 0.288518 | −1.235100 | 0.490043 |
| 1 | 0 | −0.149488 | −1.228709 | 4.349712 |
| 1 | 0 | −0.357718 | −3.349364 | 3.093485 |
| 6 | 0 | 0.000000 | −3.659092 | 0.430148 |
| 6 | 0 | 0.206833 | −3.657392 | −0.911824 |
| 6 | 0 | 0.660065 | −2.476124 | −1.574922 |
| 6 | 0 | 0.868288 | −1.303877 | −0.830303 |
| 1 | 0 | −0.254940 | −4.573912 | 0.953777 |
| 1 | 0 | 0.087280 | −4.564417 | −1.494702 |
| 6 | 0 | 0.904858 | −2.468038 | −2.979483 |
| 6 | 0 | 1.322515 | −1.332639 | −3.594804 |
| 6 | 0 | 1.725770 | −0.198896 | −2.828228 |
| 6 | 0 | 1.661961 | −0.248474 | −1.421914 |
| 1 | 0 | 0.685413 | −3.364447 | −3.549115 |
| 1 | 0 | 1.413011 | −1.284605 | −4.674731 |
| 6 | 0 | 2.197477 | 0.980563 | −3.472154 |
| 6 | 0 | 2.595224 | 2.051351 | −2.739182 |
| 6 | 0 | 2.737197 | 1.943214 | −1.325679 |
| 6 | 0 | 2.402438 | 0.744434 | −0.676252 |
| 1 | 0 | 2.182940 | 1.019695 | −4.555860 |
| 1 | 0 | 2.877843 | 2.981811 | −3.219816 |
| 6 | 0 | 3.248043 | 3.047359 | −0.571238 |
| 6 | 0 | 3.479777 | 2.938069 | 0.757009 |
| 6 | 0 | 3.366573 | 1.667767 | 1.404933 |
| 6 | 0 | 2.901953 | 0.548724 | 0.674131 |
| 1 | 0 | 3.420624 | 3.984293 | −1.089975 |
| 1 | 0 | 3.825865 | 3.788262 | 1.335036 |
| 6 | 0 | 3.794367 | 1.504428 | 2.740589 |
| 6 | 0 | 3.839635 | 0.262289 | 3.319221 |
| 6 | 0 | 3.503986 | −0.868889 | 2.553972 |
| 6 | 0 | 3.053865 | −0.727883 | 1.264826 |

| | | | | |
|---|---|---|---|---|
| 1 | 0 | 4.109918 | 2.382870 | 3.293654 |
| 1 | 0 | 4.173660 | 0.146613 | 4.343353 |
| 1 | 0 | 3.605877 | –1.860171 | 2.979425 |
| 1 | 0 | 2.828346 | –1.612978 | 0.686212 |

M06–2X/6–311+G(3d,p) with the option of ultrafine grid
Compound **6**:**6**
Symmetry $C_i$
Energy HF = –2000.613340 au
Standard orientation

| | | | | |
|---|---|---|---|---|
| 6 | 0 | –4.787632 | 1.320917 | –0.422435 |
| 6 | 0 | –3.556568 | 1.264922 | –1.116519 |
| 6 | 0 | –2.887321 | 2.408189 | –1.477634 |
| 6 | 0 | –3.402535 | 3.673578 | –1.144926 |
| 6 | 0 | –4.620631 | 3.761074 | –0.522668 |
| 6 | 0 | –5.350870 | 2.599076 | –0.189762 |
| 1 | 0 | –3.150499 | 0.302438 | –1.398367 |
| 1 | 0 | –1.963064 | 2.337462 | –2.039430 |
| 1 | 0 | –2.857704 | 4.570154 | –1.414434 |
| 1 | 0 | –5.061639 | 4.728226 | –0.307261 |
| 6 | 0 | –6.688749 | 2.706812 | 0.304134 |
| 6 | 0 | –7.454908 | 1.600492 | 0.436091 |
| 6 | 0 | –6.910377 | 0.294552 | 0.218980 |
| 6 | 0 | –5.538030 | 0.136134 | –0.041857 |
| 1 | 0 | –7.095350 | 3.693593 | 0.495198 |
| 1 | 0 | –8.499786 | 1.678226 | 0.715438 |
| 6 | 0 | –7.773513 | –0.837167 | 0.223944 |
| 6 | 0 | –7.288948 | –2.063754 | –0.094577 |
| 6 | 0 | –5.887520 | –2.272567 | –0.229829 |
| 6 | 0 | –4.988956 | –1.201928 | –0.018003 |
| 1 | 0 | –8.830913 | –0.684060 | 0.407537 |
| 1 | 0 | –7.951563 | –2.914280 | –0.208470 |
| 6 | 0 | –5.390823 | –3.560614 | –0.574551 |
| 6 | 0 | –4.055314 | –3.774669 | –0.684400 |
| 6 | 0 | –3.133332 | –2.778289 | –0.257391 |
| 6 | 0 | –3.599859 | –1.535877 | 0.207609 |
| 1 | 0 | –6.102970 | –4.350501 | –0.785058 |
| 1 | 0 | –3.668846 | –4.730497 | –1.019723 |
| 6 | 0 | –1.729900 | –3.065271 | –0.262419 |
| 6 | 0 | –0.837791 | –2.196475 | 0.263854 |
| 6 | 0 | –1.296114 | –1.031346 | 0.956352 |
| 6 | 0 | –2.676932 | –0.718437 | 0.978061 |
| 1 | 0 | –1.400970 | –3.996312 | –0.711002 |
| 1 | 0 | 0.229463 | –2.386211 | 0.215365 |
| 6 | 0 | –0.380676 | –0.222043 | 1.662962 |
| 6 | 0 | –0.813559 | 0.824704 | 2.434839 |
| 6 | 0 | –2.192097 | 1.076527 | 2.543823 |
| 6 | 0 | –3.096668 | 0.326497 | 1.833482 |
| 1 | 0 | 0.677145 | –0.446669 | 1.577286 |
| 1 | 0 | –0.100850 | 1.441238 | 2.969206 |
| 1 | 0 | –2.547804 | 1.864363 | 3.196780 |
| 1 | 0 | –4.151638 | 0.529311 | 1.952250 |
| 6 | 0 | 2.676932 | 0.718437 | –0.978061 |
| 6 | 0 | 3.096668 | –0.326497 | –1.833482 |

| | | | | |
|---|---|---|---|---|
| 6 | 0 | 2.192097 | –1.076527 | –2.543823 |
| 6 | 0 | 0.813559 | –0.824704 | –2.434839 |
| 6 | 0 | 0.380676 | 0.222043 | –1.662962 |
| 6 | 0 | 1.296114 | 1.031346 | –0.956352 |
| 1 | 0 | 4.151638 | –0.529311 | –1.952250 |
| 1 | 0 | 2.547804 | –1.864363 | –3.196780 |
| 1 | 0 | 0.100850 | –1.441238 | –2.969206 |
| 1 | 0 | –0.677145 | 0.446669 | –1.577286 |
| 6 | 0 | 0.837791 | 2.196475 | –0.263854 |
| 6 | 0 | 1.729900 | 3.065271 | 0.262419 |
| 6 | 0 | 3.133332 | 2.778289 | 0.257391 |
| 6 | 0 | 3.599859 | 1.535877 | –0.207609 |
| 1 | 0 | –0.229463 | 2.386211 | –0.215365 |
| 1 | 0 | 1.400970 | 3.996312 | 0.711002 |
| 6 | 0 | 4.055314 | 3.774669 | 0.684400 |
| 6 | 0 | 5.390823 | 3.560614 | 0.574551 |
| 6 | 0 | 5.887520 | 2.272567 | 0.229829 |
| 6 | 0 | 4.988956 | 1.201928 | 0.018003 |
| 1 | 0 | 3.668846 | 4.730497 | 1.019723 |
| 1 | 0 | 6.102970 | 4.350501 | 0.785058 |
| 6 | 0 | 7.288948 | 2.063754 | 0.094577 |
| 6 | 0 | 7.773513 | 0.837167 | –0.223944 |
| 6 | 0 | 6.910377 | –0.294552 | –0.218980 |
| 6 | 0 | 5.538030 | –0.136134 | 0.041857 |
| 1 | 0 | 7.951563 | 2.914280 | 0.208470 |
| 1 | 0 | 8.830913 | 0.684060 | –0.407537 |
| 6 | 0 | 7.454908 | –1.600492 | –0.436091 |
| 6 | 0 | 6.688749 | –2.706812 | –0.304134 |
| 6 | 0 | 5.350870 | –2.599076 | 0.189762 |
| 6 | 0 | 4.787632 | –1.320917 | 0.422435 |
| 1 | 0 | 8.499786 | –1.678226 | –0.715438 |
| 1 | 0 | 7.095350 | –3.693593 | –0.495198 |
| 6 | 0 | 4.620631 | –3.761074 | 0.522668 |
| 6 | 0 | 3.402535 | –3.673578 | 1.144926 |
| 6 | 0 | 2.887321 | –2.408189 | 1.477634 |
| 6 | 0 | 3.556568 | –1.264922 | 1.116519 |
| 1 | 0 | 5.061639 | –4.728226 | 0.307261 |
| 1 | 0 | 2.857704 | –4.570154 | 1.414434 |
| 1 | 0 | 1.963064 | –2.337462 | 2.039430 |
| 1 | 0 | 3.150499 | –0.302438 | 1.398367 |

M06–2X/6–311+G(3d,p) with the option of ultrafine grid
Compound **7:7**
Symmetry $C_i$
Energy HF = –2307.855458 au
Standard orientation

| | | | | |
|---|---|---|---|---|
| 6 | 0 | 3.794660 | –0.321732 | 1.797240 |
| 1 | 0 | 4.859027 | –0.372265 | 1.980816 |
| 6 | 0 | 2.961978 | –1.211399 | 2.430553 |
| 1 | 0 | 3.382618 | –1.961131 | 3.089934 |
| 6 | 0 | 1.570886 | –1.151405 | 2.230298 |
| 1 | 0 | 0.918336 | –1.876729 | 2.702715 |
| 6 | 0 | 1.041187 | –0.137082 | 1.473637 |
| 1 | 0 | –0.032317 | –0.033948 | 1.356936 |

| | | | | |
|---|---|---|---|---|
| 6 | 0 | 1.879221 | 0.812898 | 0.849362 |
| 6 | 0 | 1.315697 | 1.958917 | 0.203492 |
| 1 | 0 | 0.235401 | 2.055270 | 0.171518 |
| 6 | 0 | 2.124010 | 2.931881 | –0.277551 |
| 1 | 0 | 1.709591 | 3.846419 | –0.687941 |
| 6 | 0 | 3.546694 | 2.771918 | –0.287966 |
| 6 | 0 | 4.373627 | 3.832691 | –0.755976 |
| 1 | 0 | 3.902701 | 4.753801 | –1.080572 |
| 6 | 0 | 5.725054 | 3.716355 | –0.716011 |
| 1 | 0 | 6.366174 | 4.552351 | –0.971972 |
| 6 | 0 | 6.331242 | 2.470156 | –0.388971 |
| 6 | 0 | 7.751072 | 2.360215 | –0.336541 |
| 1 | 0 | 8.345910 | 3.249175 | –0.513337 |
| 6 | 0 | 8.334309 | 1.184018 | 0.001917 |
| 1 | 0 | 9.406565 | 1.111708 | 0.144683 |
| 6 | 0 | 7.553027 | –0.003366 | 0.104221 |
| 6 | 0 | 8.174385 | –1.237692 | 0.448872 |
| 1 | 0 | 9.234731 | –1.238205 | 0.674804 |
| 6 | 0 | 7.436596 | –2.372078 | 0.547761 |
| 1 | 0 | 7.881503 | –3.299137 | 0.891449 |
| 6 | 0 | 6.079398 | –2.385453 | 0.115637 |
| 6 | 0 | 5.327163 | –3.601309 | 0.173261 |
| 1 | 0 | 5.794840 | –4.471431 | 0.620720 |
| 6 | 0 | 4.058478 | –3.658324 | –0.292890 |
| 1 | 0 | 3.474184 | –4.568040 | –0.210741 |
| 6 | 0 | 3.500455 | –2.545577 | –0.996175 |
| 6 | 0 | 2.256984 | –2.668663 | –1.653807 |
| 1 | 0 | 1.696770 | –3.590363 | –1.536137 |
| 6 | 0 | 1.783293 | –1.664967 | –2.458618 |
| 1 | 0 | 0.830030 | –1.770860 | –2.963442 |
| 6 | 0 | 2.561760 | –0.509326 | –2.653348 |
| 1 | 0 | 2.221314 | 0.261366 | –3.335148 |
| 6 | 0 | 3.757440 | –0.355190 | –1.995804 |
| 1 | 0 | 4.352816 | 0.527767 | –2.183597 |
| 6 | 0 | 4.236578 | –1.341395 | –1.102599 |
| 6 | 0 | 5.482453 | –1.210193 | –0.370144 |
| 6 | 0 | 6.162915 | 0.042977 | –0.134286 |
| 6 | 0 | 5.524434 | 1.345861 | –0.111191 |
| 6 | 0 | 4.126585 | 1.577525 | 0.172331 |
| 6 | 0 | 3.284258 | 0.666702 | 0.924581 |
| 6 | 0 | –3.794660 | 0.321732 | –1.797240 |
| 1 | 0 | –4.859027 | 0.372265 | –1.980816 |
| 6 | 0 | –2.961978 | 1.211399 | –2.430553 |
| 1 | 0 | –3.382618 | 1.961131 | –3.089934 |
| 6 | 0 | –1.570886 | 1.151405 | –2.230298 |
| 1 | 0 | –0.918336 | 1.876729 | –2.702715 |
| 6 | 0 | –1.041187 | 0.137082 | –1.473637 |
| 1 | 0 | 0.032317 | 0.033948 | –1.356936 |
| 6 | 0 | –1.879221 | –0.812898 | –0.849362 |
| 6 | 0 | –1.315697 | –1.958917 | –0.203492 |
| 1 | 0 | –0.235401 | –2.055270 | –0.171518 |
| 6 | 0 | –2.124010 | –2.931881 | 0.277551 |
| 1 | 0 | –1.709591 | –3.846419 | 0.687941 |
| 6 | 0 | –3.546694 | –2.771918 | 0.287966 |
| 6 | 0 | –4.373627 | –3.832691 | 0.755976 |

| | | | | |
|---|---|---|---|---|
| 1 | 0 | –3.902701 | –4.753801 | 1.080572 |
| 6 | 0 | –5.725054 | –3.716355 | 0.716011 |
| 1 | 0 | –6.366174 | –4.552351 | 0.971972 |
| 6 | 0 | –6.331242 | –2.470156 | 0.388971 |
| 6 | 0 | –7.751072 | –2.360215 | 0.336541 |
| 1 | 0 | –8.345910 | –3.249175 | 0.513337 |
| 6 | 0 | –8.334309 | –1.184018 | –0.001917 |
| 1 | 0 | –9.406565 | –1.111708 | –0.144683 |
| 6 | 0 | –7.553027 | 0.003366 | –0.104221 |
| 6 | 0 | –8.174385 | 1.237692 | –0.448872 |
| 1 | 0 | –9.234731 | 1.238205 | –0.674804 |
| 6 | 0 | –7.436596 | 2.372078 | –0.547761 |
| 1 | 0 | –7.881503 | 3.299137 | –0.891449 |
| 6 | 0 | –6.079398 | 2.385453 | –0.115637 |
| 6 | 0 | –5.327163 | 3.601309 | –0.173261 |
| 1 | 0 | –5.794840 | 4.471431 | –0.620720 |
| 6 | 0 | –4.058478 | 3.658324 | 0.292890 |
| 1 | 0 | –3.474184 | 4.568040 | 0.210741 |
| 6 | 0 | –3.500455 | 2.545577 | 0.996175 |
| 6 | 0 | –2.256984 | 2.668663 | 1.653807 |
| 1 | 0 | –1.696770 | 3.590363 | 1.536137 |
| 6 | 0 | –1.783293 | 1.664967 | 2.458618 |
| 1 | 0 | –0.830030 | 1.770860 | 2.963442 |
| 6 | 0 | –2.561760 | 0.509326 | 2.653348 |
| 1 | 0 | –2.221314 | –0.261366 | 3.335148 |
| 6 | 0 | –3.757440 | 0.355190 | 1.995804 |
| 1 | 0 | –4.352816 | –0.527767 | 2.183597 |
| 6 | 0 | –4.236578 | 1.341395 | 1.102599 |
| 6 | 0 | –5.482453 | 1.210193 | 0.370144 |
| 6 | 0 | –6.162915 | –0.042977 | 0.134286 |
| 6 | 0 | –5.524434 | –1.345861 | 0.111191 |
| 6 | 0 | –4.126585 | –1.577525 | –0.172331 |
| 6 | 0 | –3.284258 | –0.666702 | –0.924581 |

M06–2X/6–311+G(2d,p)
Compound **7**
Symmetry $C_2$
Energy HF = –1153.926722 au
Standard orientation

| | | | | |
|---|---|---|---|---|
| 6 | 0 | 1.037135 | 1.085382 | –0.685293 |
| 6 | 0 | –0.114555 | 1.902900 | –0.660902 |
| 6 | 0 | –0.569058 | 2.525838 | –1.795719 |
| 6 | 0 | 0.114542 | 2.373754 | –3.013818 |
| 6 | 0 | 1.284317 | 1.660878 | –3.046532 |
| 6 | 0 | 1.786427 | 1.039213 | –1.883924 |
| 1 | 0 | –0.639335 | 2.056532 | 0.271501 |
| 1 | 0 | –1.455918 | 3.145469 | –1.748014 |
| 1 | 0 | –0.261306 | 2.849741 | –3.910717 |
| 1 | 0 | 1.857261 | 1.584618 | –3.963712 |
| 6 | 0 | 3.078329 | 0.428229 | –1.886785 |
| 6 | 0 | 3.626266 | –0.011876 | –0.731677 |
| 6 | 0 | 2.871247 | –0.025546 | 0.483005 |
| 6 | 0 | 1.524416 | 0.368455 | 0.477239 |
| 1 | 0 | 3.635295 | 0.394127 | –2.815935 |

| | | | | |
|---|---|---|---|---|
| 1 | 0 | 4.642486 | –0.388147 | –0.706511 |
| 6 | 0 | 3.488565 | –0.435481 | 1.698288 |
| 6 | 0 | 2.794234 | –0.405273 | 2.863157 |
| 6 | 0 | 1.392703 | –0.157557 | 2.858406 |
| 6 | 0 | 0.722497 | 0.057788 | 1.636988 |
| 1 | 0 | 4.537809 | –0.706879 | 1.682162 |
| 1 | 0 | 3.280460 | –0.613454 | 3.809033 |
| 6 | 0 | 0.665868 | –0.126289 | 4.082681 |
| 6 | 0 | –0.665868 | 0.126289 | 4.082681 |
| 6 | 0 | –1.392703 | 0.157557 | 2.858406 |
| 6 | 0 | –0.722497 | –0.057788 | 1.636988 |
| 1 | 0 | 1.209928 | –0.251030 | 5.011530 |
| 1 | 0 | –1.209928 | 0.251030 | 5.011530 |
| 6 | 0 | –2.794234 | 0.405273 | 2.863157 |
| 6 | 0 | –3.488565 | 0.435481 | 1.698288 |
| 6 | 0 | –2.871247 | 0.025546 | 0.483005 |
| 6 | 0 | –1.524416 | –0.368455 | 0.477239 |
| 1 | 0 | –3.280460 | 0.613454 | 3.809033 |
| 1 | 0 | –4.537809 | 0.706879 | 1.682162 |
| 6 | 0 | –3.626266 | 0.011876 | –0.731677 |
| 6 | 0 | –3.078329 | –0.428229 | –1.886785 |
| 6 | 0 | –1.786427 | –1.039213 | –1.883924 |
| 6 | 0 | –1.037135 | –1.085382 | –0.685293 |
| 1 | 0 | –4.642486 | 0.388147 | –0.706511 |
| 1 | 0 | –3.635295 | –0.394127 | –2.815935 |
| 6 | 0 | –1.284317 | –1.660878 | –3.046532 |
| 6 | 0 | –0.114542 | –2.373754 | –3.013818 |
| 6 | 0 | 0.569058 | –2.525838 | –1.795719 |
| 6 | 0 | 0.114555 | –1.902900 | –0.660902 |
| 1 | 0 | –1.857261 | –1.584618 | –3.963712 |
| 1 | 0 | 0.261306 | –2.849741 | –3.910717 |
| 1 | 0 | 1.455918 | –3.145469 | –1.748014 |
| 1 | 0 | 0.639335 | –2.056532 | 0.271501 |

M06–2X/6–311+G(2d,p) with the option of ultrafine grid
Compound **8**:**8**
Symmetry $C_i$
Energy HF = –2615.109370 au
Standard orientation

| | | | | |
|---|---|---|---|---|
| 6 | 0 | 0.033291 | 1.845042 | 3.883828 |
| 6 | 0 | –1.247193 | 2.139717 | 4.406938 |
| 6 | 0 | –2.205373 | 2.757607 | 3.644669 |
| 6 | 0 | –1.928986 | 3.134112 | 2.318722 |
| 6 | 0 | –0.668795 | 2.946413 | 1.814393 |
| 6 | 0 | 0.338286 | 2.332357 | 2.590206 |
| 1 | 0 | –1.472411 | 1.894169 | 5.434915 |
| 1 | 0 | –3.176815 | 2.966850 | 4.074821 |
| 1 | 0 | –2.693283 | 3.609417 | 1.716115 |
| 1 | 0 | –0.416247 | 3.290686 | 0.816860 |
| 6 | 0 | 1.682550 | 2.275785 | 2.107430 |
| 6 | 0 | 2.676195 | 1.841457 | 2.913351 |
| 6 | 0 | 2.396249 | 1.319404 | 4.214447 |
| 6 | 0 | 1.068840 | 1.176424 | 4.649969 |
| 1 | 0 | 1.894285 | 2.653102 | 1.113293 |

| | | | | |
|---|---|---|---|---|
| 1 | 0 | 3.709605 | 1.874508 | 2.587968 |
| 6 | 0 | 3.474530 | 0.978148 | 5.077213 |
| 6 | 0 | 3.231677 | 0.577056 | 6.349442 |
| 6 | 0 | 1.902717 | 0.295789 | 6.771198 |
| 6 | 0 | 0.832516 | 0.428384 | 5.863208 |
| 1 | 0 | 4.488242 | 1.116196 | 4.719957 |
| 1 | 0 | 4.042382 | 0.419167 | 7.050998 |
| 6 | 0 | 1.655503 | −0.134355 | 8.107468 |
| 6 | 0 | 0.393302 | −0.407131 | 8.517379 |
| 6 | 0 | −0.668881 | −0.473033 | 7.569923 |
| 6 | 0 | −0.409903 | −0.232880 | 6.207144 |
| 1 | 0 | 2.489672 | −0.182211 | 8.797465 |
| 1 | 0 | 0.178480 | −0.638444 | 9.553929 |
| 6 | 0 | −1.992488 | −0.782688 | 7.998077 |
| 6 | 0 | −3.006246 | −0.831394 | 7.100550 |
| 6 | 0 | −2.733671 | −0.782129 | 5.702377 |
| 6 | 0 | −1.404345 | −0.666919 | 5.247383 |
| 1 | 0 | −2.175656 | −0.918123 | 9.057419 |
| 1 | 0 | −4.032097 | −0.964803 | 7.423149 |
| 6 | 0 | −3.802273 | −0.834334 | 4.765429 |
| 6 | 0 | −3.548990 | −0.776152 | 3.434176 |
| 6 | 0 | −2.213574 | −0.863466 | 2.952924 |
| 6 | 0 | −1.141961 | −0.948129 | 3.856664 |
| 1 | 0 | −4.818349 | −0.874415 | 5.140137 |
| 1 | 0 | −4.357948 | −0.731820 | 2.714464 |
| 6 | 0 | −1.968458 | −0.890514 | 1.544301 |
| 6 | 0 | −0.715784 | −1.040320 | 1.058468 |
| 6 | 0 | 0.374681 | −1.311424 | 1.942481 |
| 6 | 0 | 0.148759 | −1.359515 | 3.337670 |
| 1 | 0 | −2.810033 | −0.768168 | 0.870414 |
| 1 | 0 | −0.528606 | −1.021264 | −0.008700 |
| 6 | 0 | 1.654678 | −1.612738 | 1.426198 |
| 6 | 0 | 2.657595 | −2.046643 | 2.253537 |
| 6 | 0 | 2.394171 | −2.236146 | 3.621994 |
| 6 | 0 | 1.172058 | −1.903427 | 4.147893 |
| 1 | 0 | 1.814200 | −1.517346 | 0.357132 |
| 1 | 0 | 3.635260 | −2.282098 | 1.851525 |
| 1 | 0 | 3.158931 | −2.649738 | 4.267508 |
| 1 | 0 | 0.984437 | −2.076558 | 5.198347 |
| 6 | 0 | −0.033291 | −1.845042 | −3.883828 |
| 6 | 0 | 1.247193 | −2.139717 | −4.406938 |
| 6 | 0 | 2.205373 | −2.757607 | −3.644669 |
| 6 | 0 | 1.928986 | −3.134112 | −2.318722 |
| 6 | 0 | 0.668795 | −2.946413 | −1.814393 |
| 6 | 0 | −0.338286 | −2.332357 | −2.590206 |
| 1 | 0 | 1.472411 | −1.894169 | −5.434915 |
| 1 | 0 | 3.176815 | −2.966850 | −4.074821 |
| 1 | 0 | 2.693283 | −3.609417 | −1.716115 |
| 1 | 0 | 0.416247 | −3.290686 | −0.816860 |
| 6 | 0 | −1.682550 | −2.275785 | −2.107430 |
| 6 | 0 | −2.676195 | −1.841457 | −2.913351 |
| 6 | 0 | −2.396249 | −1.319404 | −4.214447 |
| 6 | 0 | −1.068840 | −1.176424 | −4.649969 |
| 1 | 0 | −1.894285 | −2.653102 | −1.113293 |
| 1 | 0 | −3.709605 | −1.874508 | −2.587968 |

| | | | | |
|---|---|---|---|---|
| 6 | 0 | −3.474530 | −0.978148 | −5.077213 |
| 6 | 0 | −3.231677 | −0.577056 | −6.349442 |
| 6 | 0 | −1.902717 | −0.295789 | −6.771198 |
| 6 | 0 | −0.832516 | −0.428384 | −5.863208 |
| 1 | 0 | −4.488242 | −1.116196 | −4.719957 |
| 1 | 0 | −4.042382 | −0.419167 | −7.050998 |
| 6 | 0 | −1.655503 | 0.134355 | −8.107468 |
| 6 | 0 | −0.393302 | 0.407131 | −8.517379 |
| 6 | 0 | 0.668881 | 0.473033 | −7.569923 |
| 6 | 0 | 0.409903 | 0.232880 | −6.207144 |
| 1 | 0 | −2.489672 | 0.182211 | −8.797465 |
| 1 | 0 | −0.178480 | 0.638444 | −9.553929 |
| 6 | 0 | 1.992488 | 0.782688 | −7.998077 |
| 6 | 0 | 3.006246 | 0.831394 | −7.100550 |
| 6 | 0 | 2.733671 | 0.782129 | −5.702377 |
| 6 | 0 | 1.404345 | 0.666919 | −5.247383 |
| 1 | 0 | 2.175656 | 0.918123 | −9.057419 |
| 1 | 0 | 4.032097 | 0.964803 | −7.423149 |
| 6 | 0 | 3.802273 | 0.834334 | −4.765429 |
| 6 | 0 | 3.548990 | 0.776152 | −3.434176 |
| 6 | 0 | 2.213574 | 0.863466 | −2.952924 |
| 6 | 0 | 1.141961 | 0.948129 | −3.856664 |
| 1 | 0 | 4.818349 | 0.874415 | −5.140137 |
| 1 | 0 | 4.357948 | 0.731820 | −2.714464 |
| 6 | 0 | 1.968458 | 0.890514 | −1.544301 |
| 6 | 0 | 0.715784 | 1.040320 | −1.058468 |
| 6 | 0 | −0.374681 | 1.311424 | −1.942481 |
| 6 | 0 | −0.148759 | 1.359515 | −3.337670 |
| 1 | 0 | 2.810033 | 0.768168 | −0.870414 |
| 1 | 0 | 0.528606 | 1.021264 | 0.008700 |
| 6 | 0 | −1.654678 | 1.612738 | −1.426198 |
| 6 | 0 | −2.657595 | 2.046643 | −2.253537 |
| 6 | 0 | −2.394171 | 2.236146 | −3.621994 |
| 6 | 0 | −1.172058 | 1.903427 | −4.147893 |
| 1 | 0 | −1.814200 | 1.517346 | −0.357132 |
| 1 | 0 | −3.635260 | 2.282098 | −1.851525 |
| 1 | 0 | −3.158931 | 2.649738 | −4.267508 |
| 1 | 0 | −0.984437 | 2.076558 | −5.198347 |

M06–2X/6–311+G(2d,p) with the option of ultrafine grid
Compound **10**:**10**
Symmetry $C_i$
Energy HF = −3229.593523 au
Standard orientation

| | | | | |
|---|---|---|---|---|
| 6 | 0 | −1.215341 | 2.100184 | 3.408011 |
| 6 | 0 | −2.542289 | 1.867433 | 3.839408 |
| 6 | 0 | −3.593359 | 1.867240 | 2.957501 |
| 6 | 0 | −3.380032 | 2.121359 | 1.591865 |
| 6 | 0 | −2.122913 | 2.454975 | 1.160600 |
| 6 | 0 | −1.033261 | 2.488316 | 2.057954 |
| 1 | 0 | −2.741907 | 1.710755 | 4.889079 |
| 1 | 0 | −4.595681 | 1.682740 | 3.323492 |
| 1 | 0 | −4.209899 | 2.102647 | 0.896218 |
| 1 | 0 | −1.949001 | 2.727743 | 0.124735 |

| | | | | |
|---|---|---|---|---|
| 6 | 0 | 0.230698 | 2.999437 | 1.628725 |
| 6 | 0 | 1.227830 | 3.179735 | 2.521360 |
| 6 | 0 | 1.090427 | 2.755048 | 3.879386 |
| 6 | 0 | −0.068554 | 2.082726 | 4.302177 |
| 1 | 0 | 0.343731 | 3.305341 | 0.594887 |
| 1 | 0 | 2.160361 | 3.646635 | 2.224933 |
| 6 | 0 | 2.121561 | 3.066894 | 4.809091 |
| 6 | 0 | 1.967969 | 2.780052 | 6.124043 |
| 6 | 0 | 0.864368 | 1.998725 | 6.564540 |
| 6 | 0 | −0.069792 | 1.506799 | 5.630293 |
| 1 | 0 | 2.996400 | 3.598631 | 4.452925 |
| 1 | 0 | 2.701626 | 3.097882 | 6.855639 |
| 6 | 0 | 0.702681 | 1.717999 | 7.952627 |
| 6 | 0 | −0.366543 | 1.011708 | 8.393651 |
| 6 | 0 | −1.226700 | 0.363406 | 7.462108 |
| 6 | 0 | −0.962031 | 0.462802 | 6.085725 |
| 1 | 0 | 1.423356 | 2.130970 | 8.648832 |
| 1 | 0 | −0.550018 | 0.875446 | 9.452837 |
| 6 | 0 | −2.346361 | −0.398228 | 7.911863 |
| 6 | 0 | −3.162277 | −1.014162 | 7.020201 |
| 6 | 0 | −2.789290 | −1.096447 | 5.645922 |
| 6 | 0 | −1.587655 | −0.508061 | 5.221095 |
| 1 | 0 | −2.560508 | −0.423630 | 8.973859 |
| 1 | 0 | −4.071210 | −1.508318 | 7.342935 |
| 6 | 0 | −3.613325 | −1.770408 | 4.696977 |
| 6 | 0 | −3.244580 | −1.833781 | 3.392562 |
| 6 | 0 | −1.939831 | −1.421805 | 2.990509 |
| 6 | 0 | −1.045176 | −0.918285 | 3.950130 |
| 1 | 0 | −4.561236 | −2.177489 | 5.029173 |
| 1 | 0 | −3.907893 | −2.253948 | 2.645662 |
| 6 | 0 | −1.528756 | −1.517854 | 1.629857 |
| 6 | 0 | −0.291706 | −1.101888 | 1.260526 |
| 6 | 0 | 0.682170 | −0.751103 | 2.241541 |
| 6 | 0 | 0.356710 | −0.837103 | 3.608708 |
| 1 | 0 | −2.236847 | −1.877111 | 0.890341 |
| 1 | 0 | −0.001144 | −1.086075 | 0.217118 |
| 6 | 0 | 1.983358 | −0.329441 | 1.845413 |
| 6 | 0 | 2.914778 | −0.025523 | 2.784558 |
| 6 | 0 | 2.675317 | −0.312153 | 4.159167 |
| 6 | 0 | 1.441160 | −0.845292 | 4.562837 |
| 1 | 0 | 2.190554 | −0.221015 | 0.785684 |
| 1 | 0 | 3.882157 | 0.372892 | 2.500136 |
| 6 | 0 | 3.706756 | −0.084617 | 5.123808 |
| 6 | 0 | 3.539721 | −0.431074 | 6.419402 |
| 6 | 0 | 2.385161 | −1.170334 | 6.823741 |
| 6 | 0 | 1.366456 | −1.448051 | 5.882279 |
| 1 | 0 | 4.619496 | 0.396631 | 4.791297 |
| 1 | 0 | 4.306909 | −0.221068 | 7.155912 |
| 6 | 0 | 2.296413 | −1.698416 | 8.129625 |
| 6 | 0 | 1.277699 | −2.544518 | 8.479810 |
| 6 | 0 | 0.335116 | −2.927682 | 7.509876 |
| 6 | 0 | 0.383604 | −2.398236 | 6.245596 |
| 1 | 0 | 3.070253 | −1.441810 | 8.844342 |
| 1 | 0 | 1.222284 | −2.950120 | 9.482193 |
| 1 | 0 | −0.429936 | −3.653319 | 7.756175 |

| | | | | |
|---|---|---|---|---|
| 1 | 0 | –0.331230 | –2.733914 | 5.508189 |
| 6 | 0 | 1.215341 | –2.100184 | –3.408011 |
| 6 | 0 | 2.542289 | –1.867433 | –3.839408 |
| 6 | 0 | 3.593359 | –1.867240 | –2.957501 |
| 6 | 0 | 3.380032 | –2.121359 | –1.591865 |
| 6 | 0 | 2.122913 | –2.454975 | –1.160600 |
| 6 | 0 | 1.033261 | –2.488316 | –2.057954 |
| 1 | 0 | 2.741907 | –1.710755 | –4.889079 |
| 1 | 0 | 4.595681 | –1.682740 | –3.323492 |
| 1 | 0 | 4.209899 | –2.102647 | –0.896218 |
| 1 | 0 | 1.949001 | –2.727743 | –0.124735 |
| 6 | 0 | –0.230698 | –2.999437 | –1.628725 |
| 6 | 0 | –1.227830 | –3.179735 | –2.521360 |
| 6 | 0 | –1.090427 | –2.755048 | –3.879386 |
| 6 | 0 | 0.068554 | –2.082726 | –4.302177 |
| 1 | 0 | –0.343731 | –3.305341 | –0.594887 |
| 1 | 0 | –2.160361 | –3.646635 | –2.224933 |
| 6 | 0 | –2.121561 | –3.066894 | –4.809091 |
| 6 | 0 | –1.967969 | –2.780052 | –6.124043 |
| 6 | 0 | –0.864368 | –1.998725 | –6.564540 |
| 6 | 0 | 0.069792 | –1.506799 | –5.630293 |
| 1 | 0 | –2.996400 | –3.598631 | –4.452925 |
| 1 | 0 | –2.701626 | –3.097882 | –6.855639 |
| 6 | 0 | –0.702681 | –1.717999 | –7.952627 |
| 6 | 0 | 0.366543 | –1.011708 | –8.393651 |
| 6 | 0 | 1.226700 | –0.363406 | –7.462108 |
| 6 | 0 | 0.962031 | –0.462802 | –6.085725 |
| 1 | 0 | –1.423356 | –2.130970 | –8.648832 |
| 1 | 0 | 0.550018 | –0.875446 | –9.452837 |
| 6 | 0 | 2.346361 | 0.398228 | –7.911863 |
| 6 | 0 | 3.162277 | 1.014162 | –7.020201 |
| 6 | 0 | 2.789290 | 1.096447 | –5.645922 |
| 6 | 0 | 1.587655 | 0.508061 | –5.221095 |
| 1 | 0 | 2.560508 | 0.423630 | –8.973859 |
| 1 | 0 | 4.071210 | 1.508318 | –7.342935 |
| 6 | 0 | 3.613325 | 1.770408 | –4.696977 |
| 6 | 0 | 3.244580 | 1.833781 | –3.392562 |
| 6 | 0 | 1.939831 | 1.421805 | –2.990509 |
| 6 | 0 | 1.045176 | 0.918285 | –3.950130 |
| 1 | 0 | 4.561236 | 2.177489 | –5.029173 |
| 1 | 0 | 3.907893 | 2.253948 | –2.645662 |
| 6 | 0 | 1.528756 | 1.517854 | –1.629857 |
| 6 | 0 | 0.291706 | 1.101888 | –1.260526 |
| 6 | 0 | –0.682170 | 0.751103 | –2.241541 |
| 6 | 0 | –0.356710 | 0.837103 | –3.608708 |
| 1 | 0 | 2.236847 | 1.877111 | –0.890341 |
| 1 | 0 | 0.001144 | 1.086075 | –0.217118 |
| 6 | 0 | –1.983358 | 0.329441 | –1.845413 |
| 6 | 0 | –2.914778 | 0.025523 | –2.784558 |
| 6 | 0 | –2.675317 | 0.312153 | –4.159167 |
| 6 | 0 | –1.441160 | 0.845292 | –4.562837 |
| 1 | 0 | –2.190554 | 0.221015 | –0.785684 |
| 1 | 0 | –3.882157 | –0.372892 | –2.500136 |
| 6 | 0 | –3.706756 | 0.084617 | –5.123808 |
| 6 | 0 | –3.539721 | 0.431074 | –6.419402 |

| | | | | |
|---|---|---|---|---|
| 6 | 0 | −2.385161 | 1.170334 | −6.823741 |
| 6 | 0 | −1.366456 | 1.448051 | −5.882279 |
| 1 | 0 | −4.619496 | −0.396631 | −4.791297 |
| 1 | 0 | −4.306909 | 0.221068 | −7.155912 |
| 6 | 0 | −2.296413 | 1.698416 | −8.129625 |
| 6 | 0 | −1.277699 | 2.544518 | −8.479810 |
| 6 | 0 | −0.335116 | 2.927682 | −7.509876 |
| 6 | 0 | −0.383604 | 2.398236 | −6.245596 |
| 1 | 0 | −3.070253 | 1.441810 | −8.844342 |
| 1 | 0 | −1.222284 | 2.950120 | −9.482193 |
| 1 | 0 | 0.429936 | 3.653319 | −7.756175 |
| 1 | 0 | 0.331230 | 2.733914 | −5.508189 |

LC−ωPBE/6−311+G(2d,p)
Compound **7**
Symmetry $C_2$
Energy HF = −1153.461248 au

| | | | | |
|---|---|---|---|---|
| 6 | 0 | 1.119731 | 1.075372 | −0.696548 |
| 6 | 0 | 0.004804 | 1.935748 | −0.664316 |
| 6 | 0 | −0.364750 | 2.663612 | −1.757775 |
| 6 | 0 | 0.364750 | 2.574615 | −2.949268 |
| 6 | 0 | 1.489759 | 1.807144 | −2.987198 |
| 6 | 0 | 1.907055 | 1.078594 | −1.858838 |
| 1 | 0 | −0.553642 | 2.049345 | 0.254076 |
| 1 | 0 | −1.222523 | 3.323617 | −1.696672 |
| 1 | 0 | 0.056210 | 3.140745 | −3.820778 |
| 1 | 0 | 2.099914 | 1.767813 | −3.883614 |
| 6 | 0 | 3.172023 | 0.417538 | −1.859729 |
| 6 | 0 | 3.660010 | −0.104581 | −0.723552 |
| 6 | 0 | 2.872348 | −0.141264 | 0.467943 |
| 6 | 0 | 1.550543 | 0.297544 | 0.449567 |
| 1 | 0 | 3.758337 | 0.415107 | −2.772331 |
| 1 | 0 | 4.658926 | −0.526504 | −0.689604 |
| 6 | 0 | 3.455259 | −0.607489 | 1.675817 |
| 6 | 0 | 2.755061 | −0.564837 | 2.826082 |
| 6 | 0 | 1.372066 | −0.243276 | 2.811669 |
| 6 | 0 | 0.724666 | 0.013680 | 1.598622 |
| 1 | 0 | 4.492830 | −0.923594 | 1.665206 |
| 1 | 0 | 3.219341 | −0.815643 | 3.773680 |
| 6 | 0 | 0.650966 | −0.169921 | 4.035221 |
| 6 | 0 | −0.650966 | 0.169921 | 4.035221 |
| 6 | 0 | −1.372066 | 0.243276 | 2.811669 |
| 6 | 0 | −0.724666 | −0.013680 | 1.598622 |
| 1 | 0 | 1.188711 | −0.327394 | 4.963857 |
| 1 | 0 | −1.188711 | 0.327394 | 4.963857 |
| 6 | 0 | −2.755061 | 0.564837 | 2.826082 |
| 6 | 0 | −3.455259 | 0.607489 | 1.675817 |
| 6 | 0 | −2.872348 | 0.141264 | 0.467943 |
| 6 | 0 | −1.550543 | −0.297544 | 0.449567 |
| 1 | 0 | −3.219341 | 0.815643 | 3.773680 |
| 1 | 0 | −4.492830 | 0.923594 | 1.665206 |
| 6 | 0 | −3.660010 | 0.104581 | −0.723552 |
| 6 | 0 | −3.172023 | −0.417538 | −1.859729 |
| 6 | 0 | −1.907055 | −1.078594 | −1.858838 |

| | | | | |
|---|---|---|---|---|
| 6 | 0 | −1.119731 | −1.075372 | −0.696548 |
| 1 | 0 | −4.658926 | 0.526504 | −0.689604 |
| 1 | 0 | −3.758337 | −0.415107 | −2.772331 |
| 6 | 0 | −1.489759 | −1.807144 | −2.987198 |
| 6 | 0 | −0.364750 | −2.574615 | −2.949268 |
| 6 | 0 | 0.364750 | −2.663612 | −1.757775 |
| 6 | 0 | −0.004804 | −1.935748 | −0.664316 |
| 1 | 0 | −2.099914 | −1.767813 | −3.883614 |
| 1 | 0 | −0.056210 | −3.140745 | −3.820778 |
| 1 | 0 | 1.222523 | −3.323617 | −1.696672 |
| 1 | 0 | 0.553642 | −2.049345 | 0.254076 |

LC–ωPBE/6–311+G(2d,p) with the option of ultrafine grid
Compound **7:7**
Symmetry $C_i$
Energy HF = −2306.940209 au

| | | | | |
|---|---|---|---|---|
| 6 | 0 | 3.888860 | −0.258523 | 1.823348 |
| 1 | 0 | 4.952350 | −0.363587 | 1.985324 |
| 6 | 0 | 3.031396 | −1.049088 | 2.532756 |
| 1 | 0 | 3.428821 | −1.782196 | 3.225644 |
| 6 | 0 | 1.646328 | −0.908038 | 2.381989 |
| 1 | 0 | 0.968739 | −1.551258 | 2.932473 |
| 6 | 0 | 1.158308 | 0.075553 | 1.573746 |
| 1 | 0 | 0.088982 | 0.238304 | 1.485798 |
| 6 | 0 | 2.028172 | 0.923793 | 0.863748 |
| 6 | 0 | 1.507142 | 2.051129 | 0.159240 |
| 1 | 0 | 0.431367 | 2.183199 | 0.111755 |
| 6 | 0 | 2.343899 | 2.969139 | −0.351608 |
| 1 | 0 | 1.961259 | 3.875985 | −0.808341 |
| 6 | 0 | 3.758992 | 2.772364 | −0.327110 |
| 6 | 0 | 4.614868 | 3.810952 | −0.780439 |
| 1 | 0 | 4.170493 | 4.733276 | −1.138980 |
| 6 | 0 | 5.952263 | 3.681778 | −0.673663 |
| 1 | 0 | 6.614839 | 4.506437 | −0.913484 |
| 6 | 0 | 6.524201 | 2.438684 | −0.294063 |
| 6 | 0 | 7.934308 | 2.325687 | −0.147259 |
| 1 | 0 | 8.544018 | 3.210184 | −0.296906 |
| 6 | 0 | 8.483113 | 1.162752 | 0.250904 |
| 1 | 0 | 9.543724 | 1.088562 | 0.465481 |
| 6 | 0 | 7.692127 | −0.017562 | 0.313331 |
| 6 | 0 | 8.289451 | −1.247053 | 0.698452 |
| 1 | 0 | 9.329746 | −1.245054 | 1.005583 |
| 6 | 0 | 7.557849 | −2.378577 | 0.729047 |
| 1 | 0 | 7.983013 | −3.308364 | 1.091501 |
| 6 | 0 | 6.244489 | −2.393197 | 0.189055 |
| 6 | 0 | 5.522354 | −3.624804 | 0.134954 |
| 1 | 0 | 5.964439 | −4.498481 | 0.602291 |
| 6 | 0 | 4.321867 | −3.698032 | −0.462008 |
| 1 | 0 | 3.761782 | −4.626926 | −0.476782 |
| 6 | 0 | 3.809586 | −2.575228 | −1.179108 |
| 6 | 0 | 2.665951 | −2.716267 | −1.986299 |
| 1 | 0 | 2.132482 | −3.661224 | −1.969573 |
| 6 | 0 | 2.256357 | −1.706582 | −2.805526 |
| 1 | 0 | 1.381756 | −1.829852 | −3.434388 |

| | | | | |
|---|---|---|---|---|
| 6 | 0 | 3.004375 | –0.523958 | –2.861633 |
| 1 | 0 | 2.726081 | 0.259431 | –3.557615 |
| 6 | 0 | 4.095446 | –0.355659 | –2.058467 |
| 1 | 0 | 4.676067 | 0.551611 | –2.148526 |
| 6 | 0 | 4.503462 | –1.354573 | –1.152356 |
| 6 | 0 | 5.675360 | –1.222406 | –0.306861 |
| 6 | 0 | 6.333183 | 0.029174 | –0.015469 |
| 6 | 0 | 5.701526 | 1.333611 | –0.050747 |
| 6 | 0 | 4.295648 | 1.583145 | 0.161670 |
| 6 | 0 | 3.415650 | 0.714801 | 0.921046 |
| 6 | 0 | –3.888860 | 0.258523 | –1.823348 |
| 1 | 0 | –4.952350 | 0.363587 | –1.985324 |
| 6 | 0 | –3.031396 | 1.049088 | –2.532756 |
| 1 | 0 | –3.428821 | 1.782196 | –3.225644 |
| 6 | 0 | –1.646328 | 0.908038 | –2.381989 |
| 1 | 0 | –0.968739 | 1.551258 | –2.932473 |
| 6 | 0 | –1.158308 | –0.075553 | –1.573746 |
| 1 | 0 | –0.088982 | –0.238304 | –1.485798 |
| 6 | 0 | –2.028172 | –0.923793 | –0.863748 |
| 6 | 0 | –1.507142 | –2.051129 | –0.159240 |
| 1 | 0 | –0.431367 | –2.183199 | –0.111755 |
| 6 | 0 | –2.343899 | –2.969139 | 0.351608 |
| 1 | 0 | –1.961259 | –3.875985 | 0.808341 |
| 6 | 0 | –3.758992 | –2.772364 | 0.327110 |
| 6 | 0 | –4.614868 | –3.810952 | 0.780439 |
| 1 | 0 | –4.170493 | –4.733276 | 1.138980 |
| 6 | 0 | –5.952263 | –3.681778 | 0.673663 |
| 1 | 0 | –6.614839 | –4.506437 | 0.913484 |
| 6 | 0 | –6.524201 | –2.438684 | 0.294063 |
| 6 | 0 | –7.934308 | –2.325687 | 0.147259 |
| 1 | 0 | –8.544018 | –3.210184 | 0.296906 |
| 6 | 0 | –8.483113 | –1.162752 | –0.250904 |
| 1 | 0 | –9.543724 | –1.088562 | –0.465481 |
| 6 | 0 | –7.692127 | 0.017562 | –0.313331 |
| 6 | 0 | –8.289451 | 1.247053 | –0.698452 |
| 1 | 0 | –9.329746 | 1.245054 | –1.005583 |
| 6 | 0 | –7.557849 | 2.378577 | –0.729047 |
| 1 | 0 | –7.983013 | 3.308364 | –1.091501 |
| 6 | 0 | –6.244489 | 2.393197 | –0.189055 |
| 6 | 0 | –5.522354 | 3.624804 | –0.134954 |
| 1 | 0 | –5.964439 | 4.498481 | –0.602291 |
| 6 | 0 | –4.321867 | 3.698032 | 0.462008 |
| 1 | 0 | –3.761782 | 4.626926 | 0.476782 |
| 6 | 0 | –3.809586 | 2.575228 | 1.179108 |
| 6 | 0 | –2.665951 | 2.716267 | 1.986299 |
| 1 | 0 | –2.132482 | 3.661224 | 1.969573 |
| 6 | 0 | –2.256357 | 1.706582 | 2.805526 |
| 1 | 0 | –1.381756 | 1.829852 | 3.434388 |
| 6 | 0 | –3.004375 | 0.523958 | 2.861633 |
| 1 | 0 | –2.726081 | –0.259431 | 3.557615 |
| 6 | 0 | –4.095446 | 0.355659 | 2.058467 |
| 1 | 0 | –4.676067 | –0.551611 | 2.148526 |
| 6 | 0 | –4.503462 | 1.354573 | 1.152356 |
| 6 | 0 | –5.675360 | 1.222406 | 0.306861 |
| 6 | 0 | –6.333183 | –0.029174 | 0.015469 |

| | | | | |
|---|---|---|---|---|
| 6 | 0 | –5.701526 | –1.333611 | 0.050747 |
| 6 | 0 | –4.295648 | –1.583145 | –0.161670 |
| 6 | 0 | –3.415650 | –0.714801 | –0.921046 |

**Appendix**

QTAIM Dual Functional Analysis (QTAIM–DFA)

The bond critical point (BCP; ∗) is an important concept in QTAIM. The BCP of ($\omega$, $\sigma$) = (3, –1)[SA1] is a point along the bond path (BP) at the interatomic surface, where charge density $\rho(\boldsymbol{r})$ reaches a minimum. It is donated by $\rho_b(\boldsymbol{r}_c)$, so are other QTAIM functions, such as the total electron energy densities $H_b(\boldsymbol{r}_c)$, potential energy densities $V_b(\boldsymbol{r}_c)$ and kinetic energy densities $G_b(\boldsymbol{r}_c)$ at the BCPs. A chemical bond or interaction between A and B is denoted by A–B, which corresponds to the BP between A and B in QTAIM. We will use A–∗–B for BP, where the asterisk emphasizes the presence of a BCP in A–B.

The sign of the Laplacian $\rho_b(\boldsymbol{r}_c)$ ($\nabla^2\rho_b(\boldsymbol{r}_c)$) indicates that $\rho_b(\boldsymbol{r}_c)$ is depleted or concentrated with respect to its surrounding, since $\nabla^2\rho_b(\boldsymbol{r}_c)$ is the second derivative of $\rho_b(\boldsymbol{r}_c)$. $\rho_b(\boldsymbol{r}_c)$ is locally depleted relative to the average distribution around $\boldsymbol{r}_c$ if $\nabla^2\rho_b(\boldsymbol{r}_c) > 0$, but it is concentrated when $\nabla^2\rho_b(\boldsymbol{r}_c) < 0$. Total electron energy densities at BCPs ($H_b(\boldsymbol{r}_c)$) must be a more appropriate measure for weak interactions on the energy basis.[SA1–SA8] $H_b(\boldsymbol{r}_c)$ are the sum of kinetic energy densities ($G_b(\boldsymbol{r}_c)$) and potential energy densities ($V_b(\boldsymbol{r}_c)$) at BCPs, as shown in Equation (SA1). Electrons at BCPs are stabilized when $H_b(\boldsymbol{r}_c) < 0$, therefore, interactions exhibit the covalent nature in this region, whereas they exhibit no covalency if $H_b(\boldsymbol{r}_c) > 0$, due to the destabilization of electrons at BCPs under the conditions.[SA1] Equation (SA2) represents the relation between $\nabla^2\rho_b(\boldsymbol{r}_c)$ and $H_b(\boldsymbol{r}_c)$, together with $G_b(\boldsymbol{r}_c)$ and $V_b(\boldsymbol{r}_c)$, which is closely related to the virial theorem.

$$H_b(\boldsymbol{r}_c) = G_b(\boldsymbol{r}_c) + V_b(\boldsymbol{r}_c) \qquad \text{(SA1)}$$
$$(\hbar^2/8m)\nabla^2\rho_b(\boldsymbol{r}_c) = H_b(\boldsymbol{r}_c) - V_b(\boldsymbol{r}_c)/2 \qquad \text{(SA2)}$$
$$= G_b(\boldsymbol{r}_c) + V_b(\boldsymbol{r}_c)/2 \qquad \text{(SA2')}$$

Interactions are classified by the signs of $\nabla^2\rho_b(\boldsymbol{r}_c)$ and $H_b(\boldsymbol{r}_c)$. Interactions in the region of $\nabla^2\rho_b(\boldsymbol{r}_c) < 0$ are called shared–shell (SS) interactions and they are closed–shell (CS) interactions for $\nabla^2\rho_b(\boldsymbol{r}_c) > 0$. $H_b(\boldsymbol{r}_c)$ must be negative when $\nabla^2\rho_b(\boldsymbol{r}_c) < 0$, since $H_b(\boldsymbol{r}_c)$ are larger than $(\hbar^2/8m)\nabla^2\rho_b(\boldsymbol{r}_c)$ by $V_b(\boldsymbol{r}_c)/2$ with negative $V_b(\boldsymbol{r}_c)$ at all BCPs (Equation (SA2)). Consequently, $\nabla^2\rho_b(\boldsymbol{r}_c) < 0$ and $H_b(\boldsymbol{r}_c) < 0$ for the SS interactions. The CS interactions are especially called *pure* CS interactions for $H_b(\boldsymbol{r}_c) > 0$ and $\nabla^2\rho_b(\boldsymbol{r}_c) > 0$, since electrons at BCPs are depleted and destabilized under the conditions.[SA1a] Electrons in the intermediate region between SS and *pure* CS, which belong to CS, are locally depleted but stabilized at BCPs, since $\nabla^2\rho_b(\boldsymbol{r}_c) > 0$ but $H_b(\boldsymbol{r}_c) < 0$.[SA1a] We call the interactions in this region *regular* CS,[SA4,SA5] when it is necessary to distinguish from *pure* CS. The role of $\nabla^2\rho_b(\boldsymbol{r}_c)$ in the classification can be replaced by $H_b(\boldsymbol{r}_c) - V_b(\boldsymbol{r}_c)/2$, since $(\hbar^2/8m)\nabla^2\rho_b(\boldsymbol{r}_c) = H_b(\boldsymbol{r}_c) - V_b(\boldsymbol{r}_c)/2$ (Equation (SA2)). Scheme SA1 summarizes the classification.

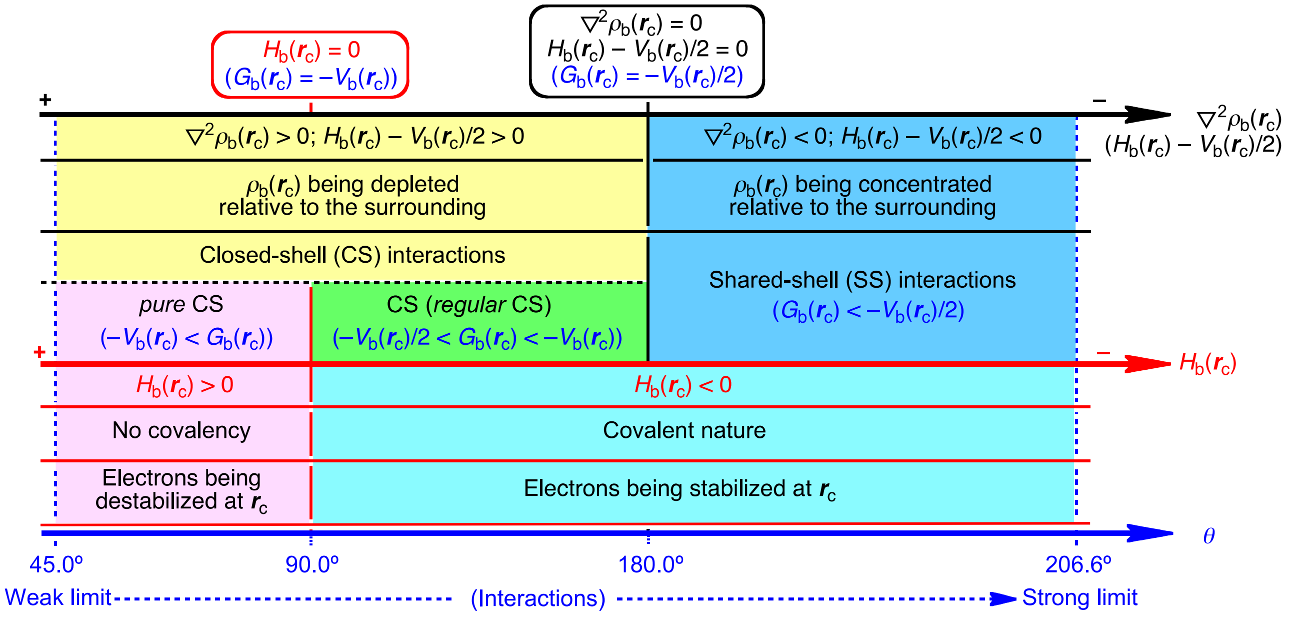


**Scheme S1.** Classification of interactions by the signs of $\nabla^2\rho_b(r_c)$ and $H_b(r_c)$, together with $G_b(r_c)$ and $V_b(r_c)$.

We proposed QTAIM–DFA by plotting $H_b(r_c)$ versus $H_b(r_c) - V_b(r_c)/2$ (= $(\hbar^2/8m)\nabla^2\rho_b(r_c)$),[SA4a] after the proposal of $H_b(r_c)$ versus $\nabla^2\rho_b(r_c)$.[SA4b] Both axes in the plot of the former are given in energy unit, therefore, distances on the $(x, y)$ (= $(H_b(r_c) - V_b(r_c)/2$, $H_b(r_c))$) plane can be expressed in the energy unit, which provides an analytical development. QTAIM–DFA incorporates the classification of interactions by the signs of $\nabla^2\rho_b(r_c)$ and $H_b(r_c)$. Scheme SA2 summarizes the QTAIM–DFA treatment. Interactions of *pure* CS appear in the first quadrant, those of *regular* CS in the fourth quadrant and SS interactions do in the third quadrant. No interactions appear in the second one.

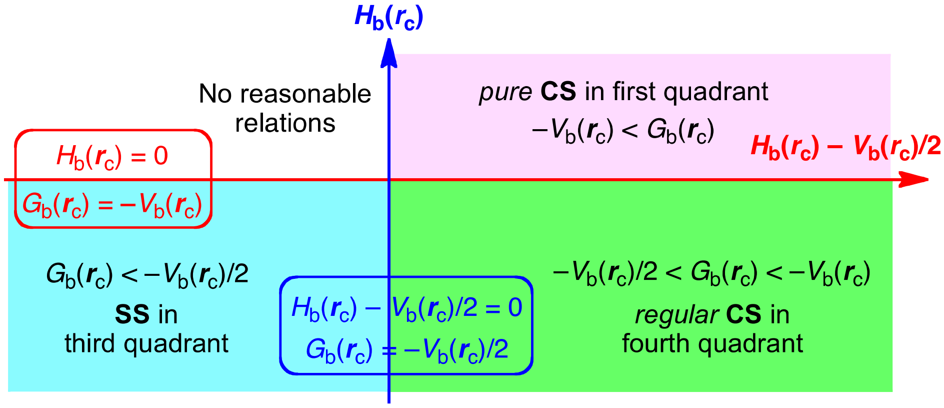


**Scheme 2.** QTAIM–DFA: Plot of $H_b(r_c)$ versus $H_b(r_c) - V_b(r_c)/2$ for Weak to Strong Interactions.

In our treatment, data for perturbed structures around fully optimized structures are also employed for the plots, together with the fully optimized ones (see Fig. SA1).[SA4–SA8] We proposed the concept of the "dynamic nature of interaction" originated from the perturbed structures. The behavior of interactions at the fully optimized structures corresponds to "the static nature of interactions", whereas that containing perturbed structures exhibit the "dynamic nature of interaction" as explained below. The method to generate the perturbed structures is discussed later. Plots of $H_b(r_c)$ versus $H_b(r_c) - V_b(r_c)/2$ are analyzed employing the polar coordinate $(R, \theta)$ representation with $(\theta_p, \kappa_p)$ parameters.[SA4a,SA5–SA8] Fig. SA1 explains the treatment. $R$ in $(R, \theta)$ is defined by Equation (SA3) and given in the energy unit. Indeed, $R$ does not correspond to the usual interaction energy, but it does to the local energy at BCP, expressed by $[(H_b(r_c))^2 + (H_b(r_c) - V_b(r_c)/2)^2]^{1/2}$ in the plot (cf:

Equation (SA3)), where $R = 0$ for the enough large interaction distance. The plots show a spiral stream, as a whole. $\theta$ in ($R$, $\theta$) defined by Equation (SA4), measured from the $y$–axis, controls the spiral stream of the plot. Each plot for an interaction shows a specific curve, which provides important information of the interaction (see Fig. SA1). The curve is expressed by $\theta_p$ and $\kappa_p$. While $\theta_p$, defined by Equation (SA5) and measured from the $y$–direction, corresponds to the tangent line of a plot, where $\theta_p$ is calculated employing data of the perturbed structures with a fully–optimized structure and $\kappa_p$ is the curvature of the plot (Equation (SA6)). While ($R$, $\theta$) correspond to the static nature, ($\theta_p$, $\kappa_p$) represent the dynamic nature of interactions. We call ($R$, $\theta$) and ($\theta_p$, $\kappa_p$) QTAIM–DFA parameters, whereas $\rho_b(\boldsymbol{r}_c)$, $\nabla^2\rho_b(\boldsymbol{r}_c)$, $G_b(\boldsymbol{r}_c)$, $V_b(\boldsymbol{r}_c)$, $H_b(\boldsymbol{r}_c)$ and $H_b(\boldsymbol{r}_c) - V_b(\boldsymbol{r}_c)/2$ belong to QTAIM functions. $k_b(\boldsymbol{r}_c)$, defined by Equation (SA7), is an QTAIM function but it will be treated as if it were an QTAIM–DFA parameter, if suitable.

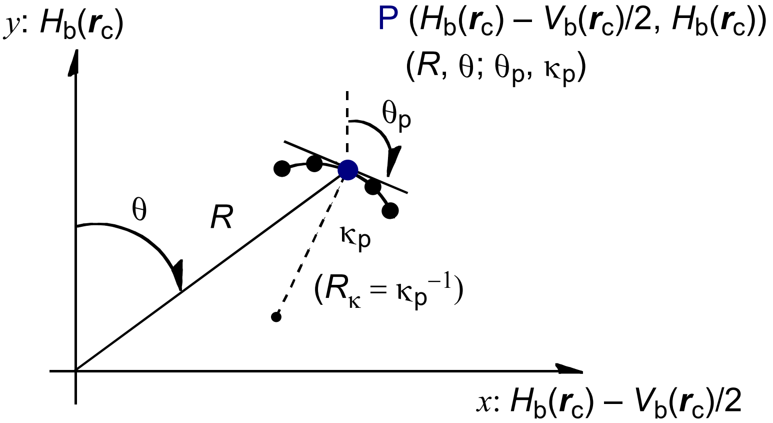


**Figure SA1.** Polar ($R$, $\theta$) coordinate representation of $H_b(\boldsymbol{r}_c)$ versus $H_b(\boldsymbol{r}_c) - V_b(\boldsymbol{r}_c)/2$, with ($\theta_p$, $\kappa_p$) parameters.

$R = (x^2 + y^2)^{1/2}$ (SA3)
$\theta = 90^{\circ} - \tan^{-1}(y/x)$ (SA4)
$\theta_p = 90^{\circ} - \tan^{-1}(dy/dx)$ (SA5)
$\kappa_p = |d^2y/dx^2|/[1 + (dy/dx)^2]^{3/2}$ (SA6)
$k_b(\boldsymbol{r}_c) = V_b(\boldsymbol{r}_c)/G_b(\boldsymbol{r}_c)$ (SA7)
where $(x, y) = (H_b(\boldsymbol{r}_c) - V_b(\boldsymbol{r}_c)/2, H_b(\boldsymbol{r}_c))$

**Criteria for Classification of Interactions: Behavior of Typical Interactions Elucidated by QTAIM–DFA**

$H_b(\boldsymbol{r}_c)$ are plotted versus $H_b(\boldsymbol{r}_c) - V_b(\boldsymbol{r}_c)/2$ for typical interactions in vdW (van der Waals interactions), HBs (hydrogen bonds), CT–MCs (molecular complexes through charge transfer), $X_3^-$ (trihalide ions), CT–TBPs (trigonal bipyramidal adducts through charge–transfer), Cov–w (weak covalent bonds) and Cov–s (strong covalent bonds).[SA4–SA8] Rough criteria are obtained by applying QTAIM–DFA, after the analysis of the plots for the typical interactions according to Equations (SA3)–(SA7). Scheme SA3 shows the rough criteria, which are accomplished by the $\theta$ and $\theta_p$ values, together with the values of $k_b(\boldsymbol{r}_c)$. The criteria will be employed to discuss the nature of interactions in question, as a reference.

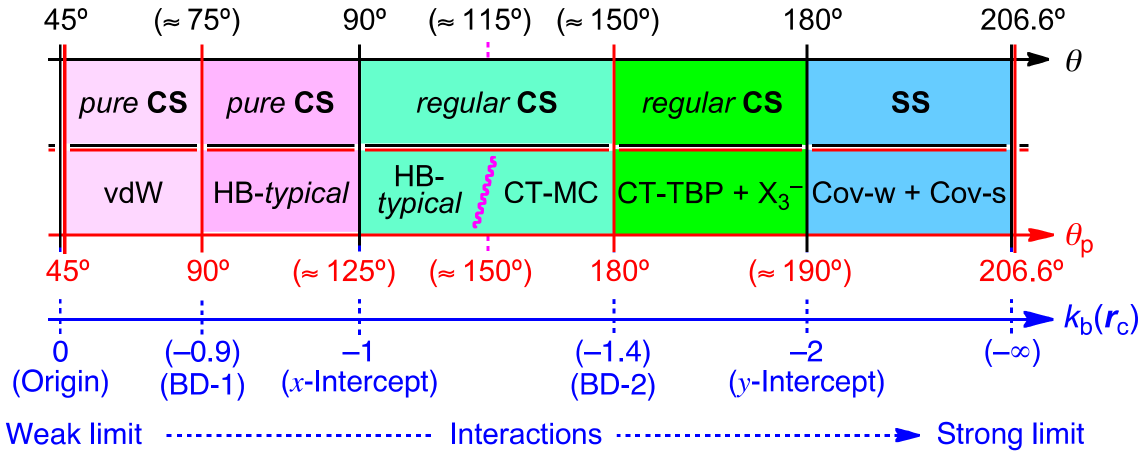


**Scheme SA3.** Rough classification and characterization of interactions by $\theta$ and $\theta_p$, together with $k_b(\mathbf{r}_c)$ (= $V_b(\mathbf{r}_c)/G_b(\mathbf{r}_c)$).

**Characterization of interactions**

The characterization of interactions is explained employing [$^1$Cl–$^2$Cl–$^3$Cl]$^-$. The wide range of the perturbed structures were generated by partially optimizing $r$($^2$Cl–$^3$Cl) in [$^1$Cl–$^2$Cl–$^3$Cl]$^-$, assuming the $C_{\infty v}$ symmetry, with $r$($^1$Cl–$^2$Cl) being fixed in the wide range. The partial optimization method is called POM.[SA4b,SA5] The QTAIM functions, such as $V_b(\mathbf{r}_c)$, $G_b(\mathbf{r}_c)$, $H_b(\mathbf{r}_c)$, $H_b(\mathbf{r}_c) - V_b(\mathbf{r}_c)/2$ are calculated at BCPs for the wide varieties of the perturbed structures of [$^1$Cl–$^2$Cl–$^3$Cl]$^-$. $H_b(\mathbf{r}_c) - V_b(\mathbf{r}_c)/2$ and $H_b(\mathbf{r}_c)$ are plotted versus the interaction distances $r$($^1$Cl–$^2$Cl) in the perturbed structures of [$^1$Cl–$^2$Cl–$^3$Cl]$^-$, in the wide range. Fig. SA2 shows the plots. Each plot is analyzed using a regression curve of the ninth function and the first derivative of each regression curve is obtained. As shown in Fig. SA2, the maximum value of $H_b(\mathbf{r}_c)$ ($\mathrm{d}(H_b(\mathbf{r}_c)/\mathrm{d}r = 0$) is defined as the borderline between vdW and t–HB interactions. Similarly, the maximum value of $H_b(\mathbf{r}_c) - V_b(\mathbf{r}_c)/2$ ($\mathrm{d}(H_b(\mathbf{r}_c) - V_b(\mathbf{r}_c)/2)/\mathrm{d}r = 0$) does to the borderline between CT–MC and CT–TBP. However, it seems difficult to find a characteristic point corresponding to the borderline between $t$–HB and CT–MC in nature. Therefore, the borderline is tentatively given by $\theta_p = 150^{\circ}$ based on the expectation form the experimental results, where $\theta_p$ is defined by $[90^{\circ} - \tan^{-1}[\mathrm{d}H_b(\mathbf{r}_c)/\mathrm{d}(H_b(\mathbf{r}_c) - V_b(\mathbf{r}_c)/2)]]$ in the plot of $H_b(\mathbf{r}_c)$ versus $H_b(\mathbf{r}_c) - V_b(\mathbf{r}_c)/2$. The proposed classification and characterization of interactions, by means of the QTAIM functions of $H_b(\mathbf{r}_c)$, $H_b(\mathbf{r}_c) - V_b(\mathbf{r}_c)/2$, $G_b(\mathbf{r}_c)$ and/or $V_b(\mathbf{r}_c)$, are summarized in Table SA1. The plot of $H_b(\mathbf{r}_c) - V_b(\mathbf{r}_c)/2$ versus $w$ in Fig. SA2 is essentially the same as that of $\nabla^2\rho_b(\mathbf{r}_c)$ versus $d$(H⋯F) in X–H⋯F–Y, presented by Espinosa and co–workers.[SA9]

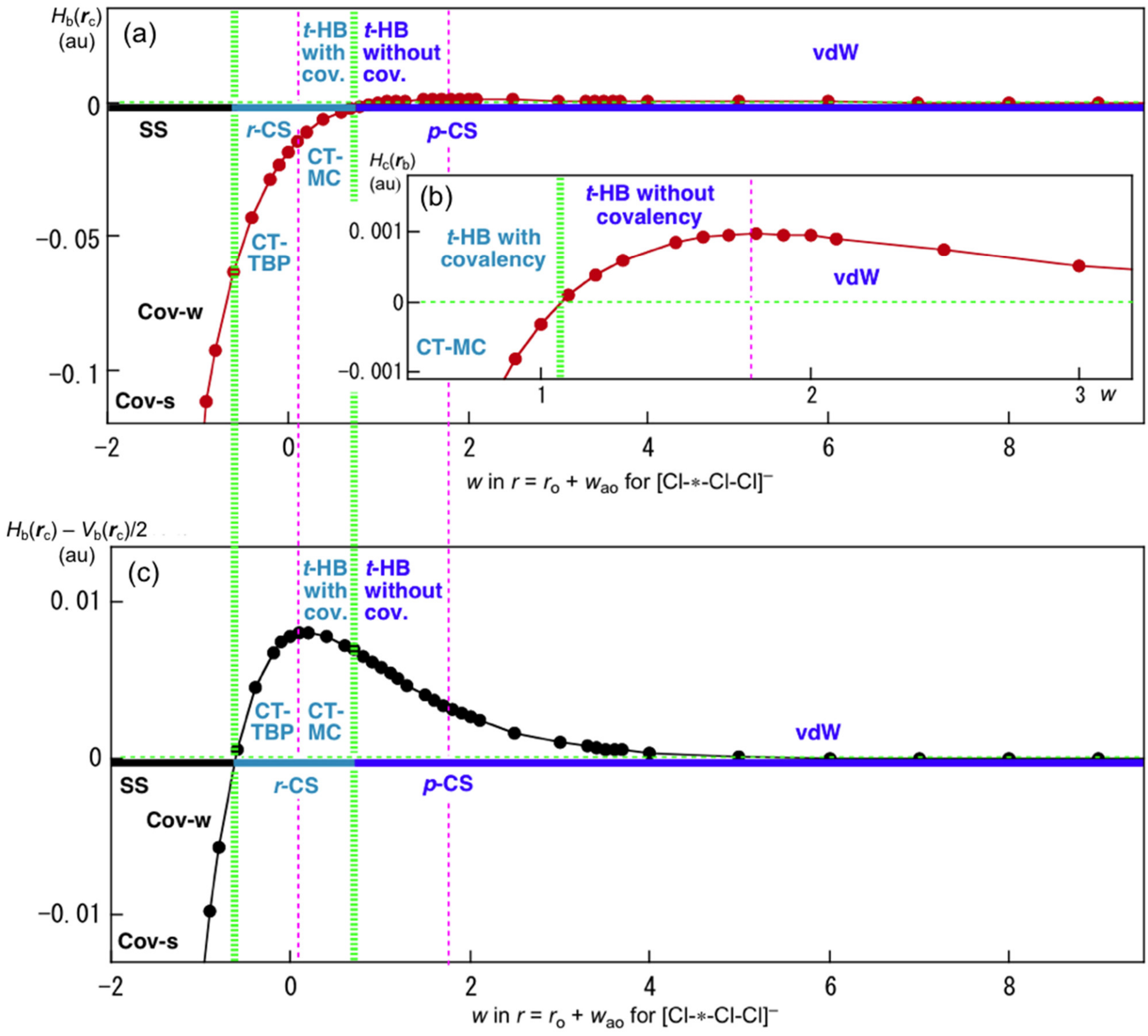


**Figure SA2.** Plot of $H_b(\mathbf{r}_c)$ versus $w$ in $r(^1Cl–^2Cl) = r_o(^1Cl–^2Cl) + wa_o$ for $^1Cl–^2Cl–^3Cl^-$ (a) with the magnified picture of (a) (b) and that of $H_b(\mathbf{r}_c) – V_b(\mathbf{r}_c)/2$ versus $w$ (c). Typical hydrogen bonds without covalency and typical hydrogen bonds with covalency are abbreviated as *t*–HB without cov. and *t*–HB with cov., respectively, whereas Cov–w and Cov–s stand for weak covalent bonds and strong covalent bonds, respectively.

**Table A1.** Proposed definitions for the classification and characterization of interactions by the signs $H_b(\boldsymbol{r}_c)$ and $H_b(\boldsymbol{r}_c) - V_b(\boldsymbol{r}_c)/2$ and their first derivatives, together with the tentatively proposed definitions by the characteristic points on the plots of $H_b(\boldsymbol{r}_c)$ versus $H_b(\boldsymbol{r}_c) - V_b(\boldsymbol{r}_c)/2$. The tentatively proposed definitions are shown by italic. The requirements for the interactions are also shown.

| ChP/Interaction | Requirements by $H_b(\boldsymbol{r}_c)$ and $V_b(\boldsymbol{r}_c)$ | Requirements by $G_b(\boldsymbol{r}_c)$ and $V_b(\boldsymbol{r}_c)$ |
|---|---|---|
| Origin | $H_b(\boldsymbol{r}_c) - V_b(\boldsymbol{r}_c)/2 = 0$; $H_b(\boldsymbol{r}_c) = 0$ | $G_b(\boldsymbol{r}_c) = 0$; $V_b(\boldsymbol{r}_c) = 0$ |
| vdW | $H_b(\boldsymbol{r}_c) > 0$; $\mathrm{d}H_b(\boldsymbol{r}_c)/\mathrm{d}(-r) > 0$ | $G_b(\boldsymbol{r}_c) > -V_b(\boldsymbol{r}_c)$; $\mathrm{d}G_b(\boldsymbol{r}_c)/\mathrm{d}(-r) > -\mathrm{d}V_b(\boldsymbol{r}_c)/\mathrm{d}(-r)$ |
| Borderline (BD–1) | $H_b(\boldsymbol{r}_c) > 0$; $\mathrm{d}H_b(\boldsymbol{r}_c)/\mathrm{d}(-r) = 0$ | $G_b(\boldsymbol{r}_c) > -V_b(\boldsymbol{r}_c)$; $\mathrm{d}G_b(\boldsymbol{r}_c)/\mathrm{d}(-r) = -\mathrm{d}V_b(\boldsymbol{r}_c)/\mathrm{d}(-r)$ |
| $t$–HB$_{\text{with no covalency}}$ | $H_b(\boldsymbol{r}_c) > 0$; $\mathrm{d}H_b(\boldsymbol{r}_c)/\mathrm{d}(-r) < 0$ | $G_b(\boldsymbol{r}_c) > -V_b(\boldsymbol{r}_c)$; $\mathrm{d}G_b(\boldsymbol{r}_c) < -\mathrm{d}V_b(\boldsymbol{r}_c)$ |
| Borderline ($x$–intercept) | $H_b(\boldsymbol{r}_c) = 0$ ($\theta_p{}^a = 125^{\circ}$) | $G_b(\boldsymbol{r}_c) = -V_b(\boldsymbol{r}_c)$ ($\theta_p{}^a = 125^{\circ}$) |
| $t$–HB$_{\text{with covalency}}$ | $H_b(\boldsymbol{r}_c) < 0$; ($125^{\circ} <$) $\theta_p{}^a < 150^{\circ}$ | $G_b(\boldsymbol{r}_c) < -V_b(\boldsymbol{r}_c)$; ($125^{\circ} <$) $\theta_p{}^b < 150^{\circ}$ |
| *Borderline (Tentative)* | *$\theta_p{}^a = 150^{\circ}$* | *$\theta_p{}^b = 150^{\circ}$* |
| CT–MC | $\mathrm{d}(H_b(\boldsymbol{r}_c) - V_b(\boldsymbol{r}_c)/2)/\mathrm{d}(-r) > 0$; | $\mathrm{d}G_b(\boldsymbol{r}_c) > \mathrm{d}V_b(\boldsymbol{r}_c)/2$; |
| | $150^{\circ} < \theta_p{}^a < 180^{\circ}$ | $150^{\circ} < \theta_p{}^a < 180^{\circ}$ |
| Borderline (BD–2) | $\mathrm{d}(H_b(\boldsymbol{r}_c) - V_b(\boldsymbol{r}_c)/2)/\mathrm{d}(-r) = 0$ | $2\mathrm{d}G_b(\boldsymbol{r}_c)/\mathrm{d}(-r) = -\mathrm{d}V_b(\boldsymbol{r}_c)/\mathrm{d}(-r)$ |
| | ($H_b(\boldsymbol{r}_c) - V_b(\boldsymbol{r}_c)/2 > 0$; $H_b(\boldsymbol{r}_c) < 0$) | ($-V_b(\boldsymbol{r}_c)/2 < G_b(\boldsymbol{r}_c) < -V_b(\boldsymbol{r}_c)$) |
| CT–TBP with $X_3^-$ | $\mathrm{d}(H_b(\boldsymbol{r}_c) - V_b(\boldsymbol{r}_c)/2)/\mathrm{d}(-r) < 0$ | $2\mathrm{d}G_b(\boldsymbol{r}_c)/\mathrm{d}(-r) < -\mathrm{d}V_b(\boldsymbol{r}_c)/\mathrm{d}(-r)$ |
| | ($H_b(\boldsymbol{r}_c) - V_b(\boldsymbol{r}_c)/2 > 0$; $H_b(\boldsymbol{r}_c) < 0$) | ($-V_b(\boldsymbol{r}_c)/2 < G_b(\boldsymbol{r}_c) < -V_b(\boldsymbol{r}_c)$) |
| Borderline ($y$–intercept) | $H_b(\boldsymbol{r}_c) - V_b(\boldsymbol{r}_c)/2 = 0$ ($H_b(\boldsymbol{r}_c) < 0$) | $G_b(\boldsymbol{r}_c) = -V_b(\boldsymbol{r}_c)/2$ ($G_b(\boldsymbol{r}_c) < -V_b(\boldsymbol{r}_c)$) |
| Cov–w | $H_b(\boldsymbol{r}_c) - V_b(\boldsymbol{r}_c)/2 < 0$; *$R^c < 0.15$ au* | $G_b(\boldsymbol{r}_c) < -V_b(\boldsymbol{r}_c)/2$; *$R^c < 0.15$ au* |
| *Borderline (Tentative)* | *$R^c = 0.15$ au* | *$R^d = 0.15$ au* |
| Cov–s | $H_b(\boldsymbol{r}_c) - V_b(\boldsymbol{r}_c)/2 < 0$; *$R^c > 0.15$ au* | $G_b(\boldsymbol{r}_c) < -V_b(\boldsymbol{r}_c)/2$; *$R^d > 0.15$ au* |

[a] $\theta_p = 90^{\circ} - \tan^{-1}[\mathrm{d}H_b(\boldsymbol{r}_c)/\mathrm{d}(H_b(\boldsymbol{r}_c) - V_b(\boldsymbol{r}_c)/2)]$, $\theta_p = 125^{\circ}$ is tentatively given for $\theta = 90^{\circ}$, where $\theta$ is defined by $90^{\circ} - \tan^{-1}[H_b(\boldsymbol{r}_c)/(H_b(\boldsymbol{r}_c) - V_b(\boldsymbol{r}_c)/2)]$ with $H_b(\boldsymbol{r}_c) = 0$. [b] $\theta_p = 90^{\circ} - \tan^{-1}[\mathrm{d}(G_b(\boldsymbol{r}_c) + V_b(\boldsymbol{r}_c))/\mathrm{d}(G_b(\boldsymbol{r}_c) + V_b(\boldsymbol{r}_c)/2)]$, $\theta_p = 125^{\circ}$ is tentatively given for $\theta = 90^{\circ}$, where $\theta$ is defined by $90^{\circ} - \tan^{-1}[(G_b(\boldsymbol{r}_c) + V_b(\boldsymbol{r}_c))/(G_b(\boldsymbol{r}_c) + V_b(\boldsymbol{r}_c)/2)]$ with $(G_b(\boldsymbol{r}_c) + V_b(\boldsymbol{r}_c)) = 0$]. [c] $R = [(H_b(\boldsymbol{r}_c) - V_b(\boldsymbol{r}_c)/2)^2 + (H_b(\boldsymbol{r}_c))^2]^{1/2}$. [d] $R = [(G_b(\boldsymbol{r}_c) + V_b(\boldsymbol{r}_c)/2)^2 + (G_b(\boldsymbol{r}_c) + V_b(\boldsymbol{r}_c))^2]^{1/2}$.

**References**

SA1 (a) *Atoms in Molecules. A Quantum Theory*: eds. R. F. W. Bader, Oxford University Press, Oxford, UK, 1990; (b) C. F. Matta, R. J. Boyd, *An Introduction to the Quantum Theory of Atoms in Molecules* in *The Quantum Theory of Atoms in Molecules: From Solid State to DNA and Drug Design*: eds. C. F. Matta, R. J. Boyd, WILEY–VCH, Weinheim, Germany, 2007, Chapter 1.

SA2 (a) R. F. W. Bader, T. S. Slee. D. Cremer, E. Kraka, *J. Am. Chem. Soc.* **1983**, *105*, 5061–5068; (b) R. F. W. Bader, *Chem. Rev.* **1991**, *91*, 893–926; (c) R. F. W. Bader, *J. Phys. Chem. A* **1998**, *102*, 7314–7323; (d) F. Biegler–König, R. F. W. Bader, T. H. Tang, *J. Comput. Chem.* **1982**, **3**, 317–328; (e) R. F. W. Bader, *Acc. Chem. Res.* **1985**, *18*, 9–15; (f) T. H. Tang, R. F. W. Bader, P. MacDougall, *Inorg. Chem.* **1985**, *24*, 2047–2053; (g) F. Biegler–König, J. Schönbohm, D. Bayles, *J. Comput. Chem.* **2001**, *22*, 545–559; (h) F. Biegler–König, J. Schönbohm, *J. Comput. Chem.* **2002**, *23*, 1489–1494.

SA3 W. Nakanishi, T. Nakamoto, S. Hayashi, T. Sasamori, N. Tokitoh, *Chem. Eur. J.* **2007**, *13*, 255–268.

SA4 (a) W. Nakanishi, S. Hayashi, K. Narahara, *J. Phys. Chem. A* **2009**, *113*, 10050–10057; (*b*) W. Nakanishi, S. Hayashi, K. Narahara, *J. Phys. Chem. A* **2008**, *112*, 13593–13599.

SA5 W. Nakanishi, S. Hayashi, *Curr. Org. Chem.* **2010**, *14*, 181–197.

SA6 (a) W. Nakanishi, S. Hayashi, *J. Phys. Chem. A* **2010**, *114*, 7423–7430; (b) W. Nakanishi, S. Hayashi, K. Matsuiwa, M. Kitamoto, *Bull. Chem. Soc. Jpn* **2012**, *85*, 1293–1305.

SA7 W. Grimme, J. Wortmann, D. Frowein, J. Lex, G. Chen, R. Gleiter, *J. Chem. Soc., Perkin Trans. 2* **1998,** 1893–1900.

SA8 C.–T. Lin, N.–J. Wang, Y.–L. Yeh, T.–C. Chou, Tetrahedron, **1995**, *51*, 2907–2928.

SA9 E. Espinosa, I. Alkorta, J. Elguero, E. Molins, *J. Chem. Phys.* **2002**, *117*, 5529. See also R. Bianchi, G. Gervasio and D. Marabello, *C. R. Chim.*, 2005, **8**, 1392.
